# Functional Prokaryotic-Like Deoxycytidine Triphosphate Deaminases and Thymidylate Synthase in Eukaryotic Social Amoebae: Vertical, Endosymbiotic, or Horizontal Gene Transfer?

**DOI:** 10.1093/molbev/msad268

**Published:** 2023-12-08

**Authors:** Heng Liang, Jeffrey P Mower, Catherine P Chia

**Affiliations:** School of Biological Sciences, University of Nebraska-Lincoln, Lincoln, NE 68588, USA; Department of Agronomy and Horticulture, University of Nebraska-Lincoln, Lincoln, NE 68588, USA; School of Biological Sciences, University of Nebraska-Lincoln, Lincoln, NE 68588, USA

**Keywords:** dTTP synthesis, dictyostelids, lateral gene transfer, in vivo functionality, phylogeny, soil microorganisms

## Abstract

The de novo synthesis of deoxythymidine triphosphate uses several pathways: gram-negative bacteria use deoxycytidine triphosphate deaminase to convert deoxycytidine triphosphate into deoxyuridine triphosphate, whereas eukaryotes and gram-positive bacteria instead use deoxycytidine monophosphate deaminase to transform deoxycytidine monophosphate to deoxyuridine monophosphate. It is then unusual that in addition to deoxycytidine monophosphate deaminases, the eukaryote *Dictyostelium discoideum* has 2 deoxycytidine triphosphate deaminases (Dcd1_Dicty_ and Dcd2_Dicty_). Expression of either Dcd_Dicty_ can fully rescue the slow growth of an *Escherichia coli dcd* knockout. Both Dcd_Dicty_ mitigate the hydroxyurea sensitivity of a *Schizosaccharomyces pombe* deoxycytidine monophosphate deaminase knockout. Phylogenies show that Dcd1_Dicty_ homologs may have entered the common ancestor of the eukaryotic groups of Amoebozoa, Obazoa, Metamonada, and Discoba through an ancient horizontal gene transfer from a prokaryote or an ancient endosymbiotic gene transfer from a mitochondrion, followed by horizontal gene transfer from Amoebozoa to several other unrelated groups of eukaryotes. In contrast, the Dcd2_Dicty_ homologs were a separate horizontal gene transfer from a prokaryote or a virus into either Amoebozoa or Rhizaria, followed by a horizontal gene transfer between them. ThyX_Dicty_, the *D. discoideum* thymidylate synthase, another enzyme of the deoxythymidine triphosphate biosynthesis pathway, was suggested previously to be acquired from the ancestral mitochondria or by horizontal gene transfer from alpha-proteobacteria. ThyX_Dicty_ can fully rescue the *E. coli* thymidylate synthase knockout, and we establish that it was obtained by the common ancestor of social amoebae not from mitochondria but from a bacterium. We propose horizontal gene transfer and endosymbiotic gene transfer contributed to the enzyme diversity of the deoxythymidine triphosphate synthesis pathway in most social amoebae, many Amoebozoa, and other eukaryotes.

## Introduction

**Fig. 1.**
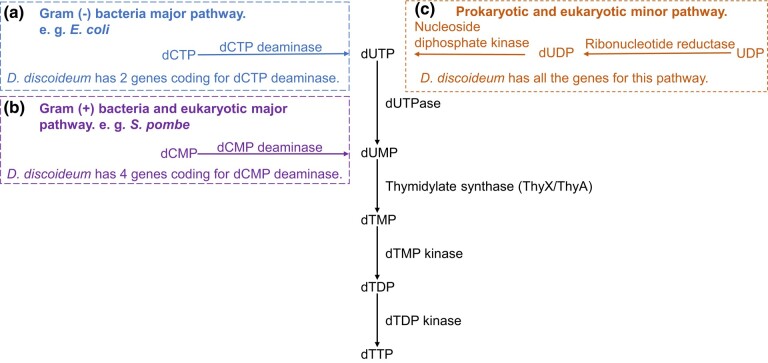
De novo synthesis of dTTP in prokaryotes and eukaryotes (salvage pathway not shown). a) The Gram-negative bacteria major pathway begins with the deamination of dCTP to dUTP by dCTP deaminase dUTP is hydrolyzed to dUMP by dUTPase. b) The Gram-positive bacteria and eukaryotic major pathway starts with the deamination of dCMP to dUMP by dCMP deaminase. c) The prokaryotic and eukaryotic minor pathway uses ribonucleotide reductase to reduce UDP to dUDP (or UTP to dUTP; not shown) followed by dUTP hydrolysis to dUMP by dUTPase. The conversion of dUMP to dTTP is common to all 3 pathways. TS converts dUMP to dTMP. Most eukaryotes use ThyA, except social amoebae which use ThyX, while bacteria use either ThyA or ThyX.

In modern cells, deoxyadenosine triphosphate (dATP), deoxyguanosine triphosphate (dGTP), and deoxycytidine triphosphate (dCTP) are made from their immediate RNA precursors by the action of ribonucleotide reductase on their respective ribonucleoside triphosphates (NTPs) (Reichard et al. 1961). Compared with these 3 deoxyribonucleotides, deoxythymidine triphosphate (dTTP) does not have an immediate NTP precursor and has a more complex biosynthetic pathway. An important intermediate in the de novo synthesis of dTTP is deoxyuridine monophosphate (dUMP) that is generated by major and minor pathways (Adams et al. 1992) (Fig. 1). Common to both prokaryotes and eukaryotes is the minor pathway that uses ribonucleotide reductase to reduce uridine diphosphate (UDP) to deoxyuridine diphosphate (dUDP) (or uridine triphosphate [UTP] to deoxyuridine triphosphate [dUTP]). The other pathway, commonly called the major pathway because it provides more than 80% of dTTP in V79 CHO cell lines (Bianchi et al. 1987), starts with either deoxycytidine monophosphate (dCMP) or dCTP. In gram-negative bacteria, the major pathway starts with the deamination of dCTP to dUTP by dCTP deaminase. The dCTP deaminases are homotrimers that bind magnesium and are members of the same trimeric dUTPase superfamily (Koonin 1996; Johansson et al. 2005; Vértessy and Tóth 2009). Though not lethal, the dCTP deaminase knockout of *Escherichia coli* (Δ*dcd E. coli*) grows poorly in the absence of thymidine, indicating that neither the remaining minor de novo dTTP synthesis nor salvage pathways produce sufficient dTTP for normal cell growth (Reichard 1988; Baba et al. 2006; Weiss 2007).

In gram-positive bacteria and eukaryotes, the major pathway for dTTP synthesis starts with the deamination of dCMP to dUMP by dCMP deaminase. As zinc ion–based hexamers (Hou et al. 2008), dCMP deaminases have a different structure from and are unrelated to dCTP deaminases. *Schizosaccharomyces pombe* cells with dCMP deaminase knocked out (Δ*S. pombe*) have a lengthened cell cycle because DNA replication is hindered. These cells also are more sensitive to hydroxyurea (HU), an inhibitor of ribonucleotide reductase that severely impairs DNA synthesis, with the consequence of a prolonged S phase (Sánchez et al. 2012). Since yeasts lack the thymidine salvage pathway (Grivell and Jackson 1968), the slow growth phenotype of Δ*S. pombe* cells indicates the minor de novo dTTP synthesis pathway is insufficient for normal cell growth.

Another major component of de novo dTTP synthesis is thymidylate synthase (TS), which converts dUMP to dTMP. Present in most eukaryotes and some bacteria is the ThyA type of TS (EC 2.1.1.45) which uses tetrahydrofolate as a reductant. Present in other bacteria is a ThyX type of TS (EC 2.1.1.148) that uses flavin coenzymes to deliver reducing equivalents to dUMP. Although both ThyA and ThyX catalyze the synthesis of thymidylate, their structures and enzymatic mechanisms are distinctly different (Myllykallio et al. 2002). *Escherichia coli* with an interrupted *thyA* show a phenotype of thymine-dependent growth (Michaels et al. 1990), and when transferred from media with thymine to media without thymine, the Δ*thyA E. coli* cells become elongated filaments (Escartin et al. 2008). Previously studied bacterial and viral thyX enzymes are unable to revert the Δ*thyA E. coli* back to wild-type (WT) growth and shape (Escartin et al. 2008).

Social amoebae, including the well-studied *Dictyostelium discoideum*, are soil microbes that are divided into 4 groups based on morphological characteristics and molecular phylogenetic analyses of ribosomal RNA (Schaap et al. 2006). They are intriguing eukaryotes to examine the evolution of pathways of dTTP synthesis because the genomes of many social amoebae are A + T rich. For example, the nuclear and mitochondrial genomes of *D. discoideum* are 77.5% and 72.65% AT, respectively (Ogawa et al. 2000; Eichinger et al. 2005). The genomes require substantial amounts of dTTP during mitotic cell growth, as well as during starvation-triggered multicellular development when mitochondrial DNA replication is observed to occur (Shaulsky and Loomis 1995).

Social amoebae have the minor pathway with ribonucleotide reductase to reduce UDP to dUDP. They also have the eukaryotic major pathway that uses dCMP deaminase to turn dCMP to dUMP. According to DictyExpress, 4 genes coding for dCMP deaminases are expressed in 3 different patterns during starvation-induced development of *D. discoideum* (supplementary fig. S1, Supplementary Material online; Stajdohar et al. 2017). Unexpectedly, 2 genes annotated to code for prokaryote-like dCTP deaminases also are present in the genome of *D. discoideum* (*dcd1_Dicty_* DDB_G0293580 and *dcd2_Dicty_* DDB_G0268194), and presumably, their expression creates a second route to synthesize dTTP. Confirming their expression, both dCTP deaminases are detected in a recent proteomic study of vegetative *D. discoideum* cells (Freitas et al. 2022). From published transcriptomic studies (Parikh et al. 2010), *dcd1_Dicty_* matches the expression pattern of *dCMP deaminase 3*, which increases during development, while *dcd2_Dicty_* matches the expression pattern of *dCMP deaminase 1* and *2*, which drops throughout the entire development period. These patterns suggest a coordination of the dCMP deaminases with the dCTP deaminases.

Also, within the dTTP synthesis pathway, rather than the expected eukaryotic ThyA, a gene coding for ThyX was annotated instead (DDB_G0280045) (Eichinger et al. 2005). The *thyX_Dicty_* was identified as gained either through horizontal gene transfer (HGT) or mitochondrial gene transfer (Stern et al. 2010). Its expression pattern largely matches those of *dcd1_Dicty_* and *dCMP deaminase 3* (supplementary fig. S1, Supplementary Material online).

In *D. discoideum*, 2 enzymes contributing to dTTP biosynthesis have been biochemically characterized: dUTPase (Chia et al. 2020) and ribonucleotide reductases (Crona et al. 2013). The function of ThyX_Dicty_ was inferred by its rescue of a thymidine-dependent mutant strain of *D. discoideum* (Dynes and Firtel 1989; Escartin et al. 2008). One of the *D. discoideum* ribonucleotide reductases is bacterial in origin, and ThyX_Dicty_ is either prokaryotic or mitochondrial in origin (Stern et al. 2010; Crona et al. 2013). Most of the remaining enzymes in the de novo dTTP synthesis pathway appear to have eukaryotic origins and are universally present in eukaryotes except for the prokaryotic-like dCTP deaminases (Fig. 1). The functions of the predicted prokaryotic-like Dcd1_Dicty_ and Dcd2_Dicty_ are unverified.

In this study, we establish the functionality of the presumptive dCTP deaminases in both a prokaryote and a eukaryote. In growth experiments, *dcd1_Dicty_* and *dcd2_Dicty_* each can separately successfully complement a *dcd* knockout of *E. coli*. Both can relieve the sensitivity of a dCMP deaminase knockout of *S. pombe* toward HU. The *thyX_Dicty_* can rescue the slow growth phenotype of the *thyA* knockout of *E. coli* and convert the filament-forming cells of the knockout to a normal rod shape. Through phylogenetic analyses, we examine the evolutionary histories and origins of the presumptive prokaryotic *dcd* and *thyX* genes in social amoebae and other eukaryotes. We conclude *dcd1_Dicty_* is older than *dcd2_Dicty_* and that these 2 dCTP deaminases in amoebae have different origins. The *dcd1_Dicty_* was gained either from HGT from prokaryotes, or endosymbiotic gene transfer (EGT) from mitochondria, with HGT more likely, while *dcd2_Dicty_* was gained from HGT from prokaryotes or viruses. For *thyX_Dicty_*, analyses support that another independent HGT from bacteria is responsible for replacing the expected *thyA* in a subset of Amoebozoa (social amoeba) and indicate that this event is younger than each of the HGT events of *dcd1_Dicty_* and *dcd2_Dicty_*.

## Results

### Expressed Dcd1_Dicty_ and Dcd2_Dicty_ Proteins Successfully Restore WT Growth to Δ*dcd E. coli*

**Fig. 2.**
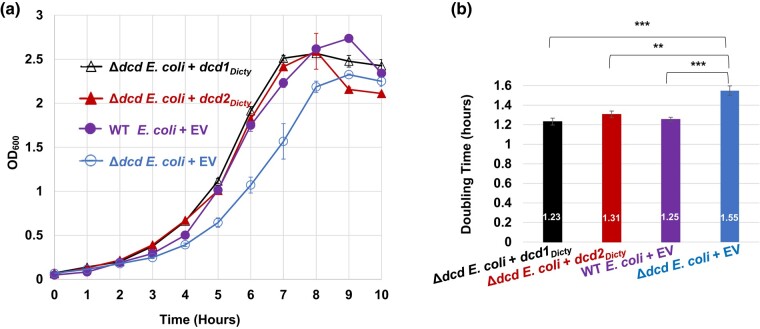
Growth of *E. coli* transformants and doubling time comparisons. The *E. coli* strains are the dCTP deaminase knockout of *E. coli* (Δ*dcd E. coli*) transformed with either *dcd1_Dicty_*, *dcd2_Dicty_*, or EV (Δ*dcd E. coli* + EV) and WT *E. coli* transformed with EV (WT *E. coli* + EV). a) Growth of transformants in MOPS minimal media. b) Calculated doubling times (numbers shown inside the bars). The doubling times of WT, *dcd1_Dicty_*, and *dcd2_Dicty_* are insignificantly different from each other, while all 3 strains have significantly better growth compared with Δ*dcd E. coli* + EV. The results are the means of at least 4 replicates. *P* ≤ 0.05 (*) indicates a significant difference; *P* ≤ 0.01 (**) and *P* ≤ 0.001 (***) indicate a highly significant difference. Error bars for both growth data and doubling times represent standard errors (in (a), solid symbols obscure small error bars).

We tested the function of the predicted *D. discoideum* dCTP deaminase proteins by expressing separately Dcd1_Dicty_ and Dcd2_Dicty_, each with C-terminal His_6_ tags, in Δ*dcd E. coli* (Fig. 2). Expression was confirmed by immunoblotting (supplementary fig. S2a, Supplementary Material online). When inoculated into prewarmed, preaerated media and grown at 37°C (warm start), Δ*dcd E. coli* cells transformed with *dcd1_Dicty_* or *dcd2_Dicty_* (Δ*dcd E. coli* + *dcd1_Dicty_*, Δ*dcd E. coli* + *dcd2_Dicty_*) grow as well as WT *E. coli* transformed with the empty vector (WT *E. coli* + EV; Fig. 2a). In comparison, and over multiple trials, the control Δ*dcd E. coli* transformed with the EV (Δ*dcd E. coli* + EV) grows poorly, consistent with the originally reported slower growth of Δ*dcd E. coli* compared with its WT parent (Baba et al. 2006). The doubling times of the Δ*dcd E. coli*  *+ dcd1_Dicty_*, Δ*dcd E. coli*  *+ dcd2_Dicty_*, and WT *E. coli* + EV are not statistically different from each other (*P* = 0.26; Fig. 2b). The doubling time of the Δ*dcd E. coli* + EV is 1.55 h, statistically different from the doubling times of the other transformants (*P* < 0.003; Fig. 2b). Because the Δ*dcd E. coli* cells expressing either of the 2 *D. discoideum* dCTP deaminases have growth comparable with the WT *E. coli* possessing its native enzyme, the data indicate that both Dcd1_Dicty_ and Dcd2_Dicty_ are functional dCTP deaminases compatible with gram-negative bacteria such as *E. coli*.

### Expressed *Dcd2_Dicty_* Improves the Growth of Δ*S. pombe* and Both *Dcd1_Dicty_* and *Dcd2_Dicty_* Partially Mitigate the HU-Inhibited Growth of Δ*S. pombe*

**Fig. 3.**
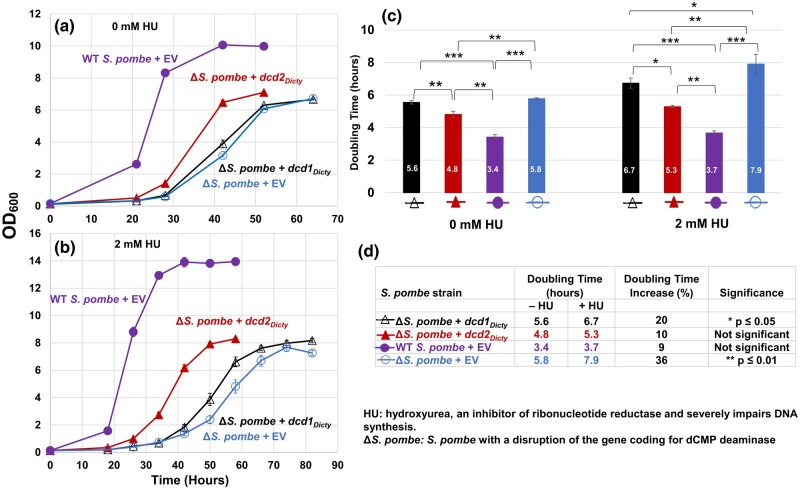
Growth of WT *S. pombe* and Δ*S. pombe* transformants and doubling time comparisons. The *S. pombe* strains are the dCMP deaminase knockout (Δ*S. pombe*) transformed with either *dcd1_Dicty_*, *dcd2_Dicty_*, or EV and WT *S. pombe* transformed with EV (WT *S. pombe* + EV). a) Growth in the absence of HU. b) Growth in the presence of 2 mM HU. All transformants grown in 2 mM HU form clusters upon reaching stationary phase because some of the mother and daughter cells do not separate and create chain-like structures, as documented previously (Tripathi et al. 2011). These likely contributed to the higher OD_600_ readings for stationary phase cells grown with 2 mM HU compared with cells grown without HU. c) Calculated doubling times (numbers inside bars). In the absence and presence of HU, doubling time differences among all pairs are significant. An exception was the Δ*S. pombe* + *dcd1_Dicty_* and Δ*S. pombe* + EV pairwise comparison without HU. d) HU has different impacts on the doubling times of *S. pombe* strains. The % increase was calculated (difference in doubling time)/(doubling time in the absence of HU). The results are means of a minimum of 3 replicates. *P* ≤ 0.05 (*) indicates a significant difference; *P* ≤ 0.01 (**) and *P* ≤ 0.001 (***) indicate highly significant differences. Error bars for both growth data and doubling times represent standard errors (in (a) and (b), solid symbols obscure small error bars).

With the evidence that the 2 dCTP deaminases from *D. discoideum* function in *E. coli*, we tested whether these enzymes typical of the major gram-negative prokaryotic pathway can substitute for the dCMP deaminase absent from Δ*S. pombe*. Untransformed Δ*S. pombe* grow slower in minimal liquid media compared with the growth of WT *S. pombe* (supplementary fig. S3, Supplementary Material online). We transformed Δ*S. pombe* with the EV or *dcd1_Dicty_* or *dcd2_Dicty_* (Δ*S. pombe* + EV, Δ*S. pombe* + *dcd1_Dicty_*, Δ*S. pombe* + *dcd2_Dicty_*) and WT *S. pombe* with EV (WT *S. pombe* + EV) and monitored their growth. Expression of the full-length His-tagged proteins was verified (supplementary fig. S2b, Supplementary Material online). Growth studies (Fig. 3a and c) show that compared with Δ*S. pombe +* EV, the *dcd1_Dicty_* strain barely decreases the doubling time, while the *dcd2_Dicty_* strain has improved growth, reducing the doubling time from 5.8 to 4.8 h (17%). These data indicate that while *dcd1_Dicty_* has little impact, *dcd2_Dicty_* can partially improve the growth of the Δ*S. pombe*.

HU is a ribonucleotide reductase inhibitor that decreases the production of deoxyribonucleoside triphosphates (dNTPs) and thus causes a lengthening of the S phase of the cell cycle (Krakoff et al. 1968). In Δ*S. pombe*, sublethal doses of HU cause the collapse of the replication fork that consequently destabilizes the genome (Sánchez et al. 2012). In the presence of HU, the Δ*S. pombe* strains expressing Dcd1_Dicty_ and Dcd2_Dicty_ (supplementary fig. S2b, Supplementary Material online) both show partial and statistically significant improvement in growth compared with the knockout strain with EV (Fig. 3b and c). Although the doubling times of all strains increased in the presence of HU, the doubling times of Δ*S. pombe* transformed with *dcd1_Dicty_* or *dcd2_Dicty_* are still significantly shorter than that of Δ*S. pombe* + EV (Fig. 3c). Neither (*dcd1_Dicty_* nor *dcd2_Dicty_*) transformant, however, restores the Δ*S. pombe* to the level of WT + EV. When comparing the growth of the same strains with and without HU (Fig. 3d), the transformants were less affected by HU than the Δ*S. pombe* + EV. Δ*S. pombe* + *dcd2_Dicty_* had only a 10% increase in doubling time (statistically insignificant), and Δ*S. pombe* + *dcd1_Dicty_* had a 20% increase, suggesting that the *D. discoideum* dCTP deaminase proteins ameliorate the damage of HU by providing another source of dUMP in the absence of the native dCMP deaminase.

### Expressed ThyX_Dicty_ Successfully Restores Δ*thyA E. coli* to WT Growth and Normal Rod Shapes

**Fig. 4.**
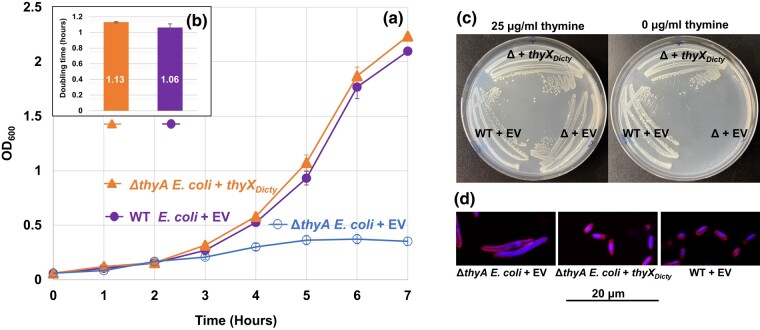
Growth of *E. coli* transformants and doubling time comparisons. The *E. coli* strains are the TS gene (*thyA*) knockout of *E. coli* (Δ*thyA E. coli*) transformed with either *thyX_Dicty_* or EV (Δ*thyA E. coli* + EV) and WT *E. coli* transformed with EV (WT *E. coli* + EV). a) Growth of transformants in MOPS minimal media. b) Calculated doubling times (numbers shown inside the bars). The doubling times of WT + EV and Δ*thyA E. coli*  *+*  *thyX_Dicty_* are insignificantly different from each other. The Δ*thyA E. coli* + EV stopped growing after reaching OD_600_ = 0.36, which did not permit a doubling time calculation. The results are the means of 3 replicates. Error bars for both growth data and doubling times represent standard errors (in (a), solid symbols obscure small error bars). c) Complementation of Δ*thyA E. coli* by *thyX_Dicty_*. Cells from well-isolated single colonies streaked on MOPS minimal media with (left) or without (right) thymine. Colonies of both Δ*thyA E. coli* + *thyX_Dicty_* and WT *E. coli* + EV arise at similar rates (images taken after 36 h at 37˚C), while Δ*thyA E. coli +* EV grows only in the presence of thymine. d) Dual staining of *E. coli* cells grown under thymine deprivation. The cell membrane is stained with FM4-64 and DNA is stained with DAPI. The figure shows a representative field of the filamentous cells observed for Δ*thyA E. coli* + EV, whereas the Δ*thyA E. coli +*  *thyX_Dicty_* transformant and WT + EV strains do not form filaments.

We tested the function of the predicted *D. discoideum* TS (flavin-dependent) by expressing ThyX_Dicty_ with a C-terminal His_6_ tag, in Δ*thyA E. coli* (Fig. 4). Expression was confirmed by immunoblotting (data not shown). The Δ*thyA E. coli* cells transformed with *thyX* (Δ*thyA E. coli* + *thyX_Dicty_*) grow as well as WT *E. coli* transformed with the EV (WT *E. coli* + EV; Fig. 4a). In comparison, and over multiple trials, the control Δ*thyA E. coli* transformed with the EV (Δ*thyA E. coli* + EV) ceases to increase beyond OD_600_ of 0.36, presumably due to the depletion of thymine. The average doubling times of the Δ*thyA E. coli*  *+ thyX_Dicty_* and WT *E. coli* + EV are 1.06 and 1.13 h, respectively (Fig. 4b), which are not statistically different from each other (*P* = 0.20). A meaningful doubling time for Δ*thyA E. coli* + EV could not be calculated as cells do not reach log phase. While the filamentous phenotype of Δ*thyA E. coli* in liquid cultures may distort cell numbers as measured by the OD_600_ (Stevenson et al. 2016), the lack of growth of the mutant on solid media in the absence of thymine (Fig. 4c) implies lengthy doubling times if any division occurs.

Because the Δ*thyA E. coli* cells expressing the *D. discoideum* ThyX_Dicty_ have growth comparable with the WT *E. coli* possessing its native ThyA, the data indicate that ThyX_Dicty_ is a functional TS compatible with gram-negative bacteria such as *E. coli*. On solid media, the Δ*E. coli* + *thyX_Dicty_* and WT *E. coli* + EV exhibit similar growth with or without thymine, while the Δ*E. coli* + EV does not grow in the absence of thymine (Fig. 4c). In addition, ThyX_Dicty_ can rescue the filamentous phenotype of Δ*thyA E. coli* (Fig. 4d). The EV transformants of Δ*thyA E. coli* show elongated cells, while the Δ*thyA E. coli* + *thyX_Dicty_* and WT *E. coli* + EV have normal rod shapes that are 1.5 µm long and 0.5 µm wide.

### Phylogenetic Analyses of Eukaryotic Dcd Homologs

**Fig. 5.**
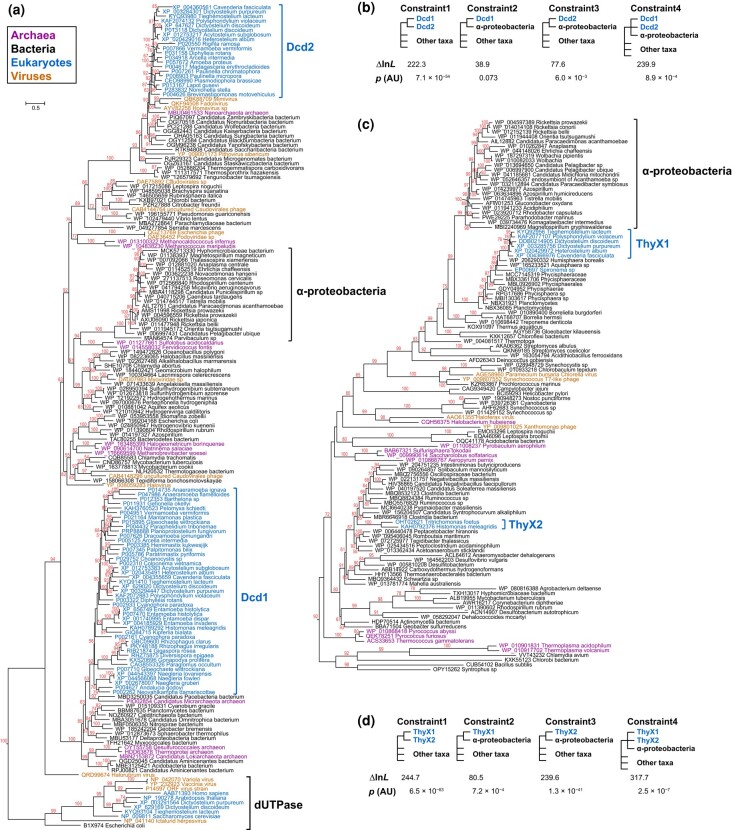
Phylogenetic trees for Dcd and ThyX and the topology tests for the likelihood of alternative hypotheses. a) The tree from ML evaluation of 179 sequences including 168 dCTP deaminase protein sequences from diverse species and 11 dUTPases. The 11 dUTPase sequences were used to root the tree. Taxa are labeled with protein IDs followed by names of species. Taxon names are color-coded according to biological classification: bacteria, black; eukaryotes, blue; archaea, purple; viruses, orange. Bootstrap values ≥50% from 1,000 replicates are shown above branches. Scale bar (substitutions per site) is shown at the top left. b) Alternative topology tests for Dcd1 and Dcd2. Different constraints representing alternative topology hypotheses are demonstrated in cladograms. From left to right, constraints 1 to 4 represent the alternative hypotheses: Dcd1 and Dcd2 entered eukaryotes through 1 event; Dcd1 is from mitochondrial gene transfer; Dcd2 is from mitochondrial gene transfer; Dcd1 and Dcd2 are from the same mitochondrial gene transfer event. The natural log of the likelihood (ln*L*) of the alternative hypotheses is compared with the ln*L* of the ML tree, and the difference is labeled (Δln*L*); *P*(AU) < 0.05 means an alternative hypothesis is rejected. All alternative hypotheses are rejected except for constraint 2. c) The tree from the ML evaluation of 116 flavin-dependent TS protein sequences from diverse species. Midpoint rooting of the tree is used because there is no obvious outgroup for ThyX. ThyX1 indicates ThyX homologs of social amoebae. ThyX2 indicates ThyX homologs of metamonads (*Tritrichomonas* and *Histomonas*). The tree is labeled, color-coded, and scaled as described in (a). d) Alternative topology tests for ThyX. Different constraints representing alternative topology hypotheses are demonstrated in cladograms. From left to right, constraints 1 to 4 represent the alternative hypotheses: ThyX1 and ThyX2 entered eukaryotes through 1 event; ThyX1 is from mitochondrial gene transfer; ThyX2 is from mitochondrial gene transfer; ThyX1 and ThyX2 are from the same mitochondrial gene transfer event. All the alternative hypotheses are rejected (*P* < 0.001). Details of the values and statistical tests are described in (b) (see also supplementary material and methods, Supplementary Material online). Constraints and the corresponding fully resolved trees for topology tests of Dcd and ThyX are shown in supplementary fig. S11, Supplementary Material online.

The presence of functional prokaryotic-like dCTP deaminases in *D. discoideum* prompts an investigation into their evolutionary origins, beginning with a search for homologs in other eukaryotes. The presence of Dcd1 and Dcd2 in eukaryotes is shown in a detailed tree (196 species) that is reduced to a 34-taxa tree (supplementary fig. S4a and b, Supplementary Material online). We next took Dcd sequences from representative clades of bacteria. A third set of sequences were from a general BLASTp search using Dcd1_Dicty_ and Dcd2_Dicty_ as separate queries. The resulting phylogenetic analysis of 235 Dcd homologs was generated using maximum likelihood (ML) (supplementary fig. S5, Supplementary Material online), which was trimmed to build an ML tree of 179 species (Fig. 5a) (see supplementary material and methods, Supplementary Material online).

**Fig. 6.**
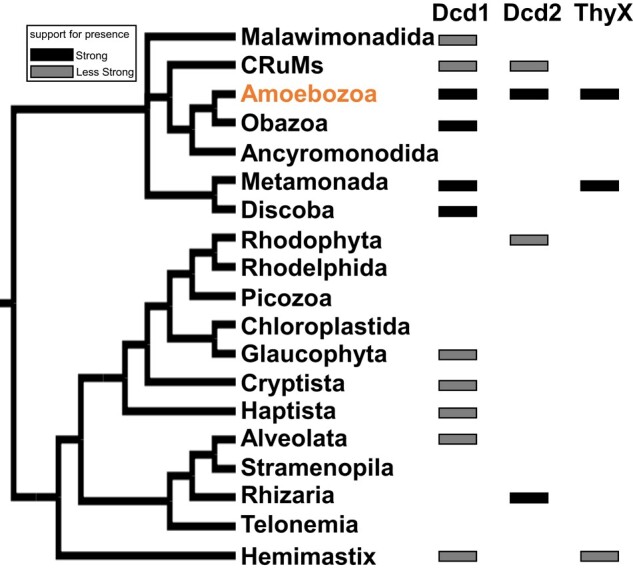
The distribution of Dcd1, Dcd2, and ThyX among 19 major eukaryotic groups. The simplified tree was derived from 4 studies of the phylogeny of eukaryotes (Brown et al. 2018; Burki et al. 2020; Schön et al. 2021; Strassert et al. 2021). A solid black rectangle means that the predicted protein is present in multiple (but not necessarily all) species in the same major eukaryotic group that form a monophyletic clade in their protein phylogeny (supplementary fig. S6, Supplementary Material online). A gray rectangle means either there is only a single species in the major group that contains the predicted protein or the presence of species belonging to the same major eukaryotic group that do not form a monophyletic clade. The detailed species distribution of Dcd1, Dcd2, and ThyX in the eukaryotic clades of their respective protein phylogenies is shown in supplementary fig. S6, Supplementary Material online.

The eukaryotic Dcd1 homologs form a monophyletic clade with strong Bootstrap branch support (BS = 92%). The clade includes multiple annotated genome sequences from diverse Amoebozoa, plus Obazoa, Metamonada, and Discoba species, which are systematically related eukaryotic groups sharing a common ancestor, as well as sequences from transcriptome studies of Malawimonadida, CRuMs, Glaucophyta, Cryptista, Haptista, Alveolata, and Hemimastix (Fig. 6, supplementary fig. S6a, Supplementary Material online). The whole eukaryotic clade was nested with strong support (BS = 100%) within a broader clade of sequences, with taxonomic affinities of mostly bacteria and some archaea. A distinct monophyletic clade of eukaryotic Dcd2 sequences had moderate support (BS = 63%) and included multiple annotated genome sequences of Amoebozoa and Rhizaria and sequences from transcriptome studies of CRuMs and Rhodophyta (Fig. 6, supplementary fig. S6b, Supplementary Material online). Indicating viral or prokaryotic origins, this whole eukaryotic Dcd2 clade is a sister group with giant viruses with medium support (BS = 63%), and this joint clade is nested with strong support (BS ranging from 93% to 100%) at multiple nodes inside a clade made up of mostly CPR bacteria and some archaea. These distinct phylogenetic positions of eukaryotic Dcd1 and Dcd2 clades indicate that Dcd1 and Dcd2 are distant paralogs with independent evolutionary origins.

Other possible evolutionary hypotheses regarding the origins of the genes encoding Dcd1 and Dcd2 are tested with different tree topologies. The alternative topology forcing Dcd1 and Dcd2 to be monophyletic with one another was rejected by the approximately unbiased (AU) test (*P* << 0.001; constraint 1; Fig. 5b), confirming their separate origins. Alternative topologies forcing the monophyly of alpha-proteobacteria with either Dcd1 or Dcd2 or Dcd1 and Dcd2 together tested the hypotheses of mitochondrial gene transfer (constraints 2 to 4; Fig. 5b). The topology grouping Dcd1 eukaryotic homologs with alpha-proteobacteria is statistically insignificant (constraint 2; *P*  *=* 0.073). Thus, the topology test cannot reject the possibility that *dcd1* could be from endosymbiotic (mitochondrial) gene transfer (EGT). The topology grouping Dcd2 with alpha-proteobacteria (constraint 3) and the topology grouping Dcd1, Dcd2, and alpha-proteobacteria together (constraint 4) are both rejected (*P* < 0.01) by the AU test. Thus, a mitochondrial origin for Dcd2 itself, or for Dcd1 and Dcd2 together, is rejected.

### Phylogenetic Analyses of Eukaryotic ThyX Homologs

A phylogenetic analysis of 174 detected ThyX sequences is shown in an ML tree (supplementary fig. S7, Supplementary Material online) that was trimmed to make a 116-sequence ML tree (Fig. 5c). Two distinct clades of eukaryotes were revealed. All social amoebae were clearly monophyletic (ThyX1; BS = 100%) grouping together with 2 planctomycetes (*Humisphaera borealis*, WP_206290332, and *Aquisphaera*, WP_165233521) that then group with *Spironema* (P015127) and firmly nested within Planctomycetes (BS ranging from 85% to 100%). The presence of ThyX1 in Amoebozoa and *Spironema* may be explained by separate HGTs from Planctomycetes to Amoebozoa and *Spironema*. A monophyletic metamonad clade was placed within a clade of Firmicutes from class Clostridia (ThyX2; BS = 83%). The AU alternative topology test rejected (*P* << 0.001) the shared origin of social amoebae (ThyX1) and metamonad (ThyX2) sequences (constraint 1; Fig. 5d). When grouping ThyX1 and/or ThyX2 with alpha-proteobacteria, the differences between the ML tree and these alternative topologies were significant (constraints 2 to 4, *P* << 0.001), rejecting the possibilities of ThyX1 and ThyX2 origins by EGT from mitochondria. We repeated the topology test that included *Spironema* in ThyX1, and it had no effect on the significance of the topology results. This result distinguishes between 2 proposed scenarios where ThyX in social amoebae was either from HGT or EGT (Stern et al. 2010). Our analyses using currently additional available genomic and metagenomic sequences now strongly support the HGT hypothesis.

### The Eukaryotic Species Included in the Dcd and ThyX Phylogenetic Trees

The sequences included in the above-described phylogenies are from BLASTp searches of Dcd1_Dicty_ and Dcd2_Dicty_ that identified numerous homologs from both metagenomics-derived and genome sequencing projects, with taxonomic classifications assigned to several lineages of bacteria, some archaea, and a few viruses. Among eukaryotes, through searches in the National Center for Biotechnology Information (NCBI) (ncbi.nlm.nih.gov), Dcd1 homologs were detected in all 4 groups of social amoebae: group 1 (*Cavenderia*), group 2 (*Acytostelium* and *Heterostelium*), group 3 (*Tieghemostelium*), and group 4 (*Dictyostelium* and *Polysphondylium*). In addition, Dcd1_Dicty_ homologs were detected in other eukaryotes including nonsocial Amoebozoa (*Planoprotostelium* and *Pelomyxa*), some metamonads (*Kipferlia* and *Histomonas*), the Discoba genus *Naegleria*, Obazoa fungi in Glomeromycetes (*Diversispora*, *Gigaspora*, *Paraglomus*, and *Rhizophagus*), and chytrids (*Gonapodya*). In subsequent BLASTp searches of the new EukProt database (Richter et al. 2022), we detected Dcd1 homologs from additional Amoebozoa species such as *Arcella intermedia*, *Vermamoeba vermiformis*, and *D. jomungandri* and from other eukaryotic groups (supplementary fig. S4, Supplementary Material online; Fig. 5). Detailed analyses of the eukaryotic clades in the ML trees (supplementary fig. S6, Supplementary Material online) show that the multiple NCBI-acquired sequences and some of those from EukProt form monophyletic clades within the same major eukaryotic groups. In Fig. 6, these are marked with black rectangles, while some unique sequences and sequences that do not form a monophyletic clade within the same major eukaryotic group are marked with gray rectangles.

For Dcd2_Dicty_, in NCBI, eukaryotic homologs were found in social amoebae and the cercozoan *Plasmodiophora brassicae*. Other potential eukaryotic protein matches appeared to be the result of contamination in the genome assembly from endosymbiont genomes (supplementary table S1, Supplementary Material online). In EukProt, Dcd2 homologs are also found in some other Amoebozoa species such as *Amoeba proteus*, *A. intermedia*, and *V. vermiformis* and in other eukaryotic groups including Rhodophyta, CRuMs, and Rhizaria (supplementary fig. S4, Supplementary Material online; Fig. 6). With the combined sequences from NCBI and EukProt, compared with *dcd1* homologs, *dcd2* homologs have a narrower distribution among eukaryotes, which suggests that *dcd1* is more ancient than *dcd2*. In Fig. 6, for Dcd2 as with Dcd1, the black rectangles indicate primarily annotated genome sequences (Amoebozoa and Rhizaria), while gray rectangles indicate single sequences and sequences from transcriptome studies that do not form a monophyletic clade within the same major eukaryotic group (CRuMs and Rhodophyta).

A BLASTp search using the *D. discoideum* ThyX identified close homologs from specific lineages of eukaryotes and bacteria, namely groups 1 to 4 of social amoebae and metagenomic bacterial samples from planctomycetes (Fig. 5). More eukaryotic homologs were also detected from some metamonad genome assemblies (*Histomonas* and *Tritrichomonas*). Other potential eukaryotic matches were excluded as possible contaminants because they are nearly identical to known bacterial endosymbionts (e.g. cyanobacteria) or located within a bacterial-like segment of eukaryotic genome assemblies (supplementary table S1, Supplementary Material online). One additional entry from EukProt is a sequence from *Spironema* sp. that was identified through transcriptomics (Fig. 6, supplementary fig. S6c, Supplementary Material online).

### Phylogenies of the Eukaryotic dCTP Deaminases and ThyX Clades and the Pattern of Homologous Introns Support the Ancient Acquisition of *dcd1*

**Fig. 7.**
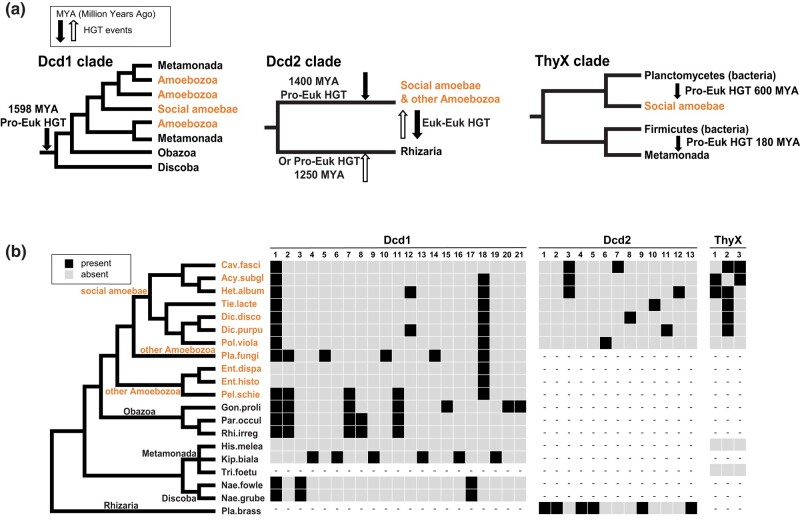
Simplified eukaryotic protein clades extracted from the ML trees each with the approximated time of the HGT event, plus the intron content of *dcd1*, *dcd2*, and *thyX*. a) The major eukaryotic clades containing Dcd1, Dcd2, ThyX1, and ThyX2 (from Fig. 5) with likely HGT timings. From left to right: Dcd1 entered the common ancestor of Amoebozoa, Obazoa, Metamonada, and Discoba. Dcd2 is gained by a transfer to either Amoebozoa or Rhizaria and then transferred to the other. ThyX1 entered social amoebae from Planctomycetes. ThyX2 entered Metamonads through a separate HGT from Firmicutes when both were parasites of a mammalian host (180 MYA is a rough estimate based on the suggested evolutionary emergence of mammals). b) Intron content of *dcd1*, *dcd2*, and *thyX* in social amoebae and other eukaryotes. A black box indicates the presence of an intron, a gray box indicates an absence, and dashes (–) indicate an absent gene. The cladogram represents the systematic relationships among sampled eukaryotic species (Brown et al. 2018; Burki et al. 2020; Schön et al. 2021; Strassert et al. 2021). Full names of species are shown in the trees in Fig. 5. Amoebozoa species that include social amoebae are in orange. The left panel shows introns in *dcd1* that are in social amoebae and other eukaryotes. The middle panel shows the introns in *dcd2* that are present in social amoebae and *Plasmodiophora*. The right panel shows introns in *thyX* that are in social amoebae. Genes of *Tritrichomonas* and *Histomonas* rarely have introns (Benchimol et al. 2017; Palmieri et al. 2021). The numbers above each panel indicate the relative positions of the introns from 5′ to 3′. The nucleotide positions of each intron are in supplementary fig. S8, Supplementary Material online which shows the exon alignment and intron sizes for each homologous gene.

Our phylogenetic analyses of the dCTP deaminase and ThyX protein clades provide an evolutionary context for the HGT or EGT events. In the case of Dcd1, the phylogeny of the protein clades within Amoebozoa, Obazoa, Discoba, and Metamonada largely agrees with the systematic relationships, except the Metamonada group together with Amoebozoa, rather than Discoba (Fig. 7a). A larger discrepancy with the systematic phylogeny is the individual and sporadic Dcd1 sequences in Glaucophyta, Cryptista, Haptista, Alveolata, and Hemimastix that nest within the Amoebozoa Dcd1 clade (supplementary fig. S6a, Supplementary Material online), rather than being outgroups. These, however, could indicate eukaryote–eukaryote HGT from Amoebozoa.

The intron analysis of the *dcd1* genes agrees with our protein phylogeny. Identification and location of introns was accomplished by aligning the amino acid sequences and then examining the exons (supplementary fig. S8, Supplementary Material online). Introns located at the same positions in diverse eukaryotic lineages are inferred to be homologous introns, while others can be unique in specific species. An intron is inferred to be older when it is shared among more diverse eukaryotic lineages. Intron insertions were shown to occur soon after a bacterial gene entered the genome of a fungal group ancestor (Da Lage et al. 2013), and this same phenomenon may be observed in *dcd* and *thyX* homologs (Fig. 7b). Introns shared among members of the *dcd1* clade indicate the acquisition of the gene in the common ancestor of Amoebozoa, Obazoa, and Discoba over 1,500 MYA (Fig. 7a and b) (Gueidan et al. 2011; Parfrey et al. 2011; Kumar et al. 2017). For example, i1 is shared among some Amoebozoa, fungi (Obazoa), and *Naegleria* (Discoba), while i2 and i7 are shared between some Amoebozoa and Obazoa, and i18 is shared by most Amoebozoa (Fig. 7b). The distribution of i1 in *dcd1* may have happened through 2 scenarios. The intron could have been acquired by a particular eukaryotic lineage (for example, amoebae), and then, the *dcd1* gene with i1 was subsequently transferred through eukaryote–eukaryote HGT events among the Amoebozoa, Obazoa, and Discoba groups. Alternatively, i1 could have been inserted quickly after *dcd1* was acquired by a eukaryotic common ancestor through HGT or EGT and then was lost from those lineages that have *dcd1* but lack the intron. Since all 3 closely related major eukaryotic groups with *dcd1* widely share i1 (Fig. 7b), the most likely scenario is an ancient HGT or EGT event of *dcd1* by a common ancestor of Amoebozoa, Obazoa, and Discoba.

For Dcd2, the strongest sequence evidence (black rectangles in Fig. 6) shows its presence in the Amoebozoa and Rhizaria groups that are not closely related, each forming their own monophyletic clade. If Dcd2 is gained through the common ancestor of these 2, multiple losses must account for its absence in many eukaryotic clades. A more plausible scenario would be an HGT event from a prokaryote or virus into the ancestor of Amoebozoa 1,400 MYA and then a transfer of *dcd2* from Amoebozoa to Rhizaria. Alternatively, *dcd2* was transferred from a prokaryote or virus to an ancestor of Rhizaria 1,250 MYA and then transferred from Rhizaria to some Amoebozoa (Fig. 7a) (Gueidan et al. 2011; Parfrey et al. 2011; Kumar et al. 2017). The 3 *dcd2* sequences in CRuMs and Rhodophyta nest within the Amoebozoa clade (supplementary fig. S6b, Supplementary Material online), which may indicate eukaryote–eukaryote HGT from Amoebozoa. For *dcd2*, the only homologous intron i3 is shared between groups 1 and 2 of social amoebae, and the rest of the introns are species-specific (Fig. 7b).

ThyX is present in the Amoebozoa and Metamonada groups. Our ThyX phylogenetic analysis, however, clearly shows ThyX1 (in social amoebae) and ThyX2 (in Metamonada) are from 2 separate prokaryotic to eukaryotic HGTs (Fig. 7a). ThyX1 entered the ancestor of social amoebae from Planctomycetes 600 MYA (Heidel et al. 2011), while ThyX2 was acquired by parasitic Metamonada from an animal parasitic Firmicutes bacterium 180 MYA when mammals emerged (Damas et al. 2022). For introns in *thyX*, i2 is shared among almost all groups of social amoebae, suggesting the entrance of ThyX1 in the common ancestor of social amoebae. The remaining introns are more narrowly shared among species within particular social amoebae lineages. The absence of introns from genes of *Histomonas* and *Tritrichomonas* (Benchimol et al. 2017; Palmieri et al. 2021) cannot contribute to the estimation for the HGT timing of ThyX2.

### GC Content and Codon Usage of Eukaryotic Dcd and ThyX Homologs

Anciently horizontally transferred genes are expected to have a %GC similar to that of their current hosts (Husnik and McCutcheon 2018), while recently horizontally transferred genes are expected to have a %GC similar to that of the donor genomes. The eukaryotic *dcd1*, *dcd2*, and *thyX* genes from NCBI were examined to see if they display an ancient or recent pattern of GC content. The %GC of all the *dcd1*, *dcd2*, and *thyX* eukaryotic homologs are slightly higher but generally track closely with the overall %GC of their eukaryotic (host) genomes. The %GC of the *dcd1* and *thyX* eukaryotic homologs is lower than that of their respective prokaryotic sister clades (potential donors), whereas the %GC of the eukaryotic *dcd2* overlaps with the %GC of their sister clade (potential donors) identified in our phylogenetic analyses (supplementary fig. S9 and supplementary table S2, Supplementary Material online). Assuming HGT is the source of the genes, these observations support ancient rather than recent HGTs.

The coding sequences of *dcd1_Dicty_*, *dcd2_Dicty_*, and *thyX_Dicty_* are 29%, 31.1%, and 33.8% GC, respectively, slightly higher than the average %GC of *D. discoideum* exons (Eichinger et al. 2005) but at the lower end of the 29.2% to 42.5% GC range of selected native *D. discoideum* coding gene sequences used for comparison (*tubA2*, 29.4%; *dut*, 32.8%; *dCMP deaminase 1*, 36.6%; *dCMP deaminase 2*, 35.9%; *dCMP deaminase 3*, 29.2%; *dCMP deaminase 4*, 30.7%; *act5*, 41.9%; *act8*, 42.5%). The similarity in %GC of the *dcd1*, *dcd2*, and *thyX* genes to the %GC of other genes of *D. discoideum* is consistent with their ancient acquisitions.

The AT richness of the *D. discoideum* genome has effects at the codon level, where codons ending in A and T are favored over G and C (Eichinger et al. 2005). Consistent with their adapted-to-host GC content, the coding sequences of *dcd1_Dicty_*, *dcd2_Dicty_*, and *thyX_Dicty_* use the most preferred codons in *D. discoideum* for each amino acid. Exceptions are for Thr where the second most frequent codon (ACT) was used in *dcd1_Dicty_* and *thyX_Dicty_*, and for histidine, CAT and CAC are used equally in *dcd2_Dicty_*. The minor deviations from the preferred codon usage of these 3 presumptive HGT genes are comparable with that seen in native genes included for comparison (supplementary table S3a, Supplementary Material online).

The analyses of GC content and codon usage are consistent with the phylogenetic analyses that indicate the relatively lengthy presence of at least hundreds of millions of years ago for *ThyX_Dicty_* and longer for *dcd2_Dicty_* and *dcd1_Dicty_* (Fig. 7a), which provided time for the GC content and codon usage to acclimate to the host genome.

## Discussion

### Dcd1_Dicty_ and Dcd2_Dicty_ Are Functional dCTP Deaminases in Both Prokaryotes and Eukaryotes

Our complementation studies demonstrate that expression of Dcd1_Dicty_ and Dcd2_Dicty_ each fully rescues the slow growth phenotype of the dCTP deaminase knockout of *E. coli* and supports the suggestion that the *D. discoideum* dCTP deaminase genes are functional in this prokaryote (Fig. 2). These findings indicate that the predicted *D. discoideum* dCTP deaminases can contribute to the biosynthesis of dTTP. Although the *E. coli* genome and its *dcd* are 50.8% and 60.3% GC, respectively (Riley et al. 2006), the lower %GC of *dcd1_Dicty_* and *dcd2_Dicty_* (supplementary table S2, Supplementary Material online) and the differences in codon usage of the *D. discoideum* and *E. coli* genes (supplementary table S3b, Supplementary Material online) do not appear to hinder the ability of the *D. discoideum* genes to complement the knockout. In experiments where growth started in chilled media, all examined strains show hindered growth. The Δ*dcd E. coli* + *dcd1_Dicty_* grew nearly as well as WT + EV, while the Δ*dcd E. coli* + *dcd2_Dicty_* transformant grew even more poorly than the Δ*dcd E. coli* + EV. This may indicate some functional differences between Dcd1_Dicty_ and Dcd2_Dicty_ in nonideal growth conditions (supplementary fig. S10, Supplementary Material online).

Dcd1_Dicty_ and Dcd2_Dicty_ have different impacts on the dCMP deaminase knockout of *S. pombe* (Δ*S. pombe*) (Fig. 3). Dcd2_Dicty_ is more successful than Dcd1_Dicty_ in improving the growth of the knockout both in the absence and presence of sublethal amounts of HU, a specific inhibitor of ribonucleotide reductase. In contrast, *dcd1_Dicty_* transformants, in the absence of HU, do not significantly aid growth, but in the presence of HU, Dcd1_Dicty_ modestly tempers the severity of the inhibitor. The limited ability of *dcd1_Dicty_* and *dcd2_Dicty_* to rescue Δ*S. pombe*, unlike their full rescue of the Δ*dcd E. coli*, may be due to several factors. These include codon usage (supplementary table S3c, Supplementary Material online) and %GC differences between the coding genes of the *D. discoideum* dCTP deaminases (*dcd1_Dicty_* and *dcd2_Dicty_* are 29% and 31.1% GC, respectively) and *S. pombe* dCMP deaminase (41.4% GC; Harris et al. 2022). Another factor may be the absence of introns in the expression plasmids, which has been argued to reduce the efficiency of transcription and translation in eukaryotes (Le Hir et al. 2003). Metabolic compatibility also may be an issue. For example, the synthesis of dTTP relies largely on an active dCMP deaminase because *S. pombe* lacks a dTTP salvage pathway (Grivell and Jackson 1968). With the presence of the *S. pombe* dUTPase (Kanehisa and Goto 2000), the introduction of *D. discoideum* dCTP deaminases into Δ*S. pombe* should construct a different pathway for dTTP synthesis. However, if the separately expressed Dcd1_Dicty_ or Dcd2_Dicty_ are less active in *S. pombe* than in *E. coli* or work less efficiently in the pathway, then the *dcd1_Dicty_* or *dcd2_Dicty_* will incompletely restore the Δ*S. pombe* to WT growth.

### ThyX_Dicty_ Is a Functional TS in Prokaryotes

In *D. discoideum*, the single copy of *thyX_Dicty_* is essential, and its deletion creates thymidine-requiring cells. The return of *thyX_Dicty_* restores the WT phenotype indicating the gene codes for a functional TS in *D. discoideum* (Dynes and Firtel 1989). In this study, the rescue of Δ*thyA E. coli* with *thyX_Dicty_* shows that ThyX_Dicty_ is functional also in prokaryotes (Fig. 4).

The ThyX from *Paramecium bursaria* Chlorella virus 1 (PBCV-1) has been characterized biochemically (Graziani et al. 2004) and considered the most catalytically active ThyX studied (Escartin et al. 2008). When it replaced the chromosomal *E. coli thyA*, it failed to rescue the long filamentous phenotype of the mutants (Escartin et al. 2008). In contrast, the *D. discoideum* ThyX_Dicty_ expressed in Δ*thyA E. coli* restores the typical rod shape of WT cells (Fig. 4d). ThyX_Dicty_ appears to be functionally more compatible with *E. coli* than the PBCV-1 ThyX, although the expression levels of the heterologous genes may differ since they are introduced differently (chromosomally or through a plasmid). Other attempts have been made to rescue Δ*thyA E. coli* mutants: ThyX from *Borrelia hermsii* partially rescued, while *B. burgdorferi* thyX failed to rescue (Zhong et al. 2006). Since the *Borrelia* proteins were expressed with N-terminal His tags rather than at the C-terminus as done with ThyX_Dicty,_ the position of the tag may have influenced the activity of the enzyme and could have affected the efficiency of rescue. Additional biochemical analyses of ThyX_Dicty_ are needed to establish its catalytic properties and to compare it with other experimentally characterized ThyX.

### Dcd1 and Dcd2 Have Different Origins and Dcd1 Is More Ancient Than Dcd2

Phylogenetic analyses of the eukaryotic Dcd proteins indicate that Dcd1 and Dcd2 are distinct paralogs and did not arise from a eukaryotic-specific gene duplication. Instead of being sister groups to each other, they each group to different sets of prokaryotes with strong bootstrap support (Fig. 5a). Moreover, topology tests strongly rejected the hypotheses forcing Dcd1 and Dcd2 to be sister groups (constraints 1 and 4; Fig. 5b). The expression patterns of *dcd1_Dicty_* and *dcd2_Dicty_* are different (supplementary fig. S1, Supplementary Material online), which may indicate distinct roles of the proteins during growth and development of social amoeba.

**Fig. 8.**
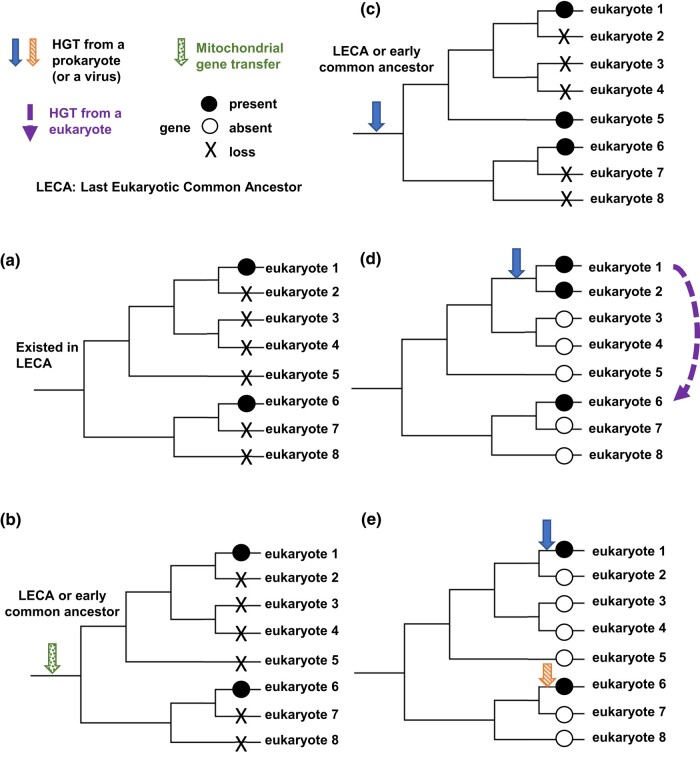
Proposed scenarios for the distribution of *dcd1*, *dcd2*, and *thyX* in eukaryotes. a) The gene is present in the LECA, and then, it is lost by multiple species, and only eukaryotes 1 and 6 retain the gene. b) The gene is gained through a transfer from a mitochondrion, and then, it is lost by multiple species, and only eukaryotes 1 and 6 retain the gene. c) The gene entered a common ancestor of some eukaryotes through HGT and is lost by multiple members of that clade, so only eukaryotes 1, 5, and 6 retain the gene. d) The gene entered a common ancestor of some eukaryotes through HGT, and a second HGT occurred from eukaryote 1 to eukaryote 6. e) Two separate HGTs introduced the gene into eukaryotes 1 and 6.

The monophyly of the diverse eukaryotes in the Dcd1 clade (Fig. 5a) suggests 5 scenarios in how these genes became distributed. One scenario posits the origin of Dcd1 in the last eukaryotic common ancestor (LECA), implying that many eukaryotic lineages lost *dcd1* with some species retaining *dcd1* (Fig. 8a). If *dcd1* existed in the LECA, then the *dcd1* eukaryotic clade should form a monophyletic group outside all bacterial Dcd clades (Lester et al. 2006). However, the ML tree shows Dcd1 clearly nested within a larger bacterial clade (Fig. 5a). A second scenario specifies that *dcd1* was acquired from the ancient mitochondrion to LECA or to an early eukaryotic ancestor via EGT (Fig. 8b). Although the ML tree does not group *dcd1* with alpha-proteobacteria, this scenario remains possible because our topology test does not reject it. Two scenarios stipulate an ancient HGT of *dcd1* from a prokaryote into an early eukaryotic ancestor, followed either by recurrent losses of the gene from the lineages that no longer have *dcd1* (Fig. 8c) or by multiple HGT events among the distinct eukaryotic lineages that presently have *dcd1* (Fig. 8d). Our data indicate that *dcd1* was initially acquired by the common ancestor of Amoebozoa, Obazoa, Metamonada, and Discoba. This conclusion is supported by the fact that most sequences in the Dcd1 eukaryotic clade are from these groups and their protein phylogeny aligns with established systematic relationships. Further supporting the idea of subsequent eukaryotic–eukaryotic HGTs from Amoebozoa (Fig. 8d), the remaining Dcd1 sequences found in Glaucophyta, Cryptista, Haptista, Alveolata, and Hemimastix all nested within the Amoebozoa clade. In contrast and less likely, scenario c would require Glaucophyta, Cryptista, Haptista, Alveolata, and Hemimastix to form clades outside the Amoebozoa, Obazoa, Metamonada, and Discoba monophyletic clades, which is not supported by our data. A fifth scenario specifies that the eukaryotic groups acquired *dcd1* genes through separate HGTs from different prokaryotes (Fig. 8e), which is unlikely because all eukaryotic Dcd1 homologs form a monophyletic clade. Overall, although scenarios b and c are plausible, our phylogenetic analyses, topology tests, and the widespread but irregular distribution of *dcd1* among eukaryotes provide the most support for the scenario involving an HGT from a prokaryote to an ancient eukaryotic ancestor, followed by multiple eukaryotic HGTs into protists (scenario d).

The narrower distribution of *dcd2* homologs among eukaryotes and the limited sharing of introns among homologous genes (Figs. 5a and 7b) indicate that *dcd2* was also an ancient acquisition but more recent than *dcd1* and argue against the possibility that *dcd2* was present in the LECA (Fig. 8a). Our topology test rejected the EGT acquisition of *dcd2* (Fig. 8b). Further, since all eukaryotic Dcd2 homologs form a monophyletic clade, different origins of Dcd2 are unsupported (Fig. 8e). Our phylogenetic analyses and topology tests show that *dcd2* likely was acquired via an initial HGT from prokaryotes or viruses. Two different paths can explain the limited distribution of *dcd2* among 4 eukaryotic groups. One is that after HGT into the common ancestor of Amoebozoa and Rhizaria, multiple eukaryotic clades lost *dcd2* (Fig. 8c). The second is that after the initial HGT to 1 eukaryotic group, eukaryote-to-eukaryote gene transfers delivered *dcd2* among the 2 to 4 eukaryotic groups (Fig. 8d). Though both scenarios are possible, scenario c requires the extensive loss of *dcd2* from numerous clades, while scenario d is a more parsimonious explanation for the restricted distribution of *dcd2*.

### Separate HGT Events Are Responsible for ThyX in a Small Number of Eukaryotes

ThyX in eukaryotes is limited to 2 distantly related eukaryotic clades (Fig. 5c; 6). One clade includes Amoebozoa and *Spironema* (ThyX1), and a second includes metamonads (ThyX2). ThyX1 and ThyX2 have narrow distributions, which argue against the presence of ThyX in the LECA (Fig. 8a). Topology tests rejected both the acquisition of ThyX1 and ThyX2 by EGT (Fig. 8b) and the possibility that the eukaryotic ThyX1 and ThyX2 clades are closely related (Fig. 8c and d). Thus, the independent acquisition of ThyX by amoebae and metamonads from different prokaryotes is consistent with and supported by the phylogenetic analyses and topology tests (Fig. 8e). Since *thyA* is absent from social amoebae and the *Histomonads* and *Tritricomonads* belonging to metamonads, the acquired *thyX* presumably replaced *thyA* in these eukaryotic genomes.

### Analysis Using Alpha-Proteobacteria for the Possibility of EGT

Although our topology test rejected the EGT acquisition of *dcd2* or *thyX* (Fig. 8b), it remains formally possible that the present-day sequences of Rickettsiales or alpha-proteobacteria are not representative of the ancestral mitochondrial genes, as they may have since horizontally acquired a different *dcd2* or *thyX* gene (Nagies et al. 2020; Tria et al. 2021). We also note that an EGT from mitochondria and an HGT from alpha-proteobacteria cannot be distinguished with current methods, since they create identical patterns. From the dCTP deaminase tree, the alpha-proteobacteria clade is not closely related to the beta or gamma-proteobacteria clades. Thus, the current alpha-proteobacteria may or may not retain the Dcd of the ancestral mitochondria. For TS, alpha-proteobacteria shows 2 discrete groups having either ThyA or ThyX with neither known as the ancestral state (Stern et al. 2010). If ThyA is the ancestral state, then ThyX_Dicty_ cannot be an EGT. If ThyX is the ancestral state, and since the alpha-proteobacterial ThyX clade is not next to the ThyX of other proteobacteria, we cannot tell if the alpha-proteobacterial ThyX represents the ancestral mitochondrial ThyX. We then asked if the Dcd2 or ThyX eukaryotic clades are located near other proteobacteria besides alpha-proteobacteria, but they are not sister groups with any proteobacteria, which argues against the mitochondrial origin of these eukaryotic proteins (supplementary fig. S5, Supplementary Material online).

### Shared Environments May Have Facilitated the HGT of *dcd1*, *dcd2*, and *thyX*

If HGT is the major source of *dcd1*, *dcd2*, and *thyX1* in eukaryotes, then for its occurrence, we expect these eukaryotes to have shared the same environments with the putative prokaryotic and/or eukaryotic donors. The eukaryotes that have Dcd1, Dcd2, or ThyX1, except for some animal parasites (*Entamoeba*), are found in soil (social amoebae), soil-associated environments including plant roots (*Gigaspora*, *Rhizophagus*, *Plasmodiophora*), decayed plant matter (*Planoprotostelium*), and water or water sediments.

The potential prokaryotic donors for Dcd1 include a variety of groups such as Deltaproteobacteria, Chloroflexi, and Cyanobacteria. Most of these gene sequences were derived from metagenome samples collected from soil (Bell et al. 2020) or soil–water interfaces, including freshwater (Martins et al. 2018), ocean (Rasigraf et al. 2020), and hydrothermal sediments (Dombrowski et al. 2017; Zhou et al. 2020). The environmental locations of LECA eukaryogenesis and the common ancestor of Amoebozoa, Obazoa, Metamonada, and Discoba remain unknown. Asgard archaea, the archaeal ancestors of eukaryotes, are typically associated with water environments (Liu et al. 2021). Since the location of the ancestor is unknown, the environmental context for the initial HGT of *dcd1* from prokaryotes remains unclear. For the postulated subsequent eukaryotic–eukaryotic HGTs, they occurred most likely from the crust of earth or earth–water interfaces, where most protists with *dcd1* identified in this study are located.

Prokaryotic relatives of the eukaryotic Dcd2 clade are primarily from the Candidate Phyla Radiation and Chloroflexi; these metagenomic samples were extracted predominantly from water sediments (Anantharaman et al. 2016; Probst et al. 2018; Tully et al. 2018) or geothermally heated biofilms (Wang et al. 2019). Likewise, large nucleocytoplasmic DNA viruses (NCLDV) that possess Dcd2 homologs are found in soil (Schulz et al. 2018) or water sediments (Bäckström et al. 2019). The bacterial relatives of the social amoebae ThyX1 clade are in phylum Planctomycetota, which are found in soil crusts (Meier et al. 2021), fresh water (Zeng et al. 2020), and hydrothermal environments (Zhou et al. 2020). We hypothesize that within these shared earth's crust environments or land–water interfaces, ancestors of Amoebozoa or Rhizaria encountered prokaryotic or viral species possessing Dcd2, and the ancestor of social amoebae encountered prokaryotes with ThyX1 homologs, thus providing opportunities for HGT. On the other hand, ThyX2 eukaryotic homologs are found in metamonads that are animal parasites (*Histomonas* and *Tritrichomonas*), and it is not surprising to find that their closest prokaryotic clade includes a group of Clostridia. Both metamonads and Clostridia species are anaerobic lineages, and they are often animal symbionts/parasites (e.g. *Clostridium*, *Intestinimonas*, *Peptoclostridium*, *Ruminococcus*), suggesting that their shared environment may have facilitated this HGT event. We conclude that the ThyX2 entered Metamonads when mammals emerged ∼180 MYA. This is also supported by the finding that not all metamonads have ThyX2. For example, the Metamonada Anaeramoeba has ThyA, which indicates that ThyX2 is not in the common ancestor of all Metamonads but only in the animal parasite branch.

Studies have suggested that amoebae have exchanged genes with parasites and other microorganisms in their shared environments (La Scola et al. 2003; Moreira and Brochier-Armanet 2008; Moliner et al. 2010). For example, 25 genes are shared between *Dictyostelium* and the distantly related Discoba *Naegleria* (Andersson 2011). Within the *D. discoideum* genome, 18 genes were identified to be derived from HGT, including ThyX_Dicty_ (Eichinger et al. 2005). The dCTP deaminases studied here were not among the 18 genes, presumably because Eichinger et al. searched for bacteria-specific protein families in the *D. discoideum* genome, but the *dcd1_Dicty_* and *dcd2_Dicty_* dCTP deaminases belong to the dUTPase superfamily that is present in both prokaryotes and eukaryotes. This may also explain why a gene coding for a ribonucleotide reductase, another enzyme in the dNTP biosynthesis pathway, was suggested to be an HGT event (Crona et al. 2013) but was not included in the 18 HGT genes identified by Eichinger et al.

### The Archaeal and Viral Sequences

Archaeal Dcd sequences are distributed widely and nested within bacterial clades, which indicates HGT between bacteria and archaea (Fig. 5a; supplementary fig. S12, Supplementary Material online). Moreover, the archaeal Dcd sequences do not form a single monophyletic clade that is a sister group with the eukaryotic Dcd, which indicates that Dcd did not exist in the LECA. Viruses are known as agents that change eukaryotic genomes (Kieft and Anantharaman 2022). While the phylogenetic analyses show a possible viral origin for Dcd2, they show no support for a viral origin of Dcd1 with the currently available viral sequences. Metagenomics and next-generation sequencing are providing more viral sequences which might change the understanding of the phylogeny Dcd1 if as yet unsequenced viruses are responsible for gene transfers between prokaryotes and eukaryotes.

### dCTP Deaminase and dCMP Deaminase Are Not Mutually Exclusive and Their Presence in AT-Rich Genomes

The presence of dCTP deaminase is limited to a subset of unicellular eukaryotes, many of which have retained their dCMP deaminase, suggesting the 2 enzymes can coexist. When using the 4 annotated *D. discoideum* dCMP deaminases as queries, homologs for the 4 are shown to be present in all social amoebae. Using the *S. cerevisiae* dCMP deaminase as a query, homologs are found in Obazoa soil fungi that have Dcd1 homologs (supplementary table S2, Supplementary Material online). The BLASTp *e*-values (all smaller than 2e^−45^) provide strong evidence for the presence of both deaminases in these species. Using these same queries, larger *e*-values are found for presumptive homologs in Naegleria (from Discoba, 5e^−11^), Histomonas (in Metamonada, 6e^−14^), and Plasmodiophora (in Rhizaria, 2e^−7^), which lend modest support to the dual presence of the deaminases. Why would an organism use 2 pathways to produce dUMP? Social amoebae can grow as single cells and also undergo starvation-induced aggregation to form multicellular structures. We speculate the pathways are vital to the specific metabolic demands of the different lifestyles. Transcriptome analyses of the *D. discoideum* genes support this suggestion (supplementary fig. S1, Supplementary Material online). Why do other single-cell eukaryotes have the 2 pathways? We note that among the 19 species with Dcd1 or Dcd2, 16 of them also have dCMP deaminase homologs and most have AT-rich genomes (12 with genomes <40% GC and 2 genomes <50% GC) (supplementary table S2, Supplementary Material online). The presence of both pathways may contribute to AT-rich genomes but cannot be the only factor, since there are exceptions to this correlation (the genomes of both *Gonapodya prolifera* and *P. brassicae* are over 50%GC).

A related question is why social amoebae have multiple genes of both dCTP deaminase and dCMP deaminase. Social amoebae are unique because they have both Dcd1 and Dcd2. The only other eukaryote which may have both is *Diphylleia rotans*, a CRuMs species (Fig. 6). In this study, we did not find any specific bacteria with both Dcd1 and Dcd2. Genomes do not retain redundant genes without selection pressure (Conrad and Antonarakis 2007). Besides the above-stated possibility of expression in different life stages, we propose multiple copies of dCTP and dCMP deaminases are a benefit to social amoebae, because they provide precursors of dTTP required by the AT-rich (nuclear and mitochondria) genomes. Retention of multiple genes involved in dTTP biosynthesis is thus advantageous. Alternatively, the acquisitions of *dcd1_Dicty_* and *dcd2_Dicty_*, along with the multiple genes of dCMP deaminase, could make dTTP more abundant, altering the differential cost and availability of dNTPs. Due to its essential role as an energy source (Bentley and Parkhill 2004), ATP is abundant which makes dATP less costly, while it is energetically more expensive to synthesize GTP and CTP (Rocha and Danchin 2002). AT-rich genomes are thus an economical outcome. In either case, extra versions for both enzymes provide more precursors of dTTP that may have facilitated the AT-rich genomes in most social amoebae today.

### The Exchange of ThyX for ThyA in Social Amoebae Is Mysterious

In all eukaryotes, ThyA and ThyX are mutually exclusive, unlike dCTP deaminase and dCMP deaminase which can coexist, as indicated by our analyses. In this study, we did not identify any eukaryotes that have both *thyA* and *thyX*. In bacteria, *thyA* and *thyX* also are mutually exclusive, except for *Mycobacteria*, which have both, and are probably in the process of selecting one over another (Fivian-Hughes et al. 2012). The HGT of *thyX* and the reason for its replacement of *thyA* in social amoebae is currently unclear. It is possible that the substitution of *thyX* for *thyA* is effectively neutral, providing no adaptive advantage. In essence, once the *thyX* gene was acquired, it may have been an evolutionary coin flip as to which gene (*thyX* or *thyA*) was retained. It is also possible that ThyX_Dicty_ is a more efficient TS in social amoebae, so there is selective pressure to favor its retention. In vivo cofactors unique to ThyX, such as the levels of NADPH or the levels of reduced folate derivatives, may have favored the substitution of ThyX for ThyA in the ancestor of social amoebae. Since *thyA* is already lost from social amoebae genomes, we cannot compare the activities of ThyA_Dicty_ and ThyX_Dicty_. In future studies, ThyA from related Amoebozoa can be compared with ThyX_Dicty_ in vitro to evaluate their catalytic properties and in vivo to test their metabolic efficiencies.

## Supplementary Material

msad268_Supplementary_Data

## Data Availability

All data are incorporated into the article and its online Supplementary material.
