# Supplementary material for: Functional Prokaryotic-Like Deoxycytidine Triphosphate Deaminases and Thymidylate Synthase in Eukaryotic Social Amoebae: Vertical, Endosymbiotic, or Horizontal Gene Transfer?
Source: Mol Biol Evol. 2023 Dec 8;40(12):msad268. doi: 10.1093/molbev/msad268 (PMC10733785; doi:10.1093/molbev/msad268)
Supplement: msad268_Supplementary_Data [file msad268_supplementary_data.zip › Supplementary Figures and legends MBE final.pdf]

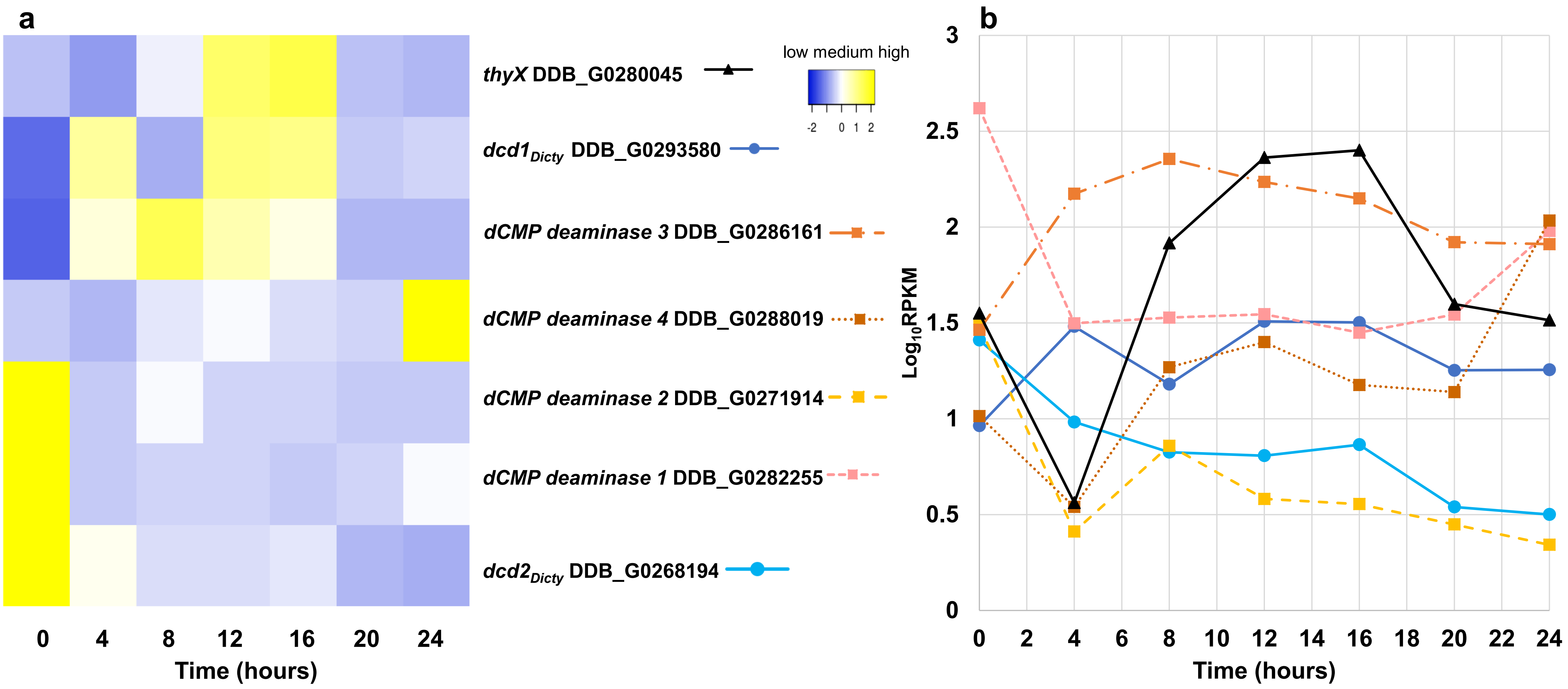

Supplementary Figure 1

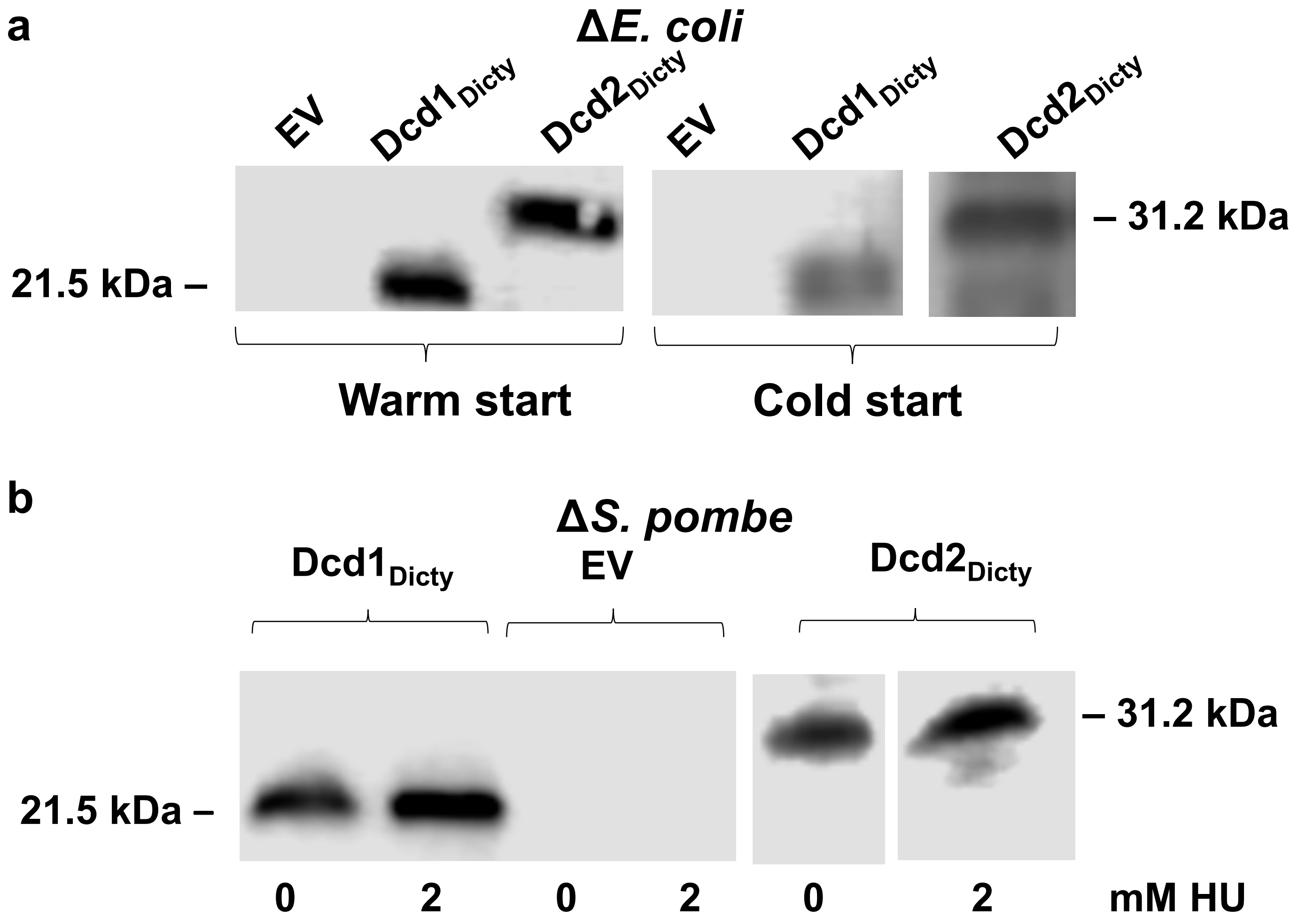

Supplementary Figure 2

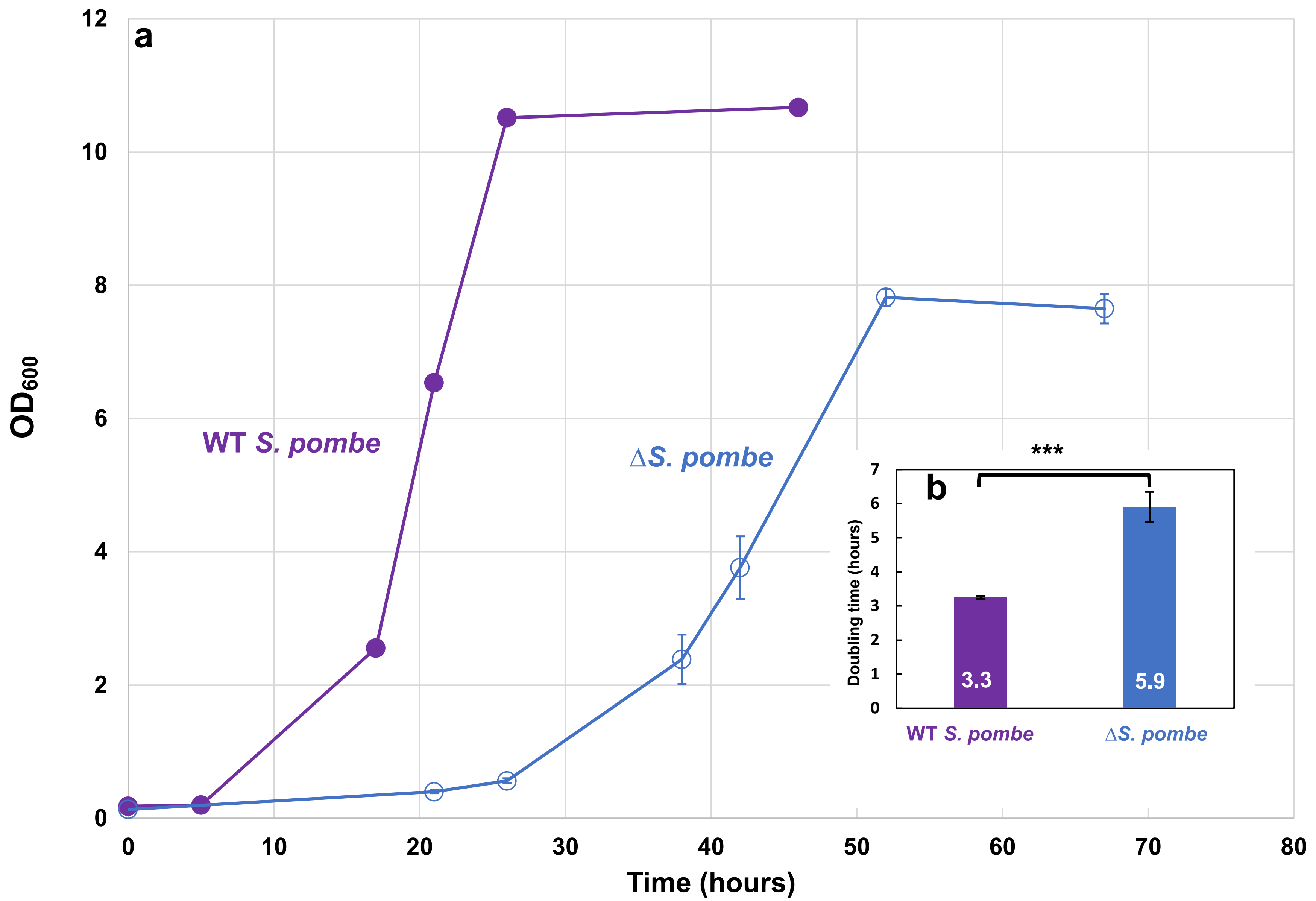

Supplementary Figure 3

Supplementary Figure 4a

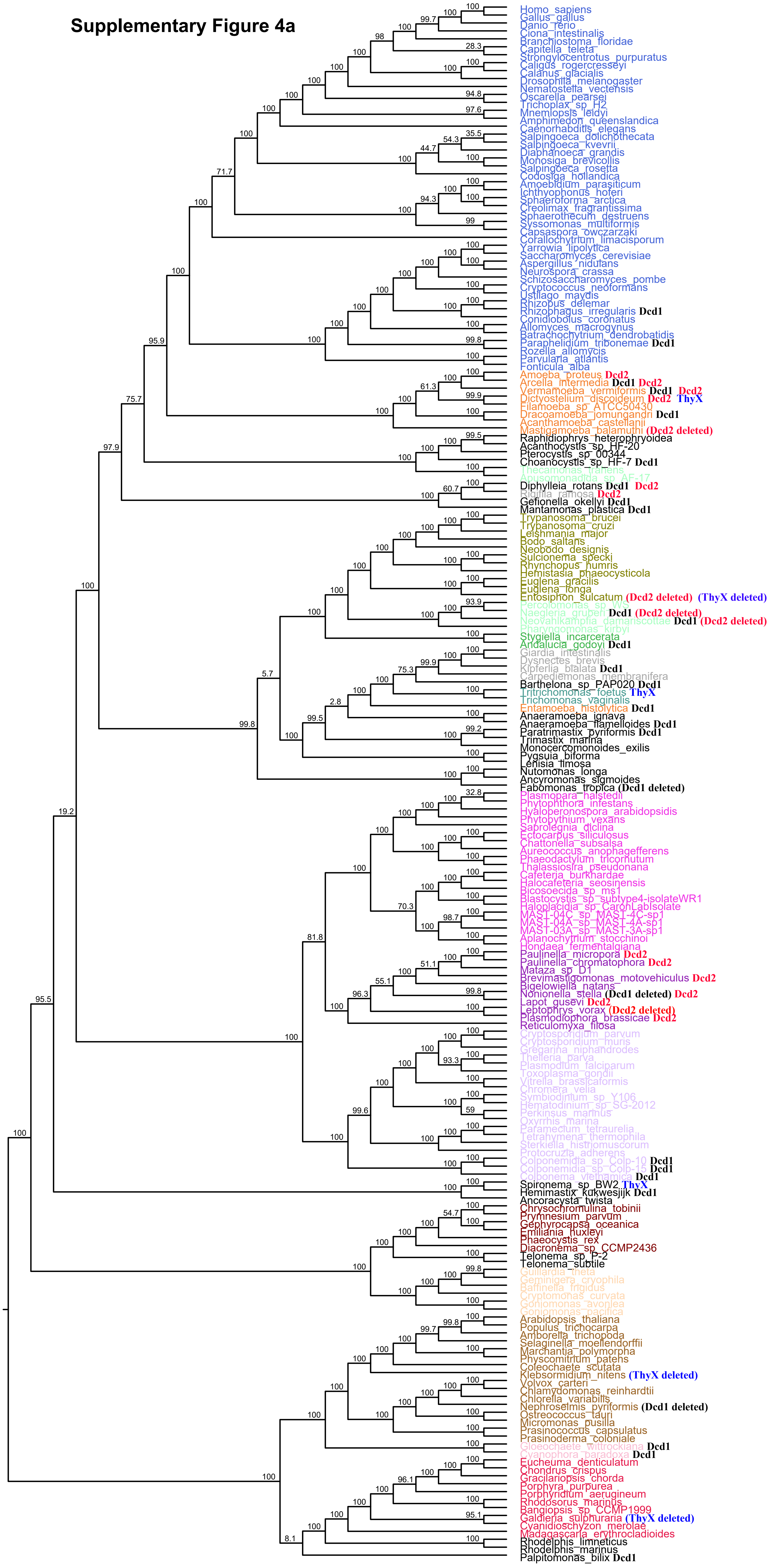

Figure 4b

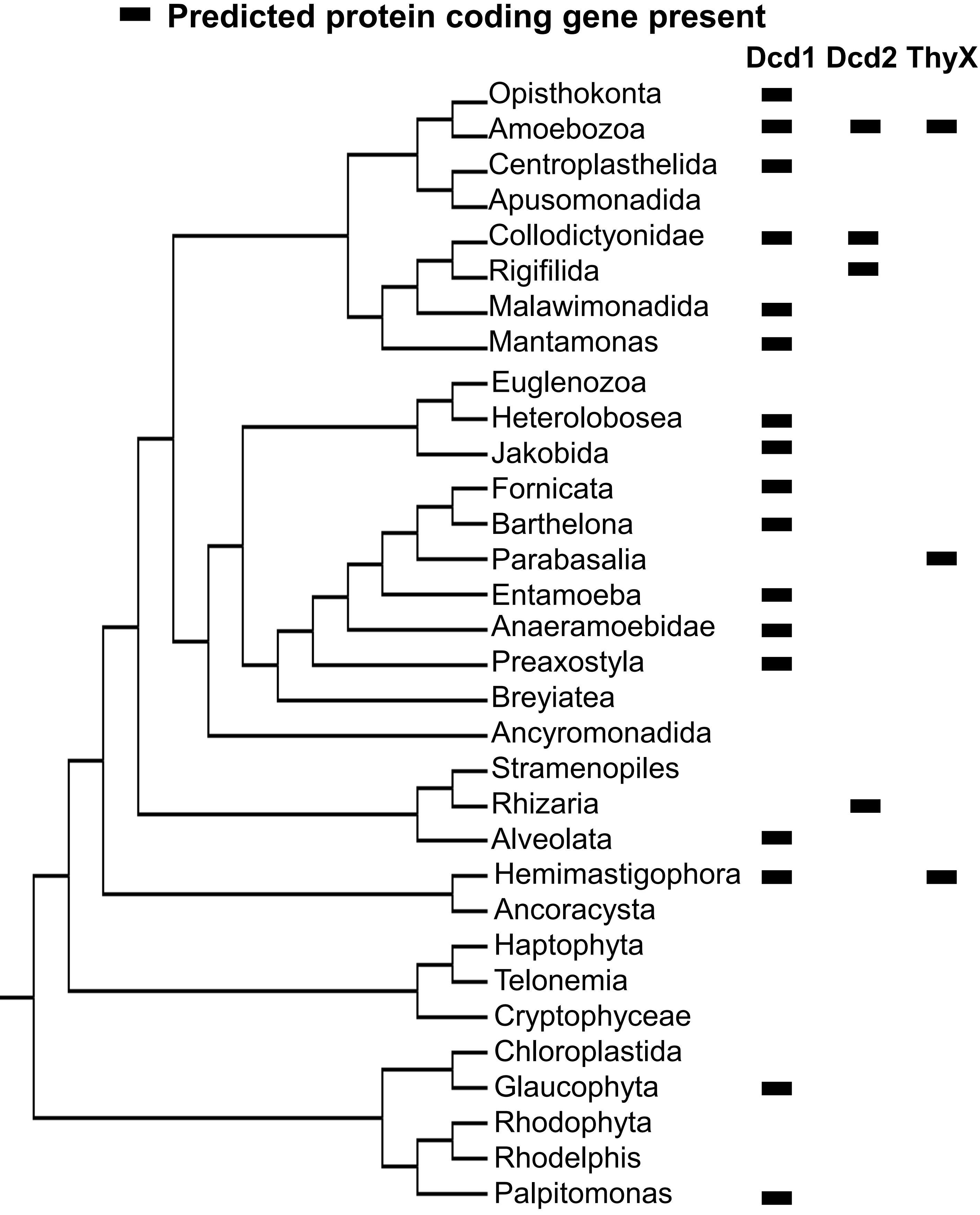

Predicted protein coding gene present
Dcd1 Dcd2 ThyX
Opisthokonta
Amoebozoa
Centroplasthelida
Apusomonadida
Collodictyonidae
Rigifilida
Malawimonadida
Mantamonas
Euglenozoa
Heterolobosea
Jakobida
Fornicata
Barthelona
Parabasalia
Entamoeba
Anaeramoebidae
Preaxostyla
Breyiatea
Ancyromonadida
Stramenopiles
Rhizaria
Alveolata
Hemimastigophora
Ancoracysta
Haptophyta
Telonemia
Cryptophyceae
Chloroplastida
Glaucophyta
Rhodophyta
Rhodelphis
Palpitomonas

Supplementary Figure 5

a

Dcd maximum likelihood phylogeny (235 species)

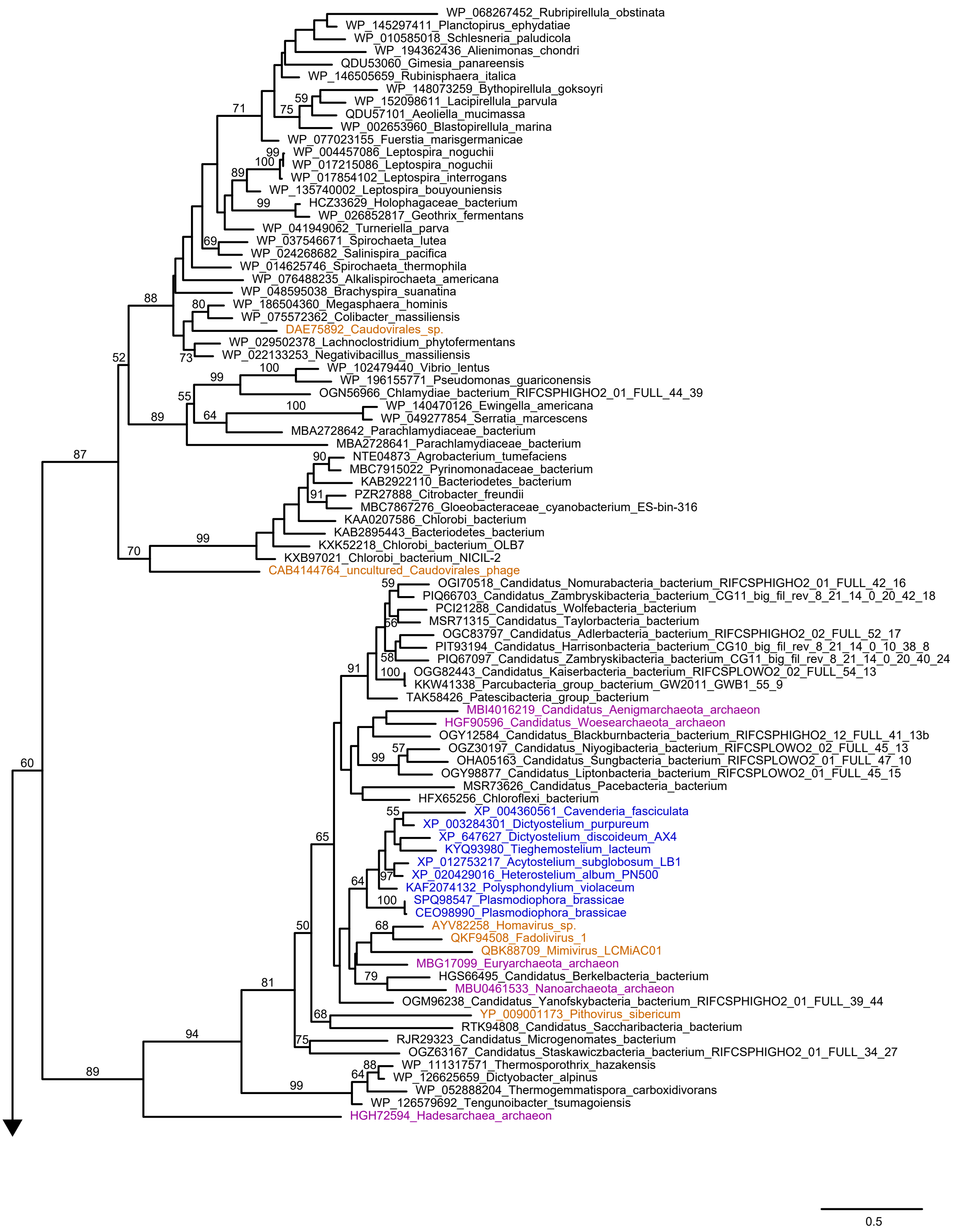

Supplementary Figure 5

b

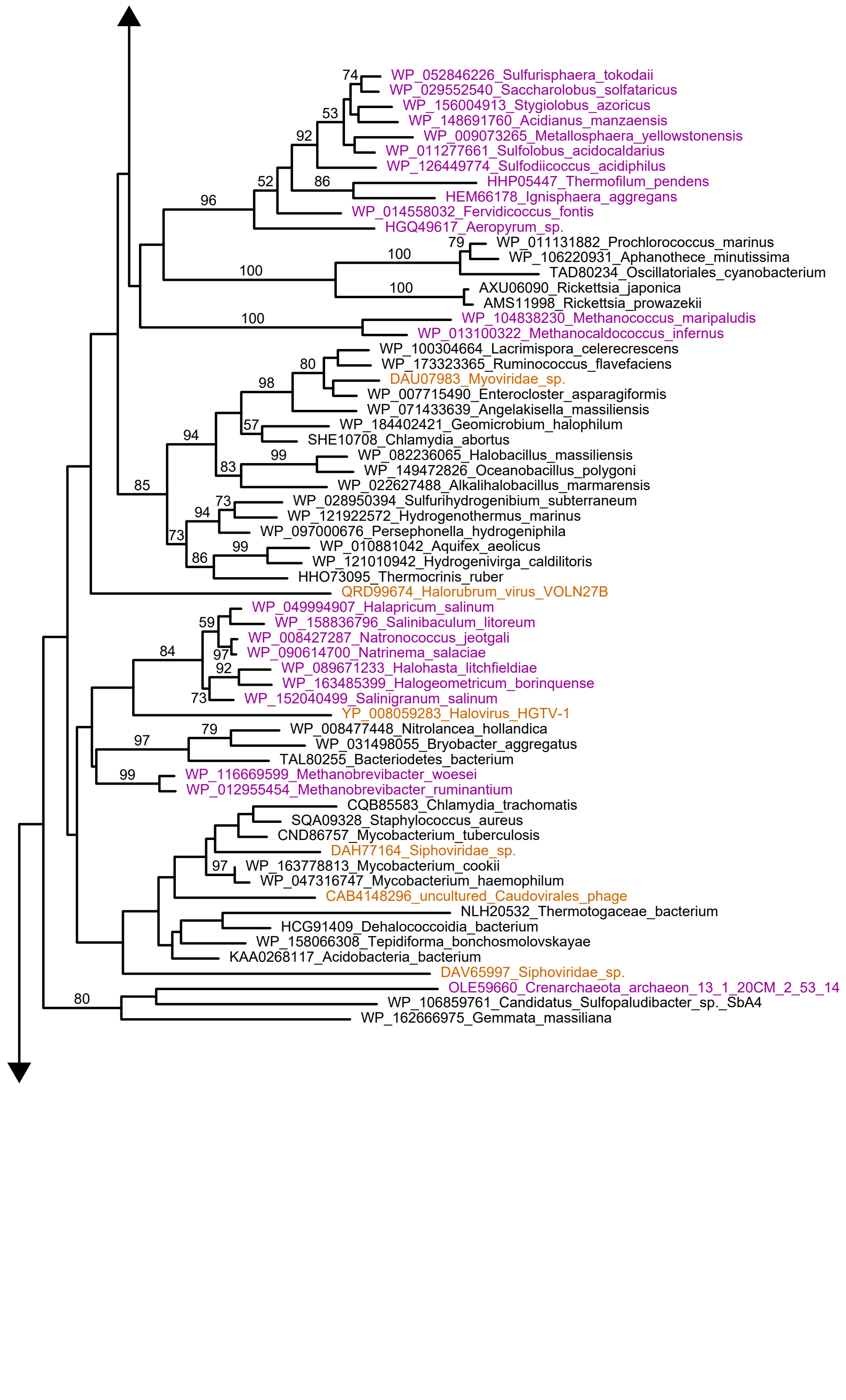

Supplementary Figure 5

c

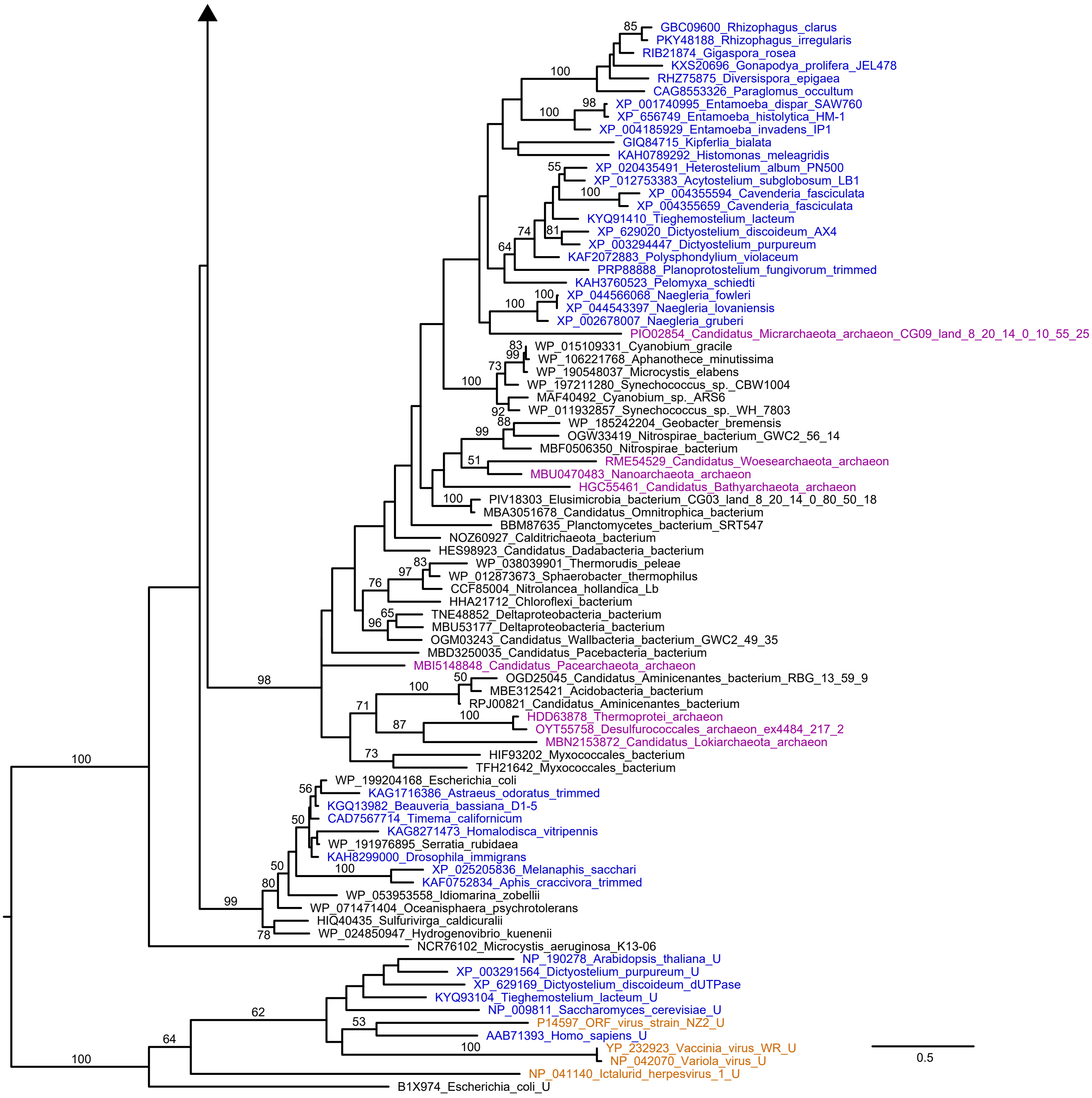

Supplementary Figure 6 a

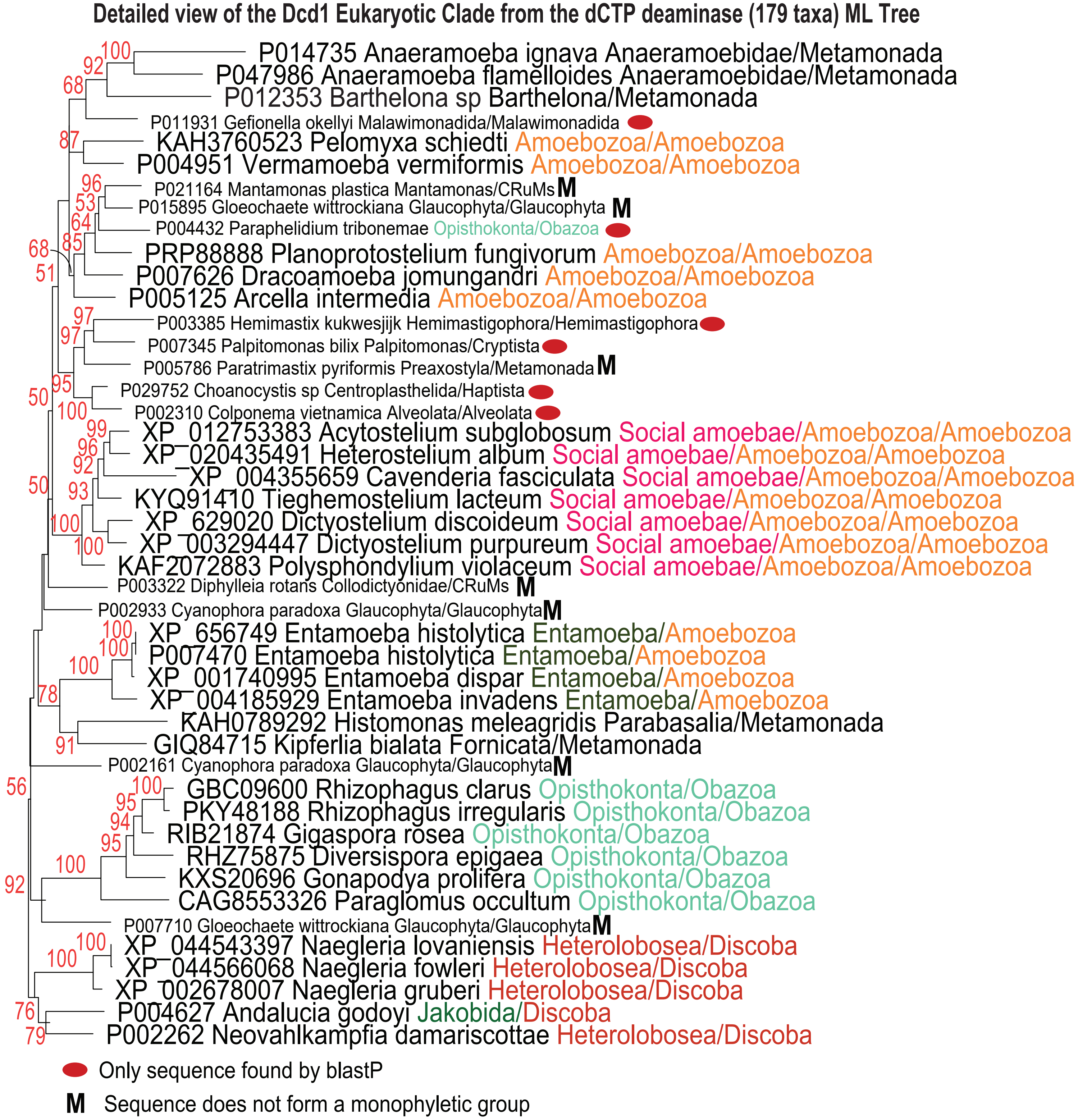

Supplementary Figure 6 b

Detailed view of the Dcd2 Eukaryotic Clade from the dCTP deaminase (179 taxa) ML Tree

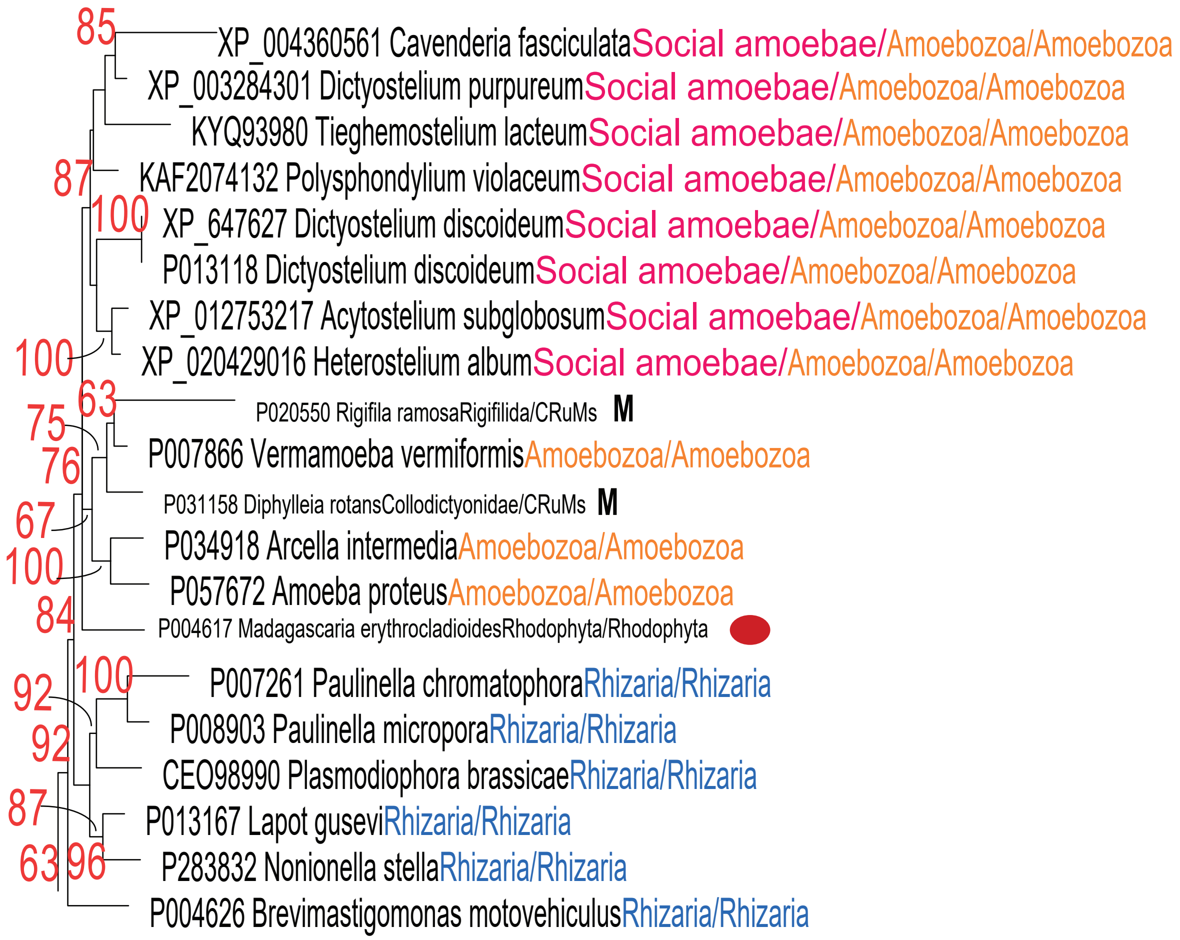

Supplementary Figure 6 c

Detailed view of the ThyX1 Eukaryotic Clade from the ThyX (116 taxa) ML Tree

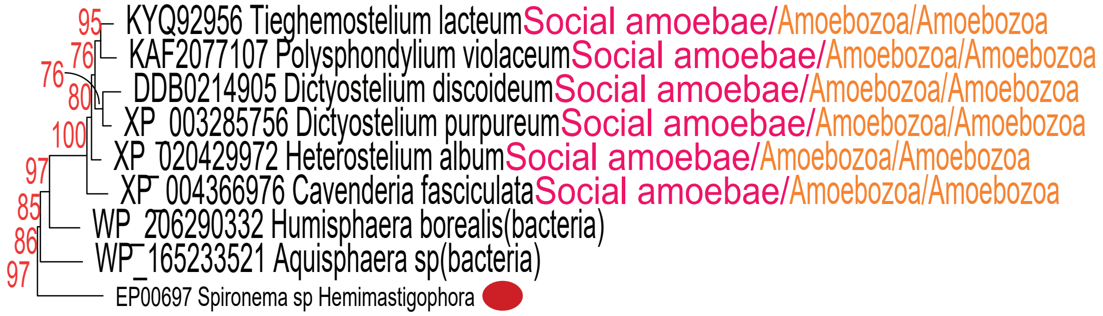

Detailed view of the ThyX2 Eukaryotic Clade from the ThyX (116 taxa) ML Tree

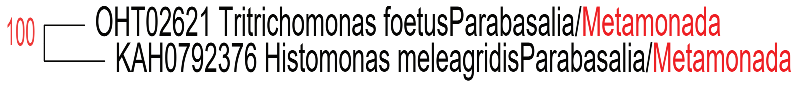

● Only sequence found by blastP

M Sequence does not form a monophyletic group

Supplementary Figure 7

a

ThyX maximum likelihood phylogeny (174 species)

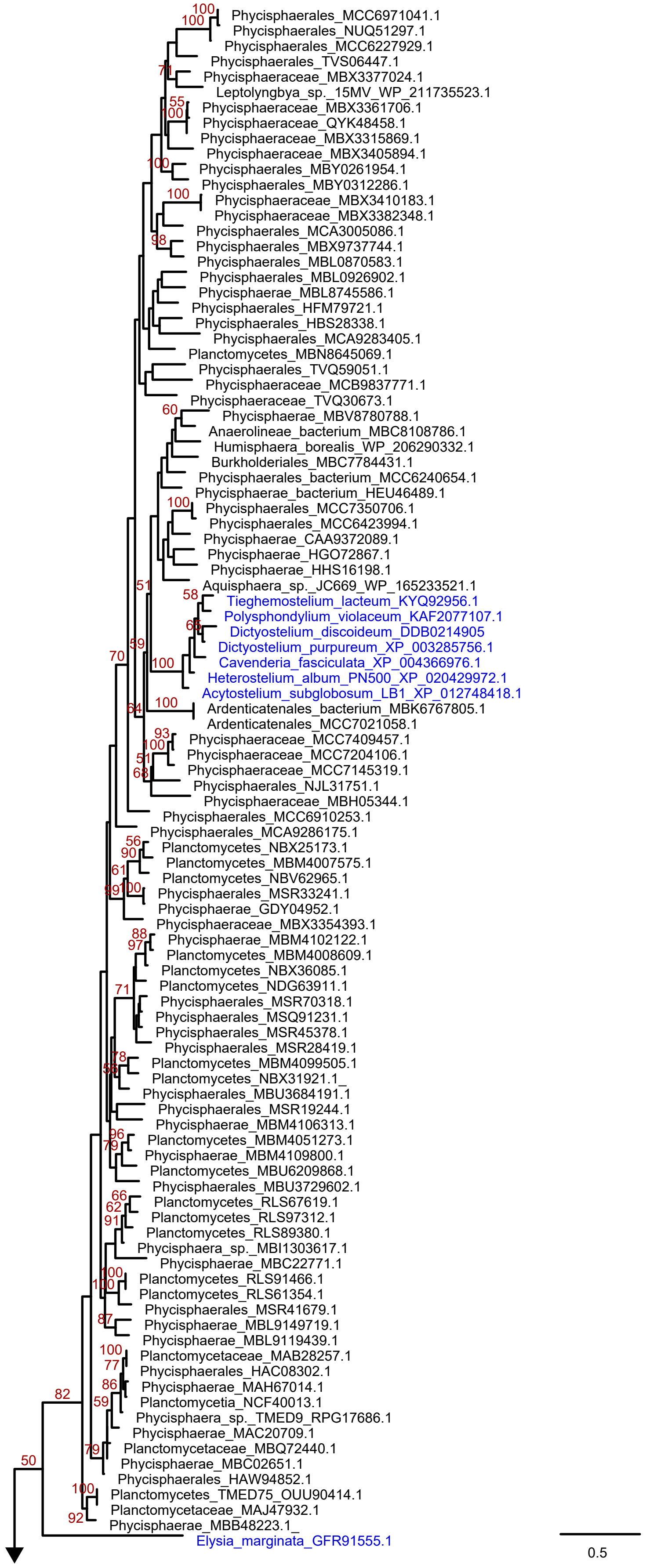

Supplementary Figure 7

b

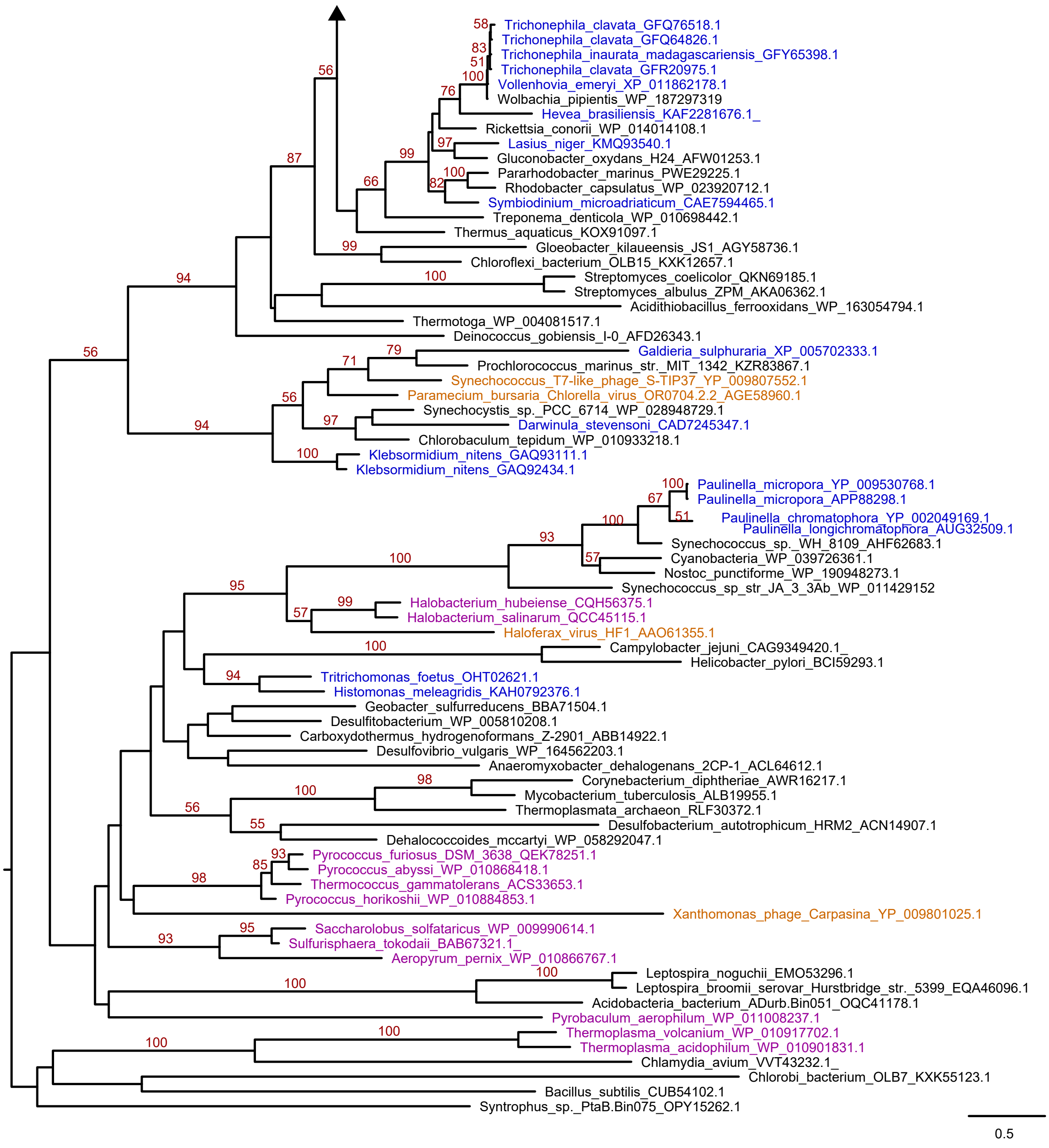

Supplementary Figure 8

dcd1 exon alignment and intron sizes

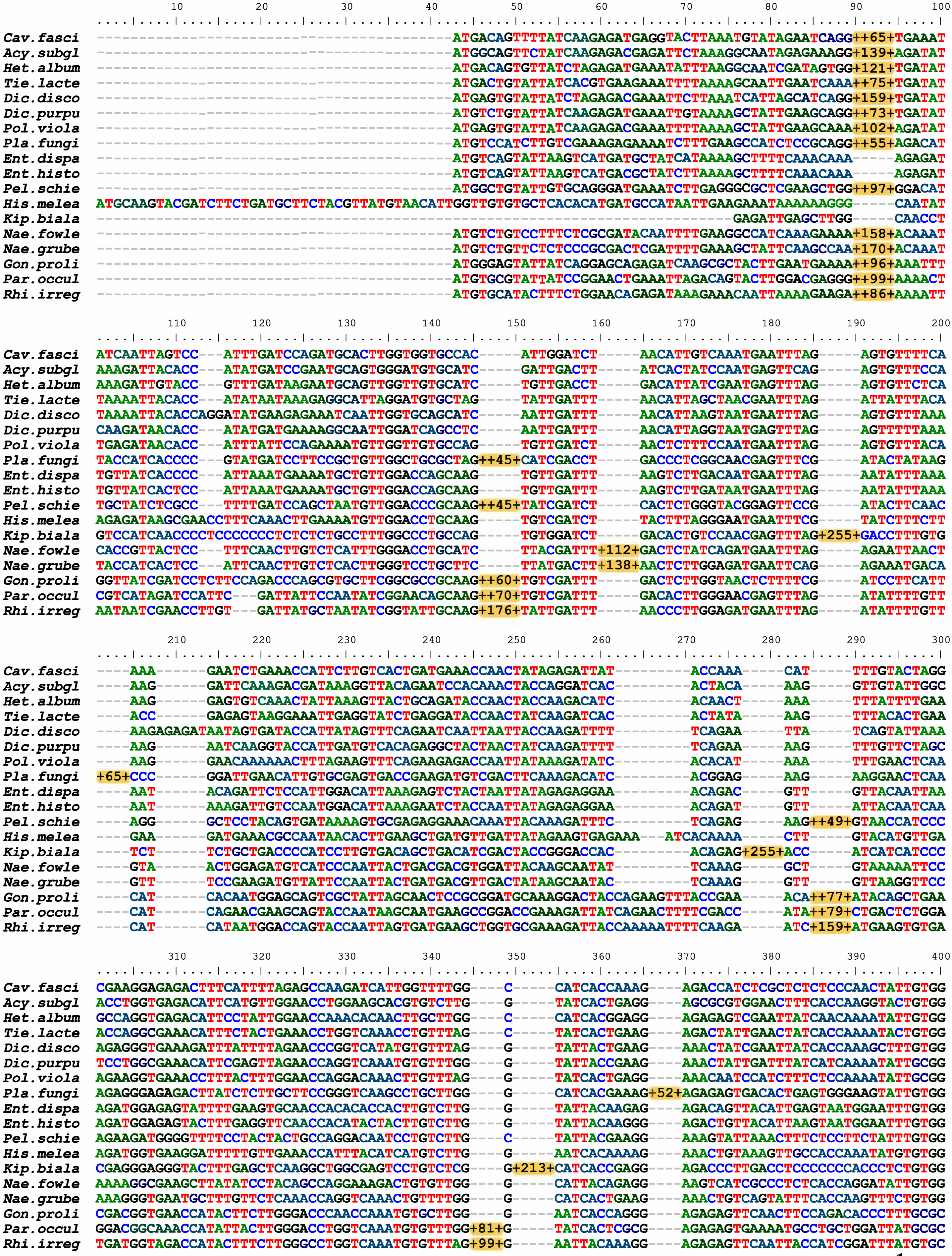

Supplementary Figure 8

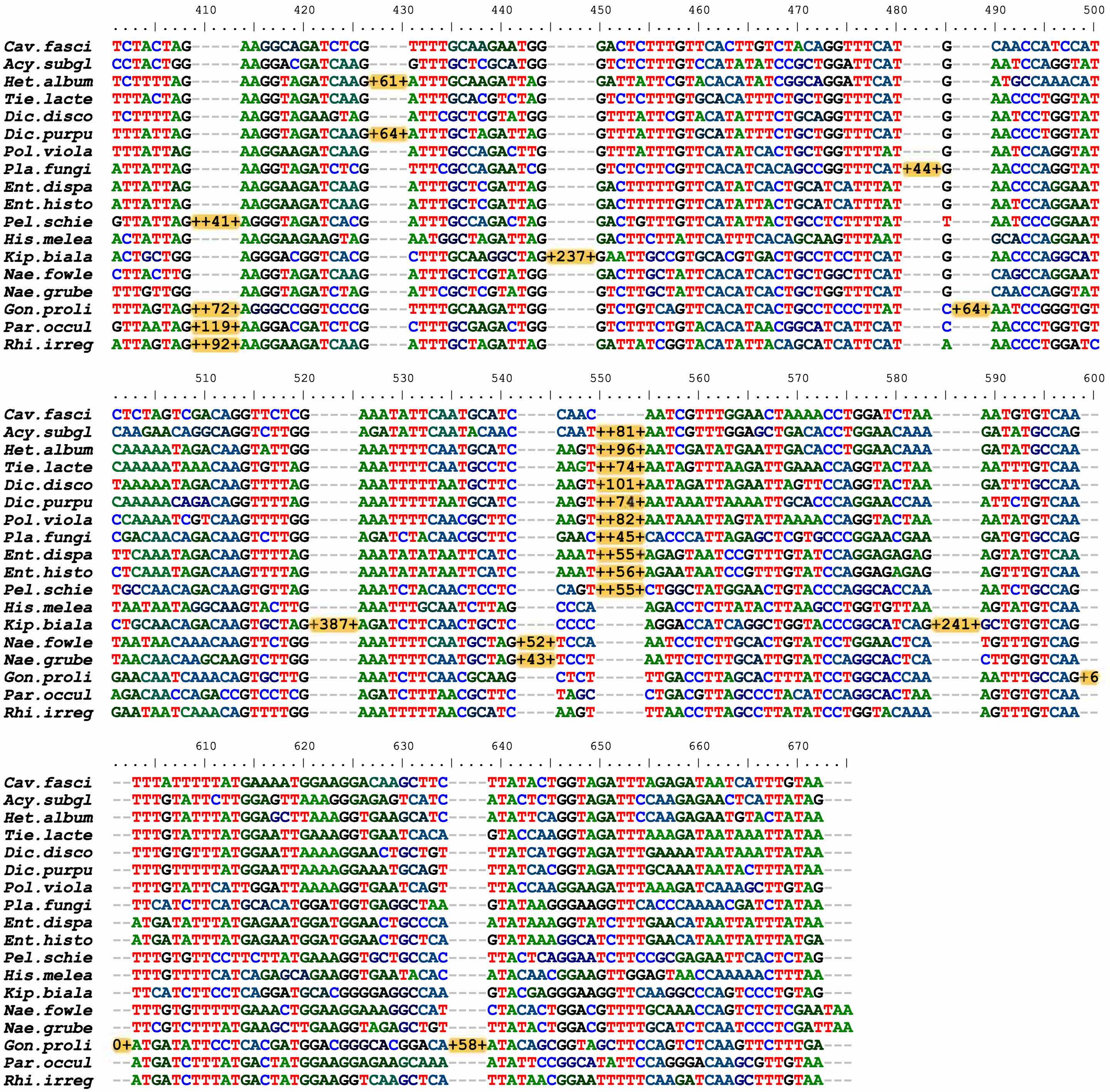

Supplementary Figure 8 *dcd2* **exon alignment and intron sizes**

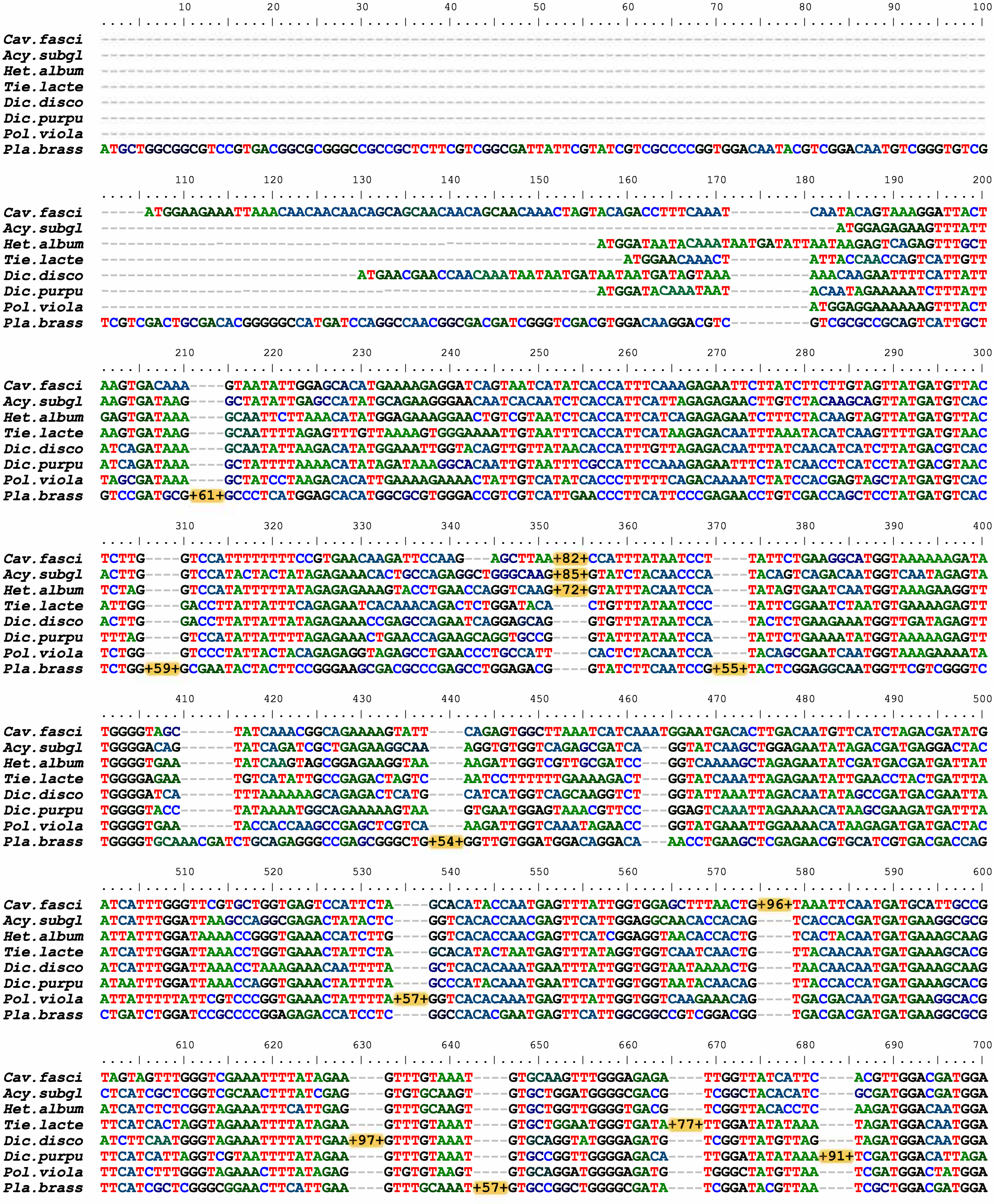

Supplementary Figure 8

```
                   710       720       730       740       750       760       770       780       790       800
          ....|....|....|....|....|....|....|....|....|....|....|....|....|....|....|....|....|....|....|....|
Cav.fasci GATTACCAACAACTCTGTACATCATACTATTCCACTA----ATCGTTGGACGTAGAATTGCACAGATTGTATTCTTTGATACCGATGGTACGATAAAGGA
Acy.subgl GATCACCAACAACTCACTCAGCTACTCGATCCCTCTG----GTCGTGTTCCGTCGCGTGGCCCAGATCATCTTCTTTGACACCGAGGGCATTGTC---GG
Het.album AATAACAAACAATTCTCTAAGTTATTCGATTCCATTG+74+GTTGTATTTAGAAGAATTGCACAAATTATATTCTTTGATACTGAAGGAATTGTT---GG
Tie.lacte AATTACAAATAATTCAAGAATGCATTCCATACCTTTA----GTAGTTGGTAGAAGAGTTGCTCAAATTGTATTTTTTGATACTGATGGTTGTGCA---GG
Dic.disco GATCACTAATAATAGTAGACACTATTCAATTCCATTA----GTAGTGGGTAGACGTTTAGCACAAATCGTTTTCTTTAATACTGACGGTATAGAA---AA
Dic.purpu AATCACCAATAACTCGCTCCACTATTCTATACCATTG----GTTGTTGGGAGAAGAATCGCTCAAATCATTTTCTTTGATTCCGAAGGAATTCTC---GA
Pol.viola GATTACCAACAACTCTAAACACTATTCCATTCCATTG----GTGGTGGGACGTAGAATTGCACAAATTATATTCTTTGACACAGAGGGTATTGAA---GG
Pla.brass GATCACCAACAACTCCCGGCACTATTCCATCCCTCTT----GTCGTCGGGCGACGCATTGCCCAGATCGTGTTCTTCGACACCCAGGGCACGCTGAACGG

                   810       820       830       840       850       860       870       880       890       900
          ....|....|....|....|....|....|....|....|....|....|....|....|....|....|....|....|....|....|....|
Cav.fasci AACATCATATGAAGGAAAGTATCAACAAGGTTACAATGGTGATATCAATCAATTAAAAGATAAATGGCATCCAAATGACATGCTACCAAAGAT----GTT
Acy.subgl CCCATCGTACGAGAGCAGCGGCAAGTATCAAGTGTCCAACGACGTCGAGTCCCTGAGGAAGCAGTGGCAGCCATCCGAGATGTTGCCCAAGAT----GTA
Het.album ACCATCCTATGAAAGCAGTGGAAAGTATCAAGTTTCAAACGATGTTGATGTGTTGAAGACCAAGTGGCACCCAACCGATATGTTACCAAAAAT----GTA
Tie.lacte TAGTTCCTATGAAAATACTGGAAAATATCAAACTTCAAATGATATCAATCAACTAAGAGAAAATTGGAAACCATCTGACATGTTACCAAAAAT----GTA
Dic.disco TAGACCCTATGAATCAAAGGGAAAATATCAAGTTTCTTCAGACGTTACAGAATTACAAAAAACTTGGAATCCATCTTTCATGTTACCAAAAAT----GTA
Dic.purpu TAAACCTTACGAGTCATCGGGTAAATATCAATCATCAAGTGATATCAATCAATTAAAAGAAAAATGGCAACCCACTGATATGTTACCAAAAAT----GTA
Pol.viola ACCCTCCTACGAGAGCAGCGGCAAATACCAGTCATCCAACGACATTGAGTCATTGAAAAAGGAGTGGTCACCCCAATGCATGCTTCCTAAAAT----GTA
Pla.brass GCGGTCGTATAGCAACAACGGCAAGTATCAGACCGCTGATACGCTCGAGGCGTTGAAACAGAAATGGTCGCCGTTTGATATGCTGCCCAAGAT+61+GTA

                   910       920       930       940       950       960       970
          ....|....|....|....|....|....|....|....|....|....|....|....|....|....|....
Cav.fasci TAACGATAAAGAGATTAAAAATAGATCACTTTCCACATTTTCAATGGATCAATACTCTAATATTAATCGTAAATAA---
Acy.subgl CAAGGATCGCGAGATCAAGAACAAGAATCCAGTCTTCTCAATCAGCGACTATGGTATCGGTGTAGATAAACAGTAA---
Het.album CAAAGACAAGGAAATCAAACAAAAACATCAATATCTTTCATTAGACGATTATATTATTGCAAAACAACTATAA
Tie.lacte TAAAGATAGAGAAATTAAAAATGGTACAAAAACATTTAATCCAAAAGATTATCCAAATCTATTAATAAAATAA------
Dic.disco TAAAGATAAAGAAATTTTAAATAGATCTCAAACATATAGCCCATCTCAATATTTTTTCGAAGGAAATTAA---------
Dic.purpu TAAAGATAAAGAACTGTTTAAAAACTTCCAAGCCTATAATCCATCAAACTATAATGTTTTCAAATAA------------
Pol.viola CAAAGACAAAGAGATCACCTGTAAAGTCCCCGCATTCAGTTTTGATAATTACAACTTCAACAAATAA
Pla.brass CCTTGACCGGGAACTTCGCAAGTCTCAAAAGCCCCATACAGGTAACAAGGCGCAGGAAGGCACCGCACGTTCAACGTGA
```

Supplementary Figure 8 *thyX* exon alignment and intron sizes

```
                     10        20        30        40        50        60        70        80        90       100
Cav.fasci  ------------ATGGAAGCTATAACTGACGGTATTACGGTGATTGAAACACCCAATGAAACAGAATACTATGATGTAATGAAAGGATCACATCGTTGGG
Acy.subgl  ---------ATGGACATGAACGAGATTGATAGCATTCAGATTACACAAGTACCTGCAGAGACAACATACTATGATGTCATGAAGGGTAGCCACCGTTGGG
Het.album  ---------ATGGACTCATTGGGTATCGATCAAATTCAAATCAACAAAGTACCAGCCGATACTGAGTACTACGATGTCATGAACGGTAGTCATCGTTGGG
Tie.lacte  ---------ATGATTCAAAACGAAATTGATCAAATTCAAATTAATATTACTAAACCAGAATGTGAATACTATGATGTTATGGGTGGAAGCCATCGTCATG
Dic.disco  ATGGGTCTTGATATTCAAACAGAAATCGATAAAATTGTAATTGAAAAGGTTAAACCAGAAGTTGAATACTATGACGTTATGGGTGGGTCACATAGATGGG
Dic.purpu  -----------------ATGATTGAAGAAAAAAATAGAAATTAATAAAATCCCAGCAGAAACCGAATATTATGATGTTATGGGTGGCTCACATAGATGGG
Pol.viola  ---------ATGGATTTAAATGAAATCAATAATATTAAAATTGAAGAGATTCCAGCCGAGTGTGAATATTATGATGTTATGAAGGGATCTCACAGACACA
His.melea  ---------------------------------------------------------------------------------------ATGGCTTTAGTTA
Tri.foetu  ---------------ATGAAACTTTTTGAGAGACATCAGATTGCGCCATGTTCTGAAAAAATCTGTACGTTAAGCATCTCATTTTTAATGGCTGTTGTTC

                    110       120       130       140       150       160       170       180       190       200
Cav.fasci  AGGTAAAAGTACACGATCATGGAAA-----TGTAGCTCTCGTTGATACTATGCCAAGATTGGCACCAGTTGGTCAAACTGCTGATTTTGCAATCTGTCAA
Acy.subgl  AGGTACCAGTTCACGACCATGGCAA+101+AGTTGCTCTGGTTGATGCTATGCCAAGACTGGCACCAGAGGGACAGACTGCCGACTTTGCTATCGTCCAG
Het.album  AAGTAAAGGTACACGATCATGGAAA++91+AGTTGCATTGGTTGATTCTATGCCAAGACTTGCACCAGTTGGTCAAACTGCTGATTTCGCCATCGTTCAA
Tie.lacte  AAGTAAAAGTACACGATCATGGAAG-----TGTAGCATTGGTAGATTGTATGCCAAGACTTGCACCAGTAGGTCAAACTGCTGATTTCTCTATTGTACAA
Dic.disco  AAGTCAAAGTGCACGACCATGGTAA-----AGTTGCATTAGTCGATACTATGCCAAGATTAGCACCAGTTGGTCAAACCGCCGATTTCTCAATTTGTCAA
Dic.purpu  AAGTCAAAGTACATGACCACGGTTC-----AGTTGCATTAGTTGACTCTATGCCAAGATTAGCTCCAGTTGGTCAAACTGCTGATTTTGCTATTTGCCAA
Pol.viola  GTGTCAAGGTTCATGATCATGGCAG-----TGTTGCATTGGTGGACAGCATGCCCAGATTGGCACCAGTTGGACAAACAGCTGATTTTGCCATTGTCCAA
His.melea  AGTTAATAGCTCACACTCCAGAGCC-----TGAAAAGGTAATTGCAATAGCAGCACGATTTTGT---------------------TATTCAAAGTTTTCA
Tri.foetu  GCTTAATTGCACATACTCCAAACCC-----AGAACGCGTTGTTGCTGTTGCCTCCAGGCTCTGC---------------------TACTCAAAGTTCGAT

                    210       220       230       240       250       260       270       280       290       300
Cav.fasci  GCTGCCAGAGTATCCTATGGTGCTGGAACTAAAAAGGTTACTGAAGATACTGGTCTCATTAGATACCTATATCGTCATCAACATACTT+77+CACCATTT
Acy.subgl  GCTGCCCGTGTGTCTTATGGCGCTGGCACTAAGAAGGTCAGTGAGGACAAGGGACTGATCAGATACTTGTTCAGACATCAACACACCT----CGCCATTC
Het.album  GCCGCTCGTGTATCCTATGGTGCCGGTACCAAGAAAGTAACTGAAGACAAAGGTCTTATCCGTTATCTCTATAGACATCAACATACTT+77+CACCATTC
Tie.lacte  GCTGCAAGAGTCTCCTATGGTGCAGGTACCAAAAAAGTCTCCGAAGATAAAGGTTTAATTCGTTACCTATTCAGACATCAACATACAT+82+CACCATTT
Dic.disco  GCAGCAAGAGTGTCATATGGTGCCGGTACTAAAAAAGTCACTGAAGATAAAGGTTTAATTCGTTATCTTTATAGACATCAACATACTT+91+CACCATTT
Dic.purpu  GCTGCCAGAGTATCATATGGTGCCGGTACTAAAAAAGTTTCAGAAGATAAAGGCTTAATTCGTTATTTATACAGACACCAACATACTT+57+CACCATTT
Pol.viola  GCAGCCAGAGTGTCCTATGGTGCTGGCACTAAAAAGGTCTCTGAAGACAAGGGATTGATTCGTTATTTATTCAGACACCAACACACAT----CACCTTTT
His.melea  ACAGATCAAATTATAGAGAAAATGACCCAAGATGAAATCAAAAAAAGT---------ATTCGGCACTTAAACAAATTAGGACATGAAT----CACCAATT
Tri.foetu  GCTGAGGAAATTTTAAAGACAATGACAGACGAAGATGTTGAAAAAAAT---------ATTCGATTGCTTAACGAAATGAAACATGAGT----CACCAATA

                    310       320       330       340       350       360       370       380       390       400
Cav.fasci  GAAATGATTGAATTTAAATTCCATTGTGTC---ATGCCAATCTTTATTGCTAGACAATGGATTAGACATCGTACTGCCAATGTCAATGAATACAGTGCTC
Acy.subgl  GAGATGATTGAGTTCAAGTTCCACTGTGTC---ATGCCAGTCTTCATTGCTCGTCAGTGGATCAGACACAGGACCGCCAGCGTCAACGAGTACAGTGCCC
Het.album  GAGATGATTGAATTCAAATTCCATTGTGTG---ATGCCAGTTTTCATTGCTAGACAATGGATTAGACATAGAACAGCCAATGTCAACGAGTACAGTGCCA
Tie.lacte  GAAATGGTTGAATTTAAATTTCATTGTGTA---ATGCCAGTTTTCATTGCTAGACAATGGATTAGACATAGAACTGCTAATGTTAACGAATACTCTGCTA
Dic.disco  GAAATGGTAGAATTTAAATTTCATTGTGTA---ATGCCAGTATTTATCGCACGTCAATGGATTAGACATAGAACAGCAAATGTCAATGAATATAGTGCAC
Dic.purpu  GAAATGGTAGAATTTAAATTTCACTGTGTA---ATGCCAGTATTTATTGCACGTCAATGGATTAGACATAGAACTGCCAATGTTAACGAATATAGTGCTC
Pol.viola  GAAATGGTTGAATTCAAATTCCACTGTGTG---ATGCCAGTCTTTATCGCTCGTCAGTGGATCAGACATCGTACTGCCAACGTCAACGAATACTCTGCTC
His.melea  GAACATGCTTCATTTACATTTAGTATTTCAAATATTTCACGAGCTTGTATGGCTCAATTAACCAGACATAGAATTGCATCTTTCTCAGTAAAATCGCAAA
Tri.foetu  GAACATGCTTCGTTTACTTACAGTGTTACAGGTATTTCACGCGCATGTATGGCTCAATTCACAAGACACAGAATTGCGGCATTTTCTGTCAAATCTCAAC

                    410       420       430       440       450       460       470       480       490       500
Cav.fasci  GTTATTCAGTCATGCCAGACAAGTTCTATCATCCATCGGTCGAAGATGTCAGAAAACAATCAACATCCAATAGACAAGGTGGTGAAGATCAAATGGATAC
Acy.subgl  GTTACTCTGTTATGCCCGACAAGTTCTATCATCCATCGGCTGAGGACGTGAGAAAGCAGTCGACCACCAACCGCCAGGGTGGTGAGGAGCCAATCGACAC
Het.album  GATACTCGGTTATGCCAGACAAGTTCTACCATCCATCGGCCGAAGACGTCAGAAAGCAATCGACTTCCAATCGTCAGGGTGGTGAGGATGCCACCGACAC
Tie.lacte  GATATTCTGTTATGCCAGACAAATTTTATCATCCCAAAGTTGAAGATGTCAGAAAACAATCAGCTAGCAACAGACAAGGTGGAGAGGAACAACTCGAAAC
Dic.disco  GTTATTCAGTATTACCAGATAAATTTTATCATCCATCAATTGAGGAAGTTAGAAAGCAATCAACATCAAATAGACAAGGTGGTGAAGAAGCATTGGAGCC
Dic.purpu  GTTATTCAGTAATGCCAGATAAATTCTATCATCCATCTGCCGAAGATGTAAGAAAACAATCTGTTTCAAATAGACAAGGTGGTGAAGAACAATTAGAAGC
Pol.viola  GTTACAGTGTCATGCCCGACAAATTCTATCATCCATCTGCCAACGACGTTCGTAAGCAATCTGTGTCCAACAGACAAGGCGGTGAAGAGCCACTCGACAC
His.melea  GATAT---GTCAATGAAAAGAAATTTACATTTATCACTCCACCAGAACTTAAAGATGATAAAGAA-----------------------------------
Tri.foetu  GATAC---GTTAATGAAAAGAAATTCAAATATGTTTGTCCTCCTGGACTTGAAGACGATGAATTA-----------------------------------

                    510       520       530       540       550       560       570       580       590       600
Cav.fasci  TAAAACTGCTCAAGAG+86+TTTTTAGATTATTTAGATCAAGCTGAAGAATGTTACAAGACCTATAATAACTTGTTGGATAAAGGAGTTAGTAGAGAATT
Acy.subgl  CAAGACCGCCCAAGAG+85+TTCTTGGACTACCTTGACCAATGTGAGGCATCATACAAGACCTACTCTGGACTGCTCGAGAAGGGAGTGTCACGTGAGCT
Het.album  CAAGACCGCACAGGAG----TTCCTCGACTATCTCGACCAGGTCGAAGCCAACTACAAGACCTACTCAGAGTTGCTCGACAAGGGTGTCTCTCGTGAGTT
Tie.lacte  TAAGACTGCAGAAGAA----TTTCTTGCCTATTTGGATCAAGTAGAGAGCTCTTACAAGACCTATAATGAACTTTTGGATAAGGGCTTGAGTAGAGAACT
Dic.disco  AAAAACCGCCCAAGAG----TTTTTAGACTATTTAGATAAAGTTGAAGAAAACTATAAAACCTATAATGAACTCTTAGAGAAAGGTTTATCACGTGAATT
Dic.purpu  CAAAACTGCCCAAGAG----TTCTTAGACTATTTGGATAAAGTTGAAGAAAACTATAAAACCTACAACAATCTCTTAGAAAAAGGCTTATCACGTGAACT
Pol.viola  TAAAACTGCCGAAGAG----TTTTTAGAGTACTTGGATCAAGTCGAAGCCTCTTACAAAACCTACACCAATCTCTTGGACAAAGGATTGTCTCGTGAGCT
His.melea  -----------------------------TATCAATTAATAATGAAGAAGCTTCACGATTCATATGCTCGACTAAAGAAAAAAGGAATAGCTAATGAAGA
Tri.foetu  -----------------------------TACAAGAAAATTATGAAAAATCTTCACAAAGCATACCAACAACTTAAAAAGAAGGGATATCAAAATGAAGA

                    610       620       630       640       650       660       670       680       690       700
Cav.fasci  GGCACGTATTGGGTTACCGGTAAGCGTGTACACTGAATGGTACTGGAAGATTGATCTACACAATCTATTCCATTTCCTTCGTTTGCGTATGGACAGCCAC
Acy.subgl  CGGTCGCATTGGCCTGCCTGTCAGCGTGTACACTGAGTGGTACTGGAAGATTGACCTCCACAACCTGTTCCACTTCCTCAGACTGCGCATGGACTCTCAC
Het.album  GGCACGTATCGGTTTGCCAGTCTCTGTGTACACCGAGTGGTACTGGAAGATCGACTTGCACAACCTCTTCCACTTCCTCCGTCTCAGAATGGATTCACAC
Tie.lacte  TGGTAGAATTGGTTTACCAGTTAGTGTATACACTGAATGGTACTGGAAAATTGATTTACACAATCTCTTCCATTTCCTTAAATTGCGTATGGACTCACAC
Dic.disco  GGGTCGTATTGGGTTACCAGTTAGTATCTACACTGAATGGTATTGGAAGATCGATTTACATAATCTTTTCCATTTCTTAAGACTTCGTATGGATAGTCAT
Dic.purpu  AGGTCGTATTGGTCTCCCAGTCAGCATTTACACCGAATGGTATTGGAAAATCGATTTACACAATCTTTTCCATTTCCTTAGACTTCGTATGGATAGCCAT
Pol.viola  TGGTCGTATTGGTTTACCCGTGAGTGTGTACACTGAATGGTATTGGAAAATTGATCTTCACAATCTCTTCCACTTTCTCAGACTCCGTATGGACTCTCAC
His.melea  TGCAAGGTTTGTCCTTCCAAATGCATGCACTACACAACTTATCACAACAATGAATGCAAGATCATTGAAAAACTTTTTTGCACTCAGATGTTGTTACCGT
Tri.foetu  TGCGCGCTTTGTTTTACCAAATGCATGTGACACACAACTTATCGTCACAATGAATGCTCGTTCACTAAGGAACTTCTTCACCTTAAGGTGTTGTCACAGA
```

Supplementary Figure 8

```
                        710       720       730       740       750       760       770       780       790       800
               ....|....|....|....|....|....|....|....|....|....|....|....|....|....|....|....|....|....|....|....|
Cav.fasci      TCACAAAAGGAGATTAGAGATTATGCCAATGCTATCTTTGCCCTCATTCGTCCTATTGTCCCAGTTGCATGTGAAGCCTTTATGGACTATGCCTTTGAGA
Acy.subgl      TCACAGAAGGAGATCAGAGACTATGCCAACACCATCTTTGCTCTGATACGCCCAATCGTCCCCATCGCCTGTGAGGCCTTCCTCGACTACTCCTTCGAGT
Het.album      TCGCAAAAGGAGATTCGTGACTATGCCAACACCATCTTTGCTCTGATCAGACCGATTGTACCGGTTGCCTGTGAGGCATTCATCGACTATGCTTTCGAGT
Tie.lacte      AGTCAAAAGGAAATTAGAGATTATGCCAATACTATTTTTGCTCTCATTAGAAAAATTGTACCAGTTGCTTGTGAAGCCTTTATGGATTACTCTTATGAAT
Dic.disco      TCTCAAAAAGAGATTAGAGATTATGCAAATACAATATTTGCTCTCATTCGTCCAATTGTACCAGTTGCTTGTGAAGCATTTATAGATTATGCTTTTGAAA
Dic.purpu      TCACAAAAAGAAATTAGAGACTATGCAAATACTATTTTTGCTCTAATCAAACCAATTGTTCCAGTTGCTTGTGAAGCTTTTATGGACTATGCCTTTGAAA
Pol.viola      TCTCAAAAGGAAATTAGAGACTATGCCAACACTATTTTTGCTCTCATTCGTAATATTGTCCCCGTTGCTTGCGAAGCATTTGTCGACTACTCTTTTGAAT
His.melea      GCTCAATGGGAGATTCGTGATGTTGCAGATAAAATGTTGAAACAATGTAAAGAAGTAGCTCCTATTTTATTTGAAAATGCTGGCCCTCCATGCTCGCAT-
Tri.foetu      GCACAATGGGAAATCAGAGATGTTGCTAACCAAATGCTTAAAGAGGCGAAAAAAGTTGCACCAATTATTTTTGAAAATGCTGGACCAAATTGCTTCCGTG

                        810       820       830       840       850       860       870       880       890       900
               ....|....|....|....|....|....|....|....|....|....|....|....|....|....|....|....|....|....|....|....|
Cav.fasci      GTGTCAAGTTGACTCGTCTCGAAATTGAAGCCATCCGTACCGGAAAACCAATTCAATCGACCAACAAACGTGAAACTGAAGAATGGATTCAAAAGGCTAC
Acy.subgl      CGGTCCGTCTGACCCGTCTGGAGATTGAGGCCATCCGCACTGGCAAGCCACTCAACACTAGCAACAAGCGTGAGACTGAGGAGTGGGAGTCCAAGAGGAC
Het.album      CGATCAAACTCACACGTCTCGAGATCGAAGCAATCCGTTCCGGTAAGCCATTGAACACCGCCAACAAACGTGAGACCGAAGAGTGGGAAGCCAAACGTAA
Tie.lacte      CTATTAAATTGACCAGACTGGAAATCGAAGCTATTCAAACTGGTAAACCATTGAATACTACCAATAAGAGAGAAACCGATGAATGGGAACAAAAGAGAAA
Dic.disco      GTTTAAAACTTACACGTTTAGAAATTGAAGCAATTCGTACTGGTAGTCCACTCAATACTACAAATAAAAGAGAAATAGAAGAATTTGAAGAAAAGAAGAA
Dic.purpu      GTATTAAATTAACACGTTTAGAAATCGAAGCTATTCGTTCTGGTAAACCAATCAATACTACAAATAAAAGAGAAACTGAGGAATGGGAACAAAAGAAACA
Pol.viola      CCATTAAATTGACTCGTTTGGAAATTGAAGCCATTCGTTCTGGTAAACCAATCAATACAACAAACAAAAGAGAAGCTCAAGAATGGGAAGATAAGAGAAA
His.melea      --------------------------------TGTCGCGAAGGAGAACTTGCTGCAAATTGTCCAAGAAAGAAGAAGTTTTCTAAATAA-----------
Tri.foetu      GACCA---------------------------TGCCCTGAAGGAAAACTTGGTGAAACATGCCCATTCAGAAATAAAAAAGGTAAGAAGAATAATAAAAA

                        910       920       930       940       950       960       970       980
               ....|....|....|....|....|....|....|....|....|....|....|....|....|....|....|....|..
Cav.fasci      TTTATTGAATCTTACTGATCATATTCCATCAAAAGATCAACAACAGCAAGAAGAAAAGAAACAATAA
Acy.subgl      AATGCTTGGCCTGCCAGCTCCCGTCAAG---GAGGGAGAGCAGCCAGCTCAGCAGCAACAGCAAGAGACCAAGCCACATGCC
Het.album      ACAACTAGGATTACCAGAGCCAGTCAAAGTCGAACAAACACAACCATCATCAACTGAACAAGCATAA
Tie.lacte      ACTTTTAAATATACCAGCACCAGTGGTACCACAAACTAATACAACTACTACACCACCAACTGAATCTAAATAA
Dic.disco      ATTATTATTCCCAAATACTCAAGCCTAA
Dic.purpu      ATTATTAGGTTTG---------------------GATAAAACCTCACAAGCATAA
Pol.viola      ACAATTGGGTTTACCTGAACCAGATGAACAACCAAAATCTGAATCTGCTTAG---
His.melea      ----------------------------------------------------------------------------------
Tri.foetu      ATAA------------------------------------------------------------------------------
```

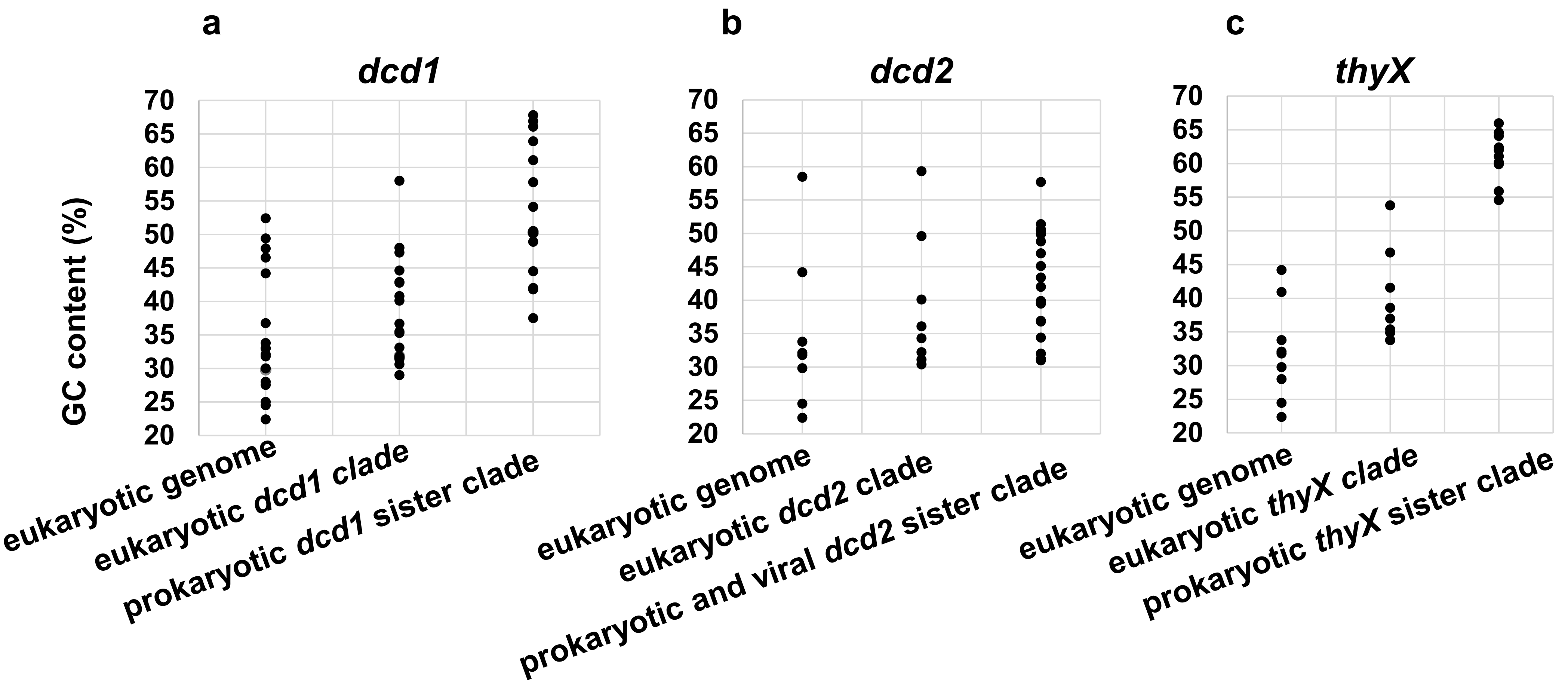

Supplementary Figure 9

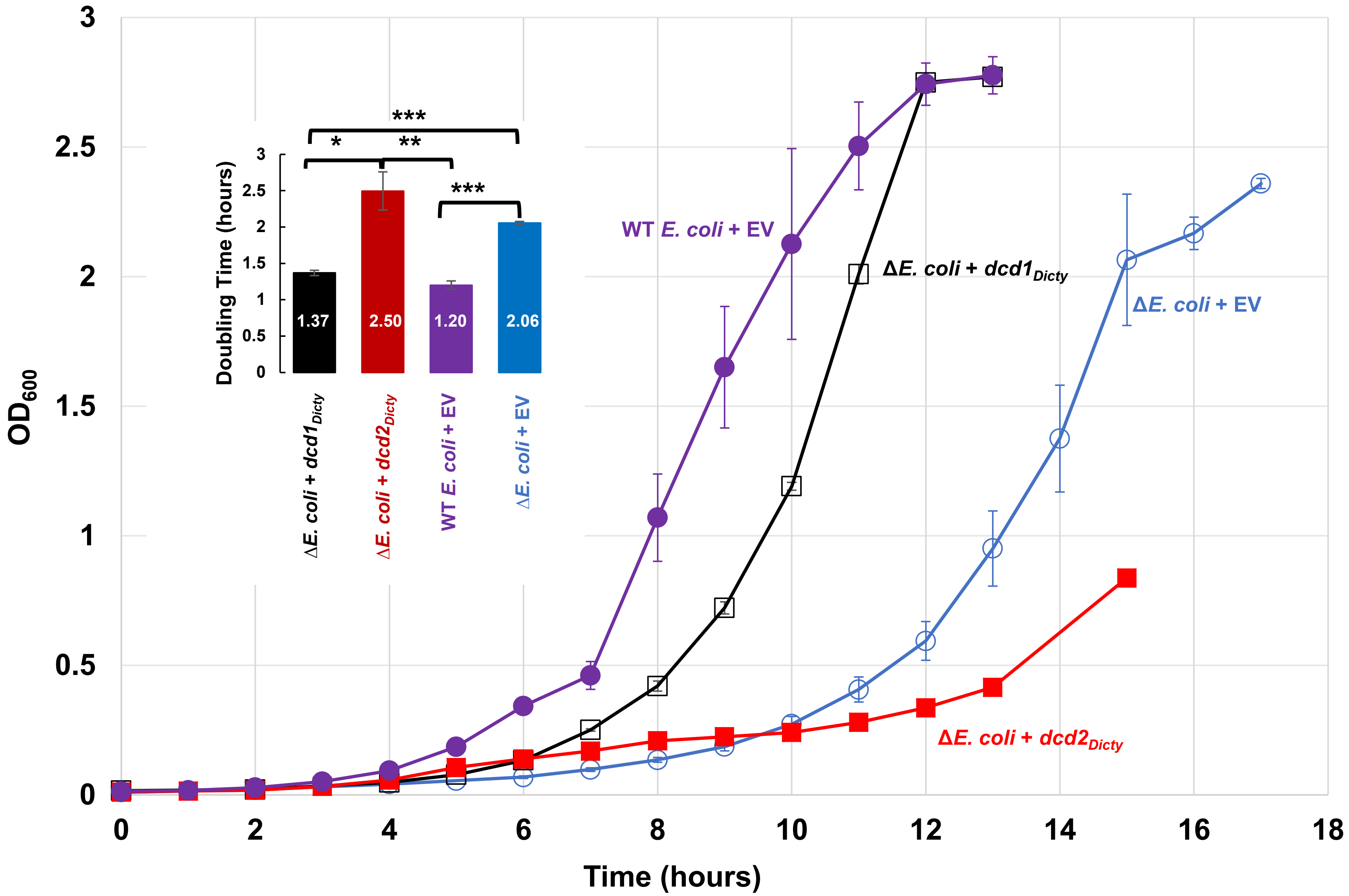

Supplementary Figure 10

Part 1 Constraint1

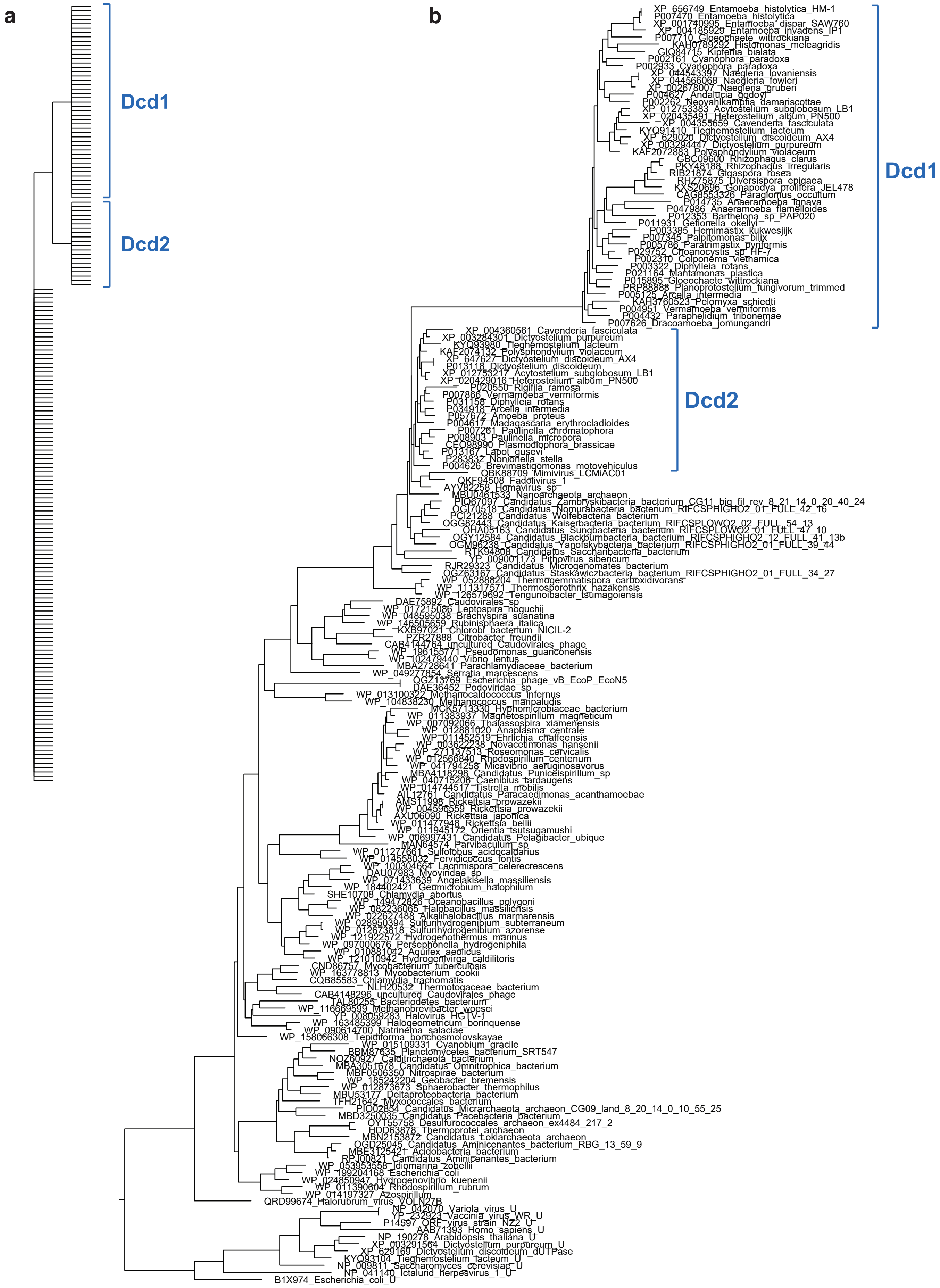

Supplementary Figure 11

Part 1 Constraint2

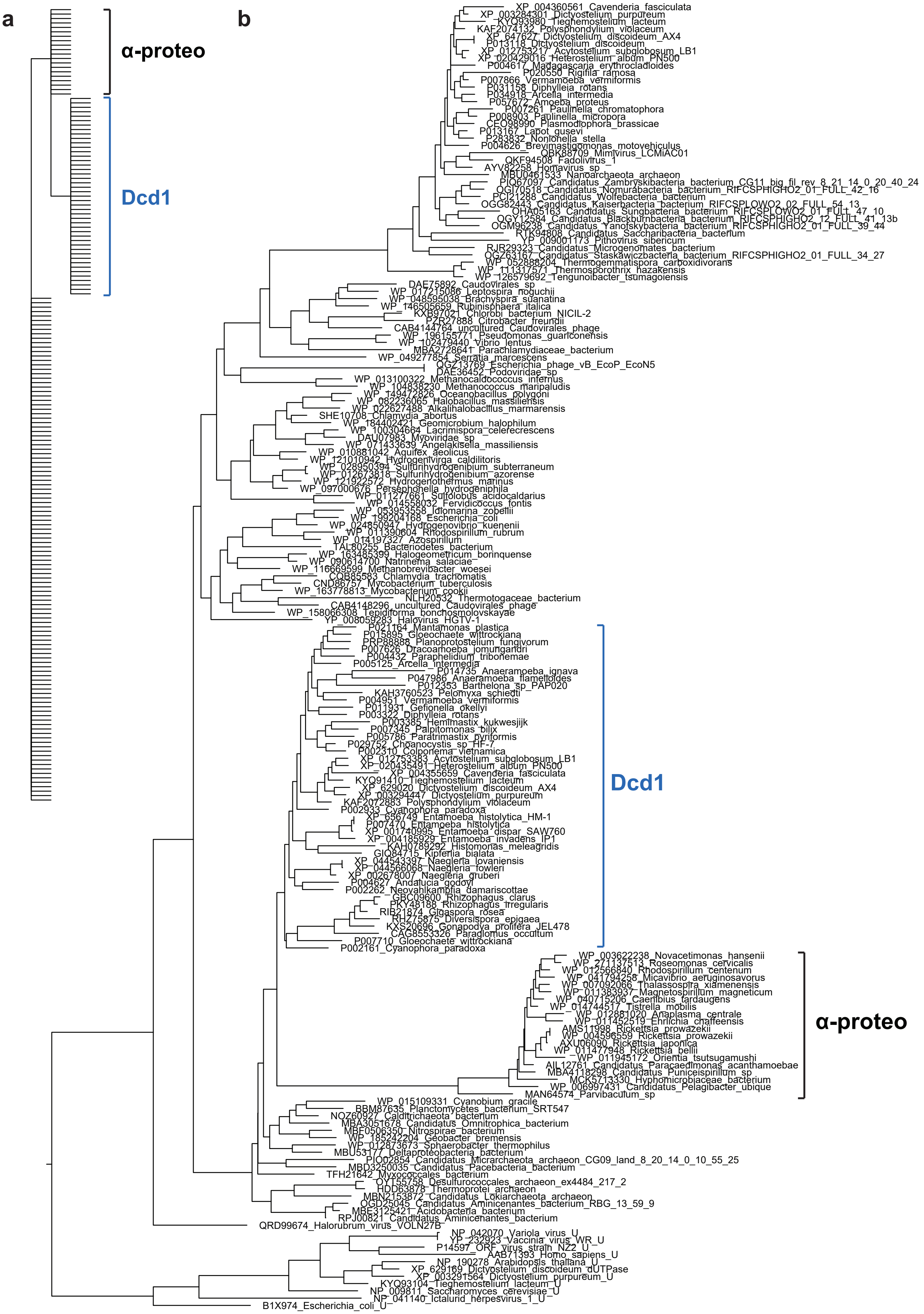

Supplementary Figure 11

Part 1 Constraint3

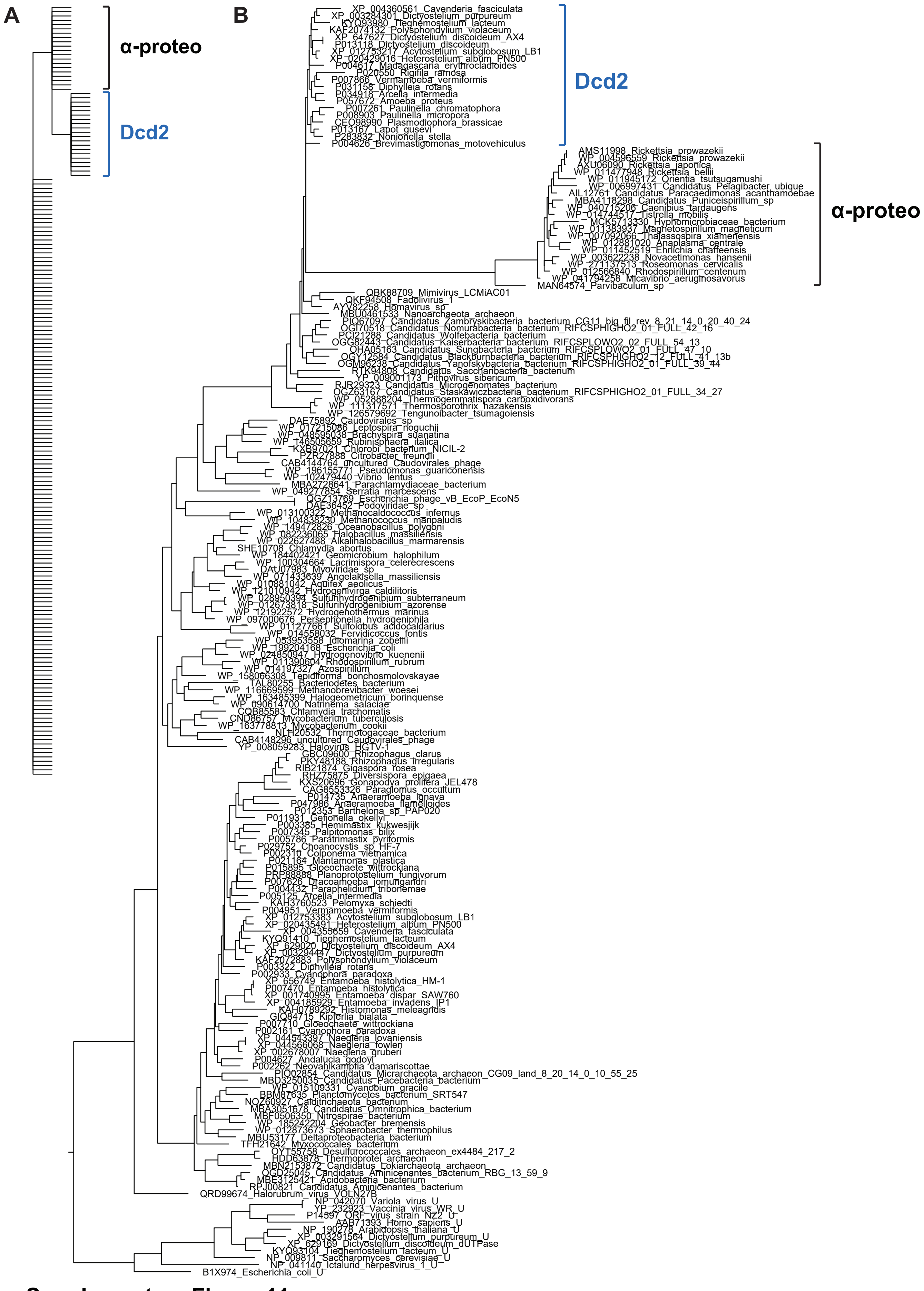

Supplementary Figure 11

Part 1 Constraint4

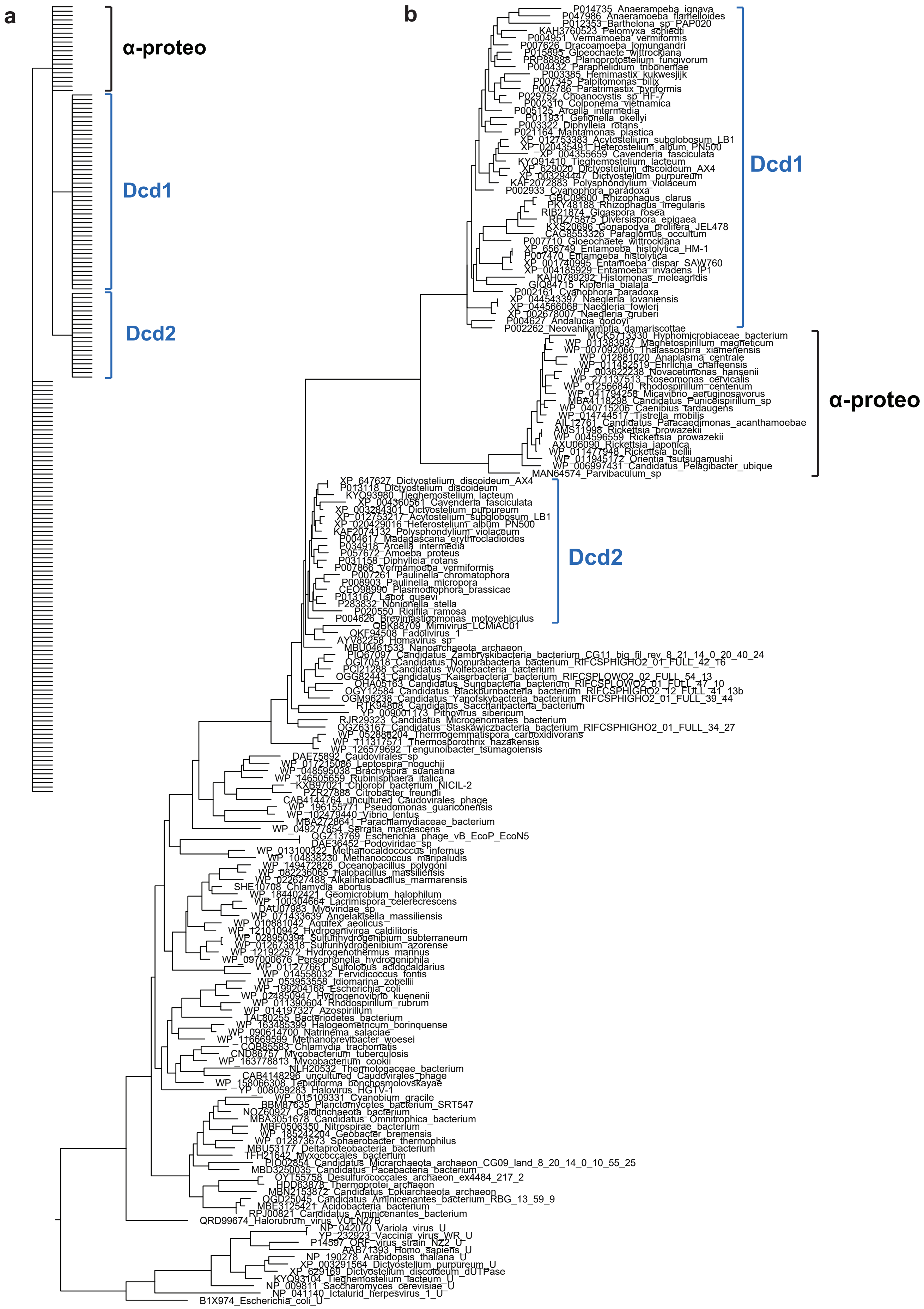

Supplementary Figure 11

Part 2 Constraint1

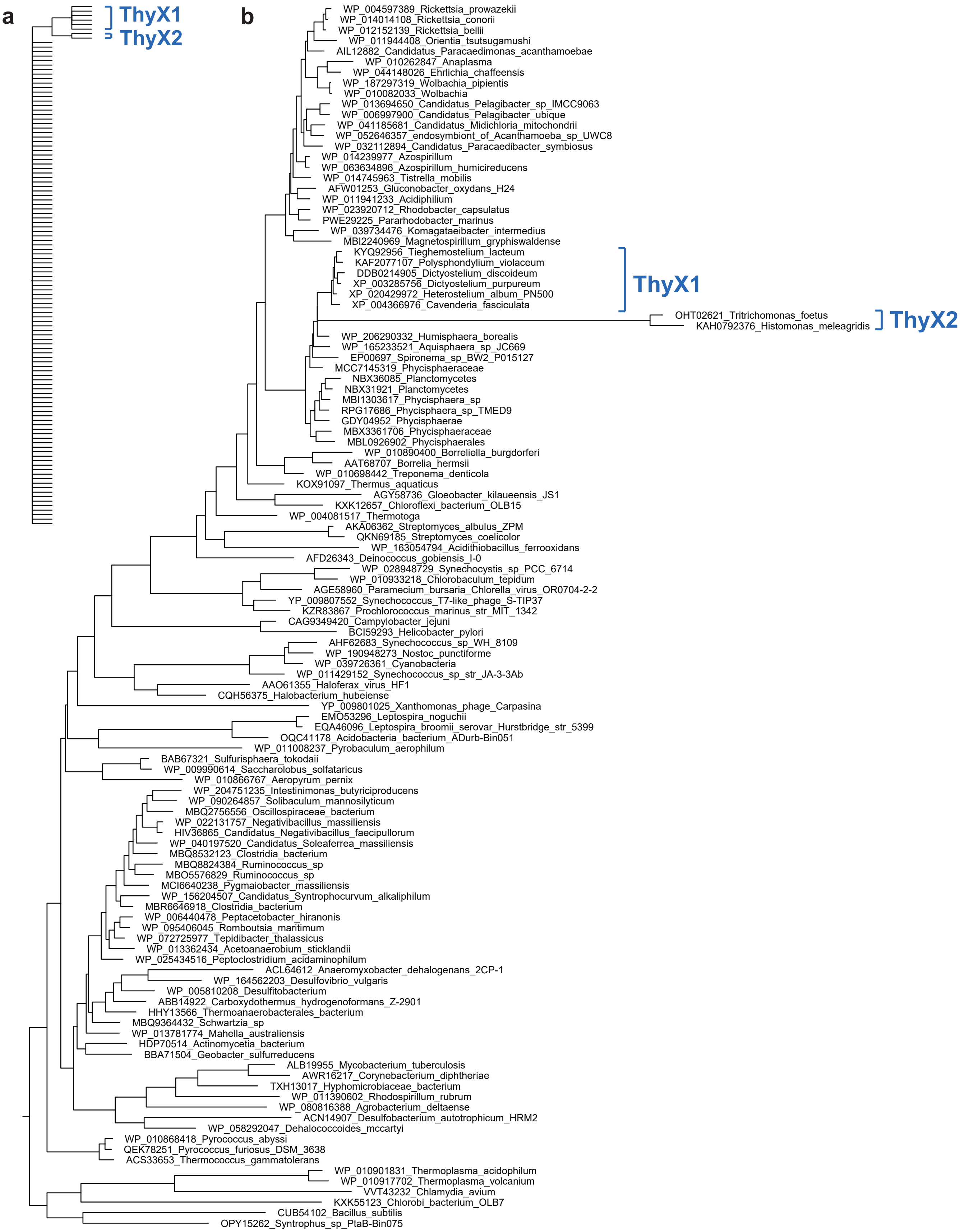

Supplementary Figure 11

Part 2 Constraint2

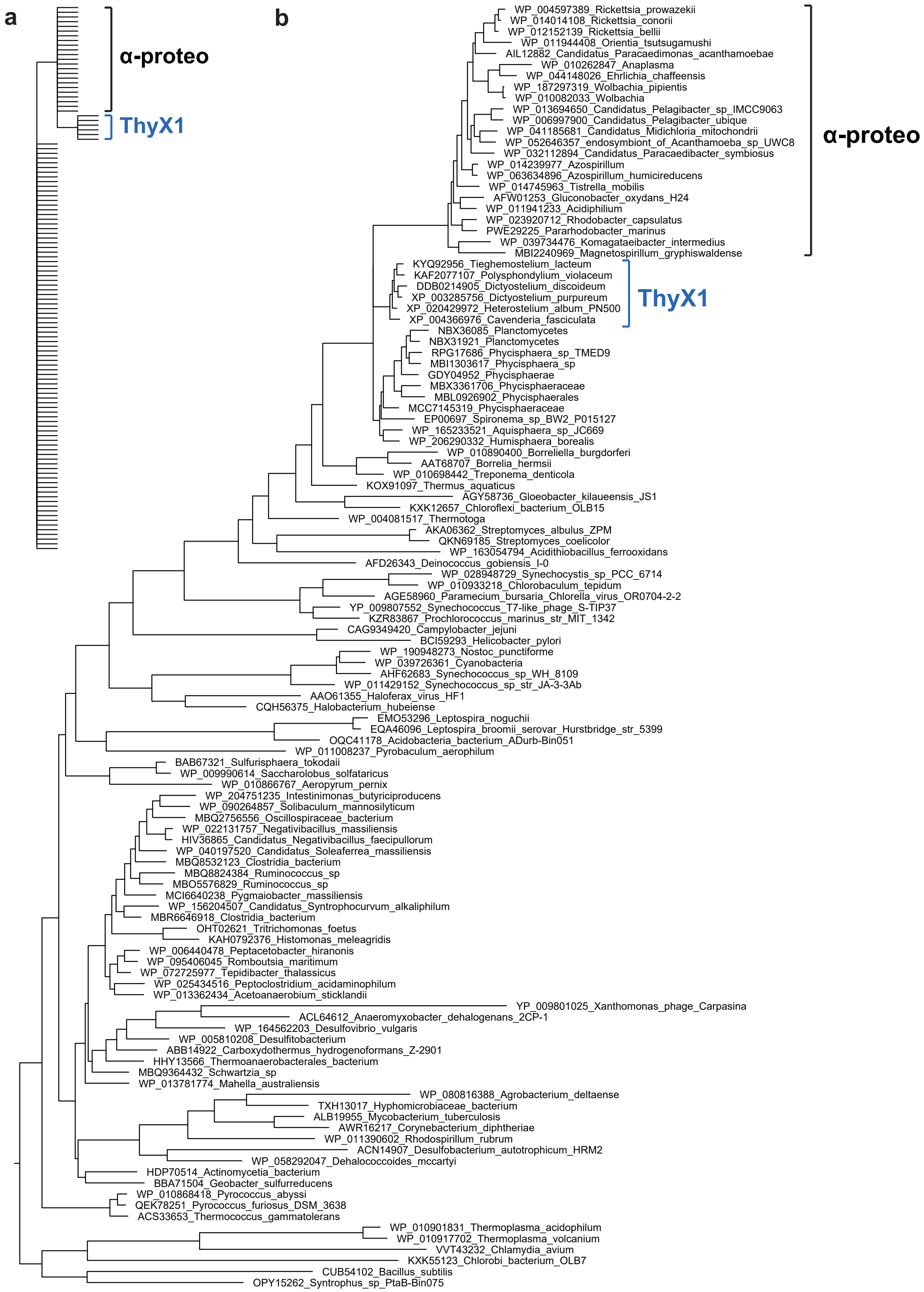

Supplementary Figure 11

Part 2 Constraint3

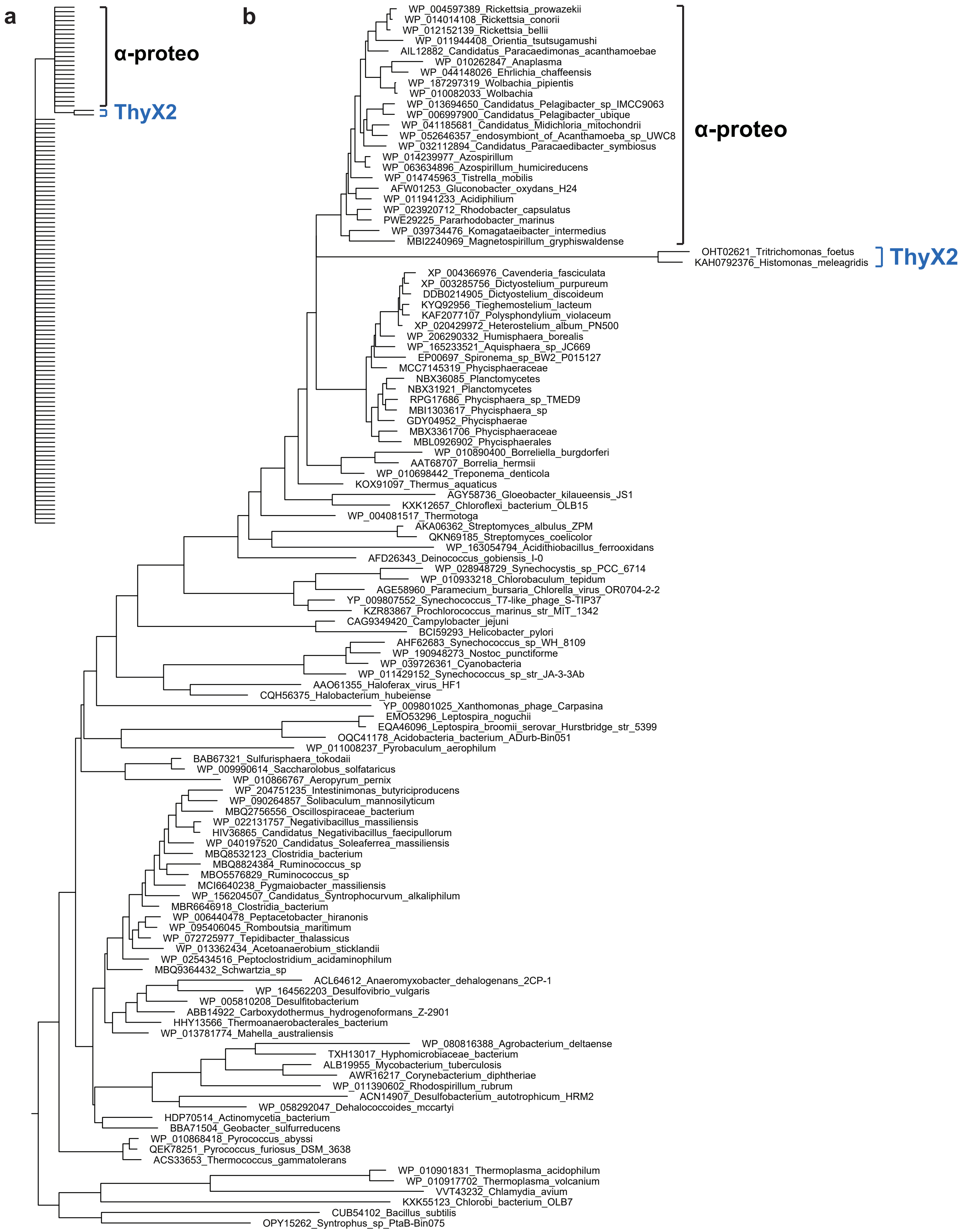

Supplementary Figure 11

Part 2 Constraint4

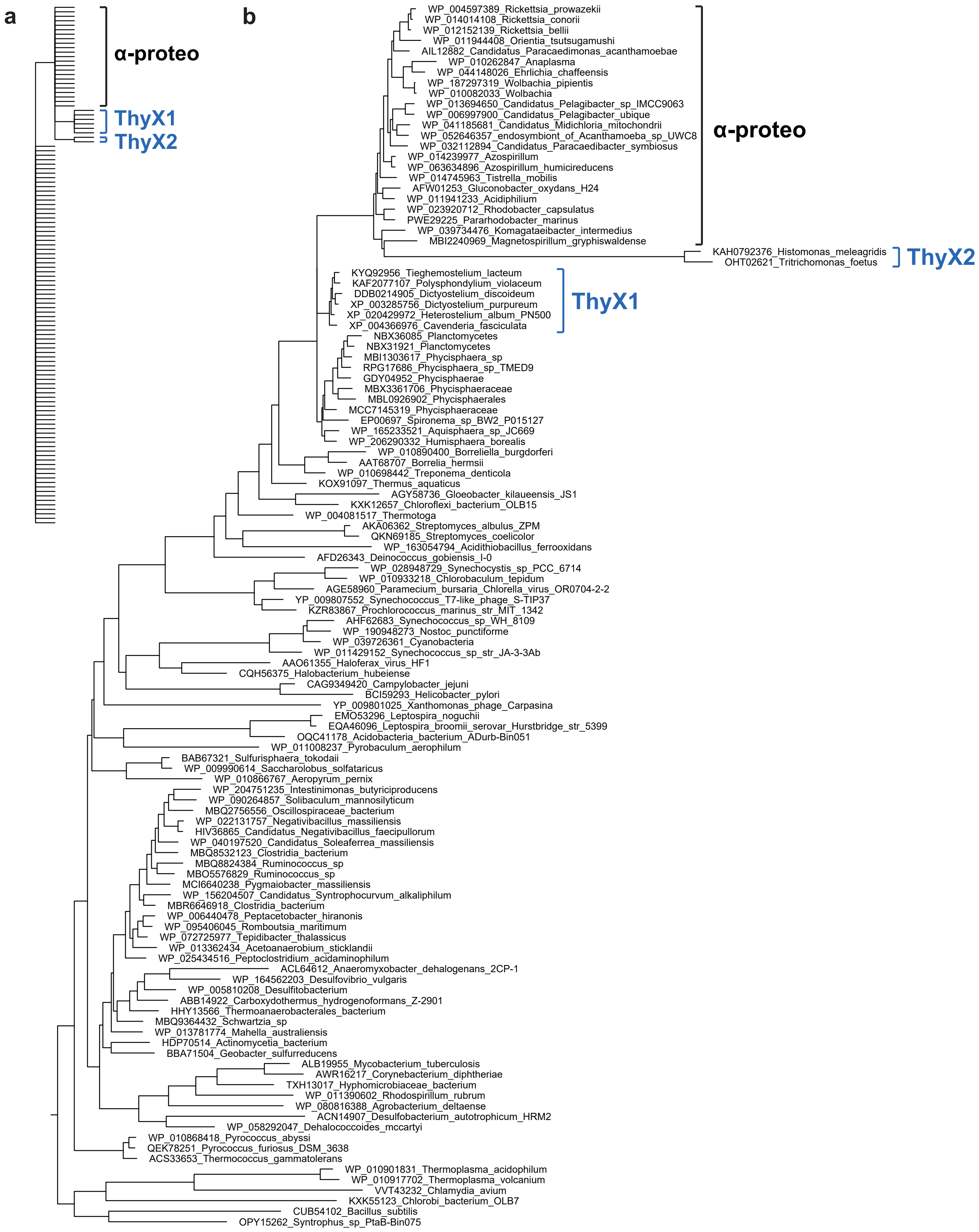

Supplementary Figure 11

Supplementary Figure 12
Dcd ML tree with RefSeq Archaeal Dcd sequences (purple)

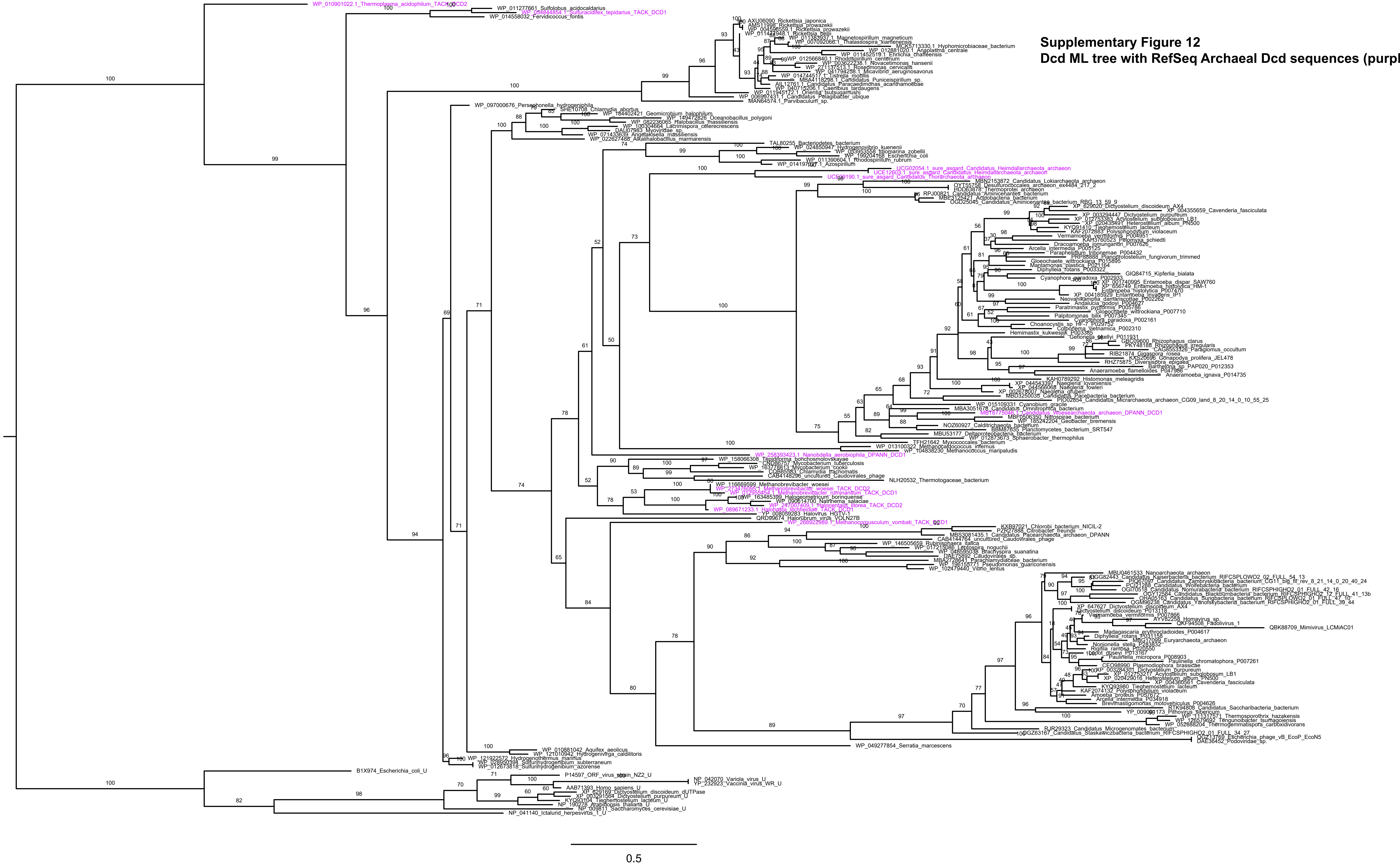

Supplementary Figure 1. Transcription levels of *dcd1$_{Dicty}$*, *dcd2$_{Dicty}$*, *thyX$_{Dicty}$* and four dCMP deaminase genes during the transition from vegetative growth (hour 0) through development (0-24 h). The gene names are followed by their Dictybase gene IDs. a. Transcription heatmap showing relative expression levels (yellow is high, blue is low, white is medium). b. The $\log_{10}$ RPKM of the same transcriptomic data shows more detailed expression levels. (RPKM is Reads Per Kilobase of transcript, per Million mapped reads.) During fruiting body development, expression of *dcd1$_{Dicty}$*, *thyX$_{Dicty}$* and *dCMP deaminase 3* increases and peaks midway, and then modestly decreases. The expression of dCMP deaminase 4 remains relatively low until 20 h and then increases. Expression of *dcd2$_{Dicty}$*, dCMP deaminases 1 and 2 is relatively high at hour 0 (transition from vegetative growth to development) and decreases through development. The heatmap and line graph were generated using data from DictyExpress (Parikh et al. 2010; Basu et al. 2013; Stajdohar et al. 2017) and agreed with other transcriptomic studies (Rosengarten et al. 2015; Santhanam et al. 2015; Katoh-Kurasawa et al. 2021). The heatmap was generated using the Pearson distance measurement method (Babicki et al. 2016).

Supplementary Figure 2. Expression of His-tagged Dcd1$_{Dicty}$ and Dcd2$_{Dicty}$ in *E. coli* and *S. pombe*. A mouse monoclonal antibody specific for His$_6$ was used to probe blots of whole cell lysates from (a) transformed Δ*dcd E. coli* strains grown either with a warm start or a cold start and (b) lysates from transformed Δ*dCMP deaminase S. pombe* cells grown in the absence or presence of 2 mM hydroxyurea (HU). The expressed proteins migrated to positions corresponding closely to the predicted full-length sizes of 21.5 kDa for Dcd1$_{Dicty}$-His$_6$ and 31.2 kDa for Dcd2$_{Dicty}$-His$_6$. Lysates from the respective knockout cells transformed with the empty vector (pQE-60 for *E. coli* and pREP3X for *S. pombe*) are labeled EV.

Supplementary Figure 3. The lack of dCMP deaminase hinders the growth of *S. pombe.* a. Growth of wild type (WT) *S. pombe* and the dCMP deaminase knockout strain (Δ*S. pombe*). b. Calculated doubling times (means of three replicates; numbers inside bars). The difference in doubling times is significant (***; $p \leq 0.001$). Error bars represent standard errors (In Panel a, solid symbols obscure small error bars).

Supplementary Figure 4 The presence of Dcd1, Dcd2, and ThyX in eukaryotic species. a. When present, Dcd1, Dcd2, and ThyX are labeled immediately after the species name in a cladogram of eukaryotes. The taxa representing the systematic relationship of 196 eukaryotic species are from The Comparative Set (TCS) of EukProt (Richter et al. 2022). After screening for contaminants, excluded proteins are labeled “deleted”. b. The presence of Dcd1, Dcd2, and ThyX are indicated in a simplified 34 taxa tree (color-coded in the 196 species tree) derived from the TCS tree. A protein marked present means some species (but not necessarily all) in the group possess it.

Supplementary Figure 5. Phylogenetic maximum likelihood tree of 235 dCTP deaminase protein sequences from diverse species. The tree is split into three parts (a, b, and c) to allow space for labeling of taxa. Scale bars (substitutions per site) for the tree are shown at bottom right. Taxon names include a GenBank Accession Number or Protein ID, and a species name. The taxa with species names followed with a U are dUTPases (part c). The included 11 dUTPase sequences form a monophyletic clade, which is used to root the tree, and the remaining sequences are dCTP deaminases. Suspected bacterial contaminants (Supplementary Table 1a), initially annotated as eukaryotic Dcds, were retained in this tree but eventually were excluded from our reduced data set (Figure 5a). Taxon names are color-coded according to biological classification: Bacteria, black; Eukaryotes, blue; Archaea, purple; Viruses, orange. Bootstrap values ≥ 50% from 1000 replicates are shown above branches.

Supplementary Figure 6. Detailed views of the Dcd1 (a) and Dcd2 (b) eukaryotic clades from the dCTP deaminase (179 taxa) ML Tree, and of the ThyX1 and ThyX2 (c) eukaryotic clades from the ThyX (116 taxa) ML tree. The major eukaryotic group is labeled after the species name. The group name before “/” is from the categories in the EukProt TCS phylogeny (Richter et al. 2022). The group name after “/” is consistent with four recent comprehensive eukaryotic phylogenetic trees (Brown et al. 2018; Burki et al. 2020; Strassert et al. 2021; Schön et al. 2021). The red oval indicates the only Blastp-identified Dcd1, Dcd2, or ThyX sequence within the major group. M means the sequences in the same major group do not form a monophyletic clade. All the species with less strong support (either has an oval or M) are written in smaller font sizes.

Supplementary Figure 7. Phylogenetic maximum likelihood tree of 174 thymidylate synthase protein sequences from diverse species. The tree is split into two parts (a and b) to allow space for labeling of taxa. Scale bars (substitutions per site) are shown at bottom right. Taxon names include a GenBank Accession Number or Protein ID, and a species name. Midpoint rooting was used because there are no closely related proteins that could be used to root the tree. Suspected bacterial contaminants, initially annotated as eukaryotic ThyX (Supplementary Table 1b), were retained in this tree but eventually were excluded from our reduced data set (Figure 5c). Taxon names are color-coded according to biological classification: Bacteria, black; Eukaryotes, blue; Archaea, purple; Viruses, orange. Bootstrap values ≥ 50% from 1000 replicates are shown above the branch.

Supplementary Figure 8. Alignment of *dcd1*, *dcd2*, and *thyX* exon sequences to identify homologous introns. The locations of introns absent from the genes are marked by dashes. The presence of introns (sequences not shown) and their lengths are indicated by highlighted numbers of base pairs. Abbreviated species names are shown on the left. Gene Accession Numbers can be found in Supplementary Table 2. Figure 7b is a graphical representation of the intron positions.

Supplementary Figure 9. GC content of eukaryotic genomes, eukaryotic clades and prokaryotic or viral sister clades of *dcd1*, *dcd2* and *thyX.* The %GC of the three genes are slightly higher than the GC content of the eukaryotic genomes (data from Supplementary Table 2). Since the average %GC of exons is not available for every species, the genome %GC is used for comparison. The overall 22% GC content of the *D. discoideum* genome is lower than the 27% GC of exons, due to the AT-richness of introns and intergenic regions (Eichinger et al. 2005).

Supplementary Figure 10. In cold start growth experiments, the dCTP deaminase knockout of *E. coli* (Δ*dcd E. coli*) transformed with $dcd2_{Dicty}$ grows significantly slower than Δ*dcd E. coli* transformed with $dcd1_{Dicty}$, or Empty Vector (Δ*dcd E. coli* + EV), or the WT *E. coli* transformed with EV (WT *E. coli* +EV). Although growing slower than under ideal conditions, both the Δ*dcd E. coli* + $dcd1_{Dicty}$ and the WT *E. coli* + EV still grew better than the Δ*dcd E. coli* + EV. However, the growth of the Δ*dcd E. coli* + $dcd2_{Dicty}$ was severely stunted, growing even more

poorly than the Δ*dcd E. coli* + EV, in spite of the fact that both $Dcd1_{Dicty}$ and $Dcd2_{Dicty}$ are expressed as intact proteins (Supplementary Figure 2). **Inset:** The calculated doubling times were 1.37, 2.5, and 2.06 h for the Δ*dcd E. coli* cells transformed with $Dcd1_{Dicty}$, $Dcd2_{Dicty}$, and EV, respectively; and 1.20 h for WT + EV. $p \leq 0.05$ (*) indicates a significant difference; $p \leq 0.01$ (**) and $p \leq 0.001$ (***) indicate highly significant differences. Data are the means of three replicates, and error bars represent standard errors. These finding suggest that under nonideal conditions, $dcd2_{Dicty}$ is less compatible than $dcd1_{Dicty}$ with *E. coli* cells.

Supplementary Figure 11. Constraints and their corresponding fully resolved trees used to test alternative topologies for Dcd (Part 1) and ThyX (Part 2). (a) The starting constraint fed into IQ-Tree. (b) The fully resolved tree determined by IQ-Tree that enforced the constraint.

Supplementary Figure 12. ML tree that includes all Dcd sequences (from Figure 5a) and NCBI RefSeq sequences of Archaeal Dcd (in purple) used to identify the archaeal clade. Instead of forming a monophyletic clade, the archaeal sequences nested within bacterial clades, indicating HGT between bacteria and archaea.

Note: Some archaeal samples from soil or ocean soil have sequences present in the Dcd tree, and in some cases group together with Dcd eukaryotic homologs with strong and moderate support. For Dcd1, the archaeal homologs belong to the TACK, Euryarchaeota, and Asgard groups. For Dcd2, the archaeal homologs belong to the DPANN and Euryarchaeota groups. The archaeal Dcd sequences do not form a single monophyletic clade that is a sister group with the eukaryotic Dcd, supporting the argument that Dcd did not exist in the LECA. Instead, the archaeal Dcd sequences are distributed widely, usually nested within bacterial clades (Figure 5a). To confirm this pattern, Dcd1 or Dcd2 sequences were used as bait in BLASTp searches of all identified archaeal clades (Baker et al. 2020) using the RefSeq Select Protein Database. A similar BLASTp was done to search the four major archaeal categories (DPANN, Asgard, Euryarchaeota, TACK) in the Non-redundant Protein Sequences Database. To augment the 179-sequence dataset (Figure 5a), 12 representative archaeal sequences were added to build a 191-sequence ML tree. Focused on archaeal sequences, this ML tree remains wholly consistent with the phylogeny presented in Figure 5a. The added RefSeq sequences appear to be splits from bigger bacterial clades, and are scattered throughout the tree. Since the archaeal Dcd sequences do not form a monophyletic

group, the vertical gene transfer hypothesis is rejected. The location of the archaea in our phylogeny could be explained by HGT between archaea and bacteria, which is known to happen frequently, or archaea and eukaryotes. A Nanoarchaeota (DPANN) genome encodes a protein with closely related orthologs in social amoebae, claiming to be the first identification of HGT from a eukaryote (social amoebae) to archaea (Rinke et al. 2013). It is also possible that these typically short metagenome-derived contigs are incorrectly annotated as archaeal. With more complete sequencing of individual archaeal and bacterial genomes that will verify the metagenomic data, more resolved trees may be built to show clearly the relationship among the eukaryotic, archaeal and bacterial Dcd sequences.

References

Babicki S, Arndt D, Marcu A, Liang Y, Grant JR, Maciejewski A, Wishart DS. 2016. Heatmapper: Web-enabled heat mapping for all. Nucleic Acids Res. 44(W1): 147.

Baker BJ, De Anda V, Seitz KW, Dombrowski N, Santoro AE, Lloyd KG. 2020. Diversity, ecology and evolution of archaea. Nat. Microbiol. 5(7): 887-900.

Basu S, Fey P, Pandit Y, Dodson R, Kibbe WA, Chisholm RL. 2013. dictyBase 2013: Integrating multiple Dictyostelid species. Nucleic Acids Research. 41(Database issue): D676-D683.

Brown MW, Heiss AA, Kamikawa R, Inagaki Y, Yabuki A, Tice AK, Shiratori T, Ishida K, Hashimoto T, Simpson AGB, et al. 2018. Phylogenomics places orphan protistan lineages in a novel eukaryotic super-group. Genome Biol Evol. 10(2): 427-433.

Burki F, Roger AJ, Brown MW, Simpson AGB. 2020. The new tree of eukaryotes. Trends Ecol Evol. 35(1): 43-55.

Eichinger L, Pachebat JA, Glöckner G, Rajandream M-, Sucgang R, Berriman M, Song J, Olsen R, Szafranski K, Xu Q, et al. 2005. The genome of the social amoeba *Dictyostelium discoideum*. Nature. 435(7038): 43-57.

Katoh-Kurasawa M, Hrovatin K, Hirose S, Webb A, Ho NI, Zupan B, Shaulsky G. 2021. Transcriptional milestones in *Dictyostelium* development. Genome Res. 31(8): 1498-1511.

Parikh A, Miranda ER, Katoh-Kurasawa M, Fuller D, Rot G, Zagar L, Curk T, Sucgang R, Chen R, Zupan B, et al. 2010. Conserved developmental transcriptomes in evolutionarily divergent species. Genome Biol. 11(3): R35.

Richter D, Berney C, Strassert J, Yu-Ping P, Herman E, Muñoz-Gómez S, Wideman J, Burki F, De Vargas C. 2022. EukProt: A database of genome-scale predicted proteins across the diversity of eukaryotes. Peer Community J, 2, e56.

Rinke C, Schwientek P, Sczyrba A, Ivanova NN, Anderson IJ, Cheng J, Darling A, Malfatti S, Swan BK, Gies EA, et al. 2013. Insights into the phylogeny and coding potential of microbial dark matter. Nature. 499(7459): 431-437.

Rosengarten RD, Santhanam B, Fuller D, Katoh-Kurasawa M, Loomis WF, Zupan B, Shaulsky G. 2015. Leaps and lulls in the developmental transcriptome of *Dictyostelium discoideum*. BMC Genomics. 16(1): 294.

Santhanam B, Cai H, Devreotes PN, Shaulsky G, Katoh-Kurasawa M. 2015. The GATA transcription factor GtaC regulates early developmental gene expression dynamics in *Dictyostelium*. Nat Commun. 6(1): 7551.

Schön ME, Zlatogursky VV, Singh RP, Poirier C, Wilken S, Mathur V, Strassert JFH, Pinhassi J, Worden AZ, Keeling PJ, et al. 2021. Single cell genomics reveals plastid-lacking picozoa are close relatives of red algae. Nature Communications. 12(1): 6651.

Stajdohar M, Rosengarten RD, Kokosar J, Jeran L, Blenkus D, Shaulsky G, Zupan B. 2017. dictyExpress: A web-based platform for sequence data management and analytics in *Dictyostelium* and beyond. BMC Bioinform. 18(1): 291.

Strassert JFH, Irisarri I, Williams TA, Burki F. 2021. A molecular timescale for eukaryote evolution with implications for the origin of red algal-derived plastids. Nature Communications. 12(1): 1879.
