# Supplementary material for: Functional Prokaryotic-Like Deoxycytidine Triphosphate Deaminases and Thymidylate Synthase in Eukaryotic Social Amoebae: Vertical, Endosymbiotic, or Horizontal Gene Transfer?
Source: Mol Biol Evol. 2023 Dec 8;40(12):msad268. doi: 10.1093/molbev/msad268 (PMC10733785; doi:10.1093/molbev/msad268)
Supplement: msad268_Supplementary_Data [file msad268_supplementary_data.zip › Supplementary material and methods MBEfinal .pdf]

Materials and Methods

Homology searches and eukaryotic contaminant analyses

The $Dcd1_{Dicty}$ (protein ID DDB0230112), $Dcd2_{Dicty}$ (protein ID DDB0230132), and $ThyX_{Dicty}$ (protein ID DDB0214905) sequences were acquired from dictyBase.org (Fey et al. 2013). (In dictyBase, the gene DDB_G0280045 is labeled incorrectly as *thyA*; it encodes a thymidylate synthase that aligns well with bacterial ThyX sequences and is described as using the $FADH_2$ cofactor required by ThyX). The protein sequences were used as BLASTp queries to search against non-redundant protein sequences (ncbi.nlm.nih.gov/BLAST), to find closely related homologs. To make sure the sequences to build trees include all the representative prokaryotic clades, the BLAST search was repeated using the Organism Search function to limit the results to sequences from particular lineages. The dCTP deaminase examples were selected from clades illustrated in Fig. 2 of Kerepesi et al. (Kerepesi et al. 2016). The prokaryotic ThyX examples were chosen from clades according to Fig. 1 of Stern et al. (Stern et al. 2010). To select alpha-proteobacteria representatives, we identified the alpha-proteobacteria species used in Figure 3 from Martijn at al. (2018), found the contig ID from their Supplementary Table 8, and searched the contigs for the Dcd and/or ThyX sequences from each species. The identified sequences are used in the 179-sequence-dataset for Dcd, and the 116-sequence-dataset for ThyX. Some alpha-proteobacteria use ThyA, and some use ThyX (Stern et al. 2010). Since ThyA sequences do not align with ThyX sequences, only alpha-proteobacteria with ThyX are included in the ThyX tree (Figure 5c). In addition to the eukaryotic sequences found by BLASTp, more eukaryotic homologs (primarily transcriptome sequences) were identified in The Comparative Set (TCS) of EukProt v3 (196 species chosen based on BUSCO completion and phylogenetic importance) (Richter et al. 2022).

Selection of eukaryotic homologs for inclusion in phylogenetic analyses required screening them for contaminating sequences (Supplementary Table 1). Those with high protein sequence identity to bacterial sequences (>50%) were included in the phylogeny analyses only if BLASTn analyses verified that the five immediate upstream and downstream (i.e., neighboring) coding gene sequences in the corresponding contigs had significant matches with eukaryotic genes. In cases where the homolog was located at one end of a contig, the immediately adjacent ten coding

sequences were examined. With short contigs, less than ten genes could be evaluated. Dcd and ThyX homologs with neighboring genes primarily bacterial in origin were classified as likely contaminants. Also flagged as likely contaminants were those Dcd and ThyX homologs that are highly similar to bacterial sequences (>95% coding sequence identity; often known endosymbionts such as *Wolbachia* in arthropods or cyanobacteria in *Paulinella*).

Many of the sequences extracted from EukProt are from transcriptome sequencing, so these cannot be screened by contig analysis. To remove potential contaminants, two strategies were used. If one species from the TCS is detected to have a Dcd_{Dicty} or $ThyX_{Dicty}$ homolog, then we go to the entire dataset setting (993 species) and BLAST the entire group that includes this putative homolog. For example, a ThyX was detected in *Klebsormidium nitens*, however, none of the other 117 Chloroplastida (*Klebsormidium nitens* belongs to this group) has ThyX. The absence of other homologs makes the *Klebsormidium nitens* sequence a likely contaminant (or a very recent HGT), and thus excludes it from the tree. In the second strategy, trial phylogenetic trees were built to detect single species that nested solely with prokaryotes, which made them likely bacterial contaminants. All the eukaryotic species possessing Dcd1, Dcd2, and ThyX proteins were labeled in Supplementary Figure 4a. The excluded species accordingly were labeled Dcd1 deleted, or Dcd2 deleted, or ThyX deleted.

Phylogenetic analyses

Collectively, the Dcd1 and Dcd2 BLASTp searches identified >600 homologs. As an outgroup for phylogenetic analyses, this data set was augmented with eleven trimeric dUTPases (members of the same superfamily as Dcd) from human, bacterial, viral, plant, and unicellular eukaryotic species. The sequences were aligned with MUSCLE (Edgar 2004). After alignment, a neighbor joining (NJ) phylogeny was constructed using the IQ-TREE webserver (Nguyen et al. 2014; Hoang, D. T. et al. 2018) (data not shown). The NJ tree was examined to guide the elimination of redundant species and oversampled lineages (lineages with very short branch lengths that show that the taxa sequences are similar), resulting in a reduced data set of 235 sequences (and retaining the tree structure) that were used to build the ML tree in Supplementary Figure 5.

The ML tree was used to guide the further removal of redundant species and oversampled lineages to yield a more extensively reduced data set of 125 out of the previous 235 sequences.

In addition, 20 representative alpha-proteobacteria based on a published phylogenetic tree (Martijn et al. 2018), and 34 Dcd homologs from EukProt (taxa with an ID starting with P0) were added to augment these lineages in the dataset. The final dataset includes 179 sequences used to build the final ML tree (Figure 5a). To confirm their correct annotation, Dcd sequences were aligned and then inspected for the presence of conserved active site residues. The active site of *E. coli* Dcd contains residues S, R, A, and E (Johansson et al. 2005). For these four active site residues, Serine (S) is present in all homologs except for *Paulinella chromatophora* (EukProt ID: P007261; replaced by N). Arginine (R) is present in all homologs. Alanine (A) is present in the majority with these exceptions: *Cavenderia fasciculata* (XP 004355659; replaced by T), *Paulinella chromatophora* (EukProt ID: P007261 replaced by S), *Anaeramoeba ignava* (EukProt ID: P014735; replaced by S), 20 bacteria and two archaea (replaced by P), two viruses and one Archaea (replaced by S). Glutamic acid (E) is present in all except *Amoeba proteus* (EukProt ID: P057672 appears to be missing C terminal residues), three bacteria (replaced by Q), and one bacterium (replaced by S). Two EukProt sequences attributed to *Gloeochaete wittrockiana* are included in the tree and group together with the Dcd1 eukaryotic clade. One of these sequences is missing more than 80 residues from the C terminus, which contains all the active site residues (EukProt ID: P015895). The second sequence is longer but still incomplete, missing the portion that would include the A and E residues (EukProt ID: P007710). Similarly, the sequence for *Hemimastix kukwesjijk* lacks the portion that would contain the A and E residues (EukProt ID: P003385).

The ThyX BLASTp search identified 174 sequences, which were used to build the initial ML tree (Supplementary Figure 7). As done with the Dcd sequences, this ThyX data set was reduced to 71 sequences, and four more categories of sequences were added to make a 116-sequence dataset. These included 21 alpha-proteobacteria ThyX sequences based on a published phylogenetic tree of alpha-proteobacteria (Martijn et al. 2018). Secondly, $ThyX_{Dicty}$ was used as bait to search EukProt (TCS setting), and a homolog from *Spironema* was added. Thirdly, a BLASTp using ThyX from *Tritrichomonas* found 21 homologs, mostly from Bacillota (Firmicutes), and included some archaeal, and viral sequences. Finally, we included two *Borrelia* bacteria ThyX homologs used to perform *E. coli* complementation experiments (Zhong et al. 2006). To confirm that the proteins were correctly annotated, the ThyX alignments were inspected for the presence of conserved active site residues. The active site of the *Helicobacter*

pylori ThyX contains the residues H, R, S and Y (Leduc et al. 2004). In the alignment of 116 ThyX homologs, active site residues are conserved with several exceptions. For Histidine (H), the exceptions are one bacterium and two archaea (replaced by D), one phage (replaced by I), and two archaea (replaced by Y). For Arginine (R), they are two archaea (replaced by E), two bacteria (replaced by L), one bacterium (replaced by N), three bacteria (replaced by H), four bacteria (replaced by T), and one bacterium (replaced by K). For the Serine (S), it is one phage (replaced by T). For the Tyrosine (Y), they are one virus (replaced by A), two archaea and one bacterium (replaced by F). All the sequences were pooled to make a 116-sample data set used to build the final ML tree (Figure 5c).

Each of these large and small datasets for Dcd (235 and 179 samples) and ThyX (174 and 116 samples) were aligned with MUSCLE and trimmed using trimAl 1.3 (Capella-Gutiérrez et al. 2009) with the Gappyout option on the Phylemon 2 webserver (Sánchez, R. et al. 2011). Phylogenetic analysis models were identified (IQ-TREE 1.6.12 ModelFinder) (Kalyaanamoorthy et al. 2017). ML trees were constructed using the model LG+G4, ultrafast bootstrap (1000 replicates) (Nguyen et al. 2014; Hoang, Diep Thi et al. 2018). For the small data sets, ML trees were built ten times each for Dcd and ThyX, to fully explore the tree space. Dcd trees were rooted on the dUTPase sequences, whereas the ThyX trees were rooted using midpoint rooting because there are no proteins closely related to ThyX that could serve as an outgroup. Trees with the highest likelihoods are presented in Figure 5.

To test for alternative hypotheses regarding the evolutionary origins of the eukaryotic Dcd and ThyX sequences, the Dcd and ThyX ML trees were each compared to trees that represent alternative topologies using the Approximately Unbiased (AU) topology (Shimodaira 2002) implemented by IQ-TREE. For these analyses, we used the ML trees as the reference topologies to compare to the alternative topologies. One alternative topology tested whether the independent eukaryotic clades may have arisen from a single ancestral event by creating a constraint tree that forced all clades of eukaryotic sequences to be monophyletic. We also tested whether each independent eukaryotic clade in the ML trees was acquired by intracellular transfer from mitochondria. This was done by creating a constraint tree that forced each independent eukaryotic clade to be monophyletic with alpha-proteobacteria, the presumed progenitor lineage for mitochondria. In alpha-proteobacteria, the Dcd and ThyX homologs each form two

monophyletic groups. For both, one group is clearly the main ancestral clade which includes many alpha-proteobacteria species, while the other group only contains two taxa (and thus likely derive from HGT or misannotation), so the main ancestral alpha-proteobacteria clade is used as the representative of ancestral mitochondria for topology tests. The origin of mitochondria remains uncertain, because either all or a subgroup of alpha-proteobacteria may share the ancestor with mitochondria. If the eukaryotic Dcd1, Dcd2 or ThyX is mitochondrial in origin, then its clade should be either a sister group to all alpha-proteobacteria or it will nest within alpha-proteobacteria and be a sister group with one specific clade of alpha-proteobacteria. To maintain both possibilities, when the alpha-proteobacteria were included in the topology tests (Constraints 2 and 3), the eukaryotic clade was allowed to possibly nest within the alpha-proteobacteria. Finally, we tested whether all eukaryotic clades may have been acquired from mitochondria by creating a tree that forced all eukaryotic homologs to be monophyletic with alpha-proteobacteria (Constraint 4). We generated minimal constraint ML trees in IQ-TREE that forced together the corresponding clades and allowed the rest of the tree to be freely and fully resolved (Supplementary Figure 11). We then compared the unconstrained ML tree to the constraint trees using the AU test in IQ-TREE.

GC content, codon usage, and intron content of Dcd and ThyX homologs

The GC content of $dcd1_{Dicty}$, $dcd2_{Dicty}$, *thyX,* and the corresponding genes of their eukaryotic, sister clade prokaryotic and viral homologs were analyzed using GC Content Calculator (2022) (Supplementary Table 2). In addition, the % GC of *D. discoideum* native genes *act5*, *act8*, *tubA2*, *dut*, and *dCMP deaminases 1 - 4* were calculated. The actin genes are selected because these genes are conserved among eukaryotes and actin is abundant in social amoebae; the dCMP deaminases are the genes that belong to dTTP *de novo* synthesis pathway, and our phylogenetic analyses show that all four copies of dCMP deaminases in social amoebae form a monophyletic group with other eukaryotic dCMP deaminases, confirming their eukaryotic native origin (data not shown).

The codon usage of the *D. discoideum* genome was retrieved from the Codon Usage Database (Nakamura et al. 2000). The coding sequences of $dcd1_{Dicty}$ (DDB_G0293580) (XM_629018.1), $dcd2_{Dicty}$ (DDB_G0268194) (XM_642535.1), *thyX* (DDB_G0280045) (XM_636243.1) and

native genes *act5* (DDB_G0289663) (XM_630996.1), *act8* (DDB_G0269234) (XM_641596.1), *dut* (DDB_G0293374) (XM_629167.1), *tubA2* (DDB_G0281889) (XM_635391.1), and *dCMP deaminases 1 - 4* (DDB_G0282255, DDB_G0271914, DDB_G0286161, DDB_G0288019) were retrieved from NCBI and dictyBase. The *S. pombe* dCMP deaminase (SPBC2G2.13c) (NM_001022361.2) was retrieved from NCBI and PomBase (Harris et al. 2022). The *E. coli* dCTP deaminase sequence (ECK2059) was retrieved from NCBI. Codon usage was analyzed using the Sequence Manipulation Suite Codon Usage Version 2 software (Stothard 2000) (Supplementary Table 3). Many of the sequences in the EukProt database are from transcriptomes, so genome and intron information are unavailable, resulting in the exclusion of the sequences from the GC content, codon usage, and intron comparisons.

To identify homologous introns, the protein translations of exon sequences of eukaryotic *dcd* and *thyX* homologs first were aligned on BioEdit using MUSCLE (Hall 1999). Then the gene sequences (with exons and introns) were manually aligned to the exon-translated alignment to identify intron positions and determine the intron sizes for each species. Intron splice junctions, starting with a 5′ GT and ending with a 3′ AG, were confirmed for all but one intron, which carried the minor junction variant of a 5′ GC. Annotation errors at the 5′ and/or 3′ ends were apparent for the *dcd* sequences of *Planoprotostelium* and *Paraglomus* because their protein sequences failed to align with other homologs. These errors were manually corrected by using a BLAST search with *dcd* homologs to identify the correct sequence of the 5′ and/or 3′ exon (Supplementary Figure 8).

E. coli expression plasmids

The *E. coli* expression plasmids used are listed in Supplementary Table 4. Sequences of the constructs are provided in Supplementary Table 5. Expression plasmids containing either $dcd1_{Dicty}$ or $dcd2_{Dicty}$ were constructed by GenScript (Piscataway, NJ) using $dcd1_{Dicty}$ or $dcd2_{Dicty}$ coding sequences, adding the sequence $(GGA)_5$ followed by the sequence coding for LVPR to generate a thrombin cleavage site, to the 3’-terminus. The dinucleotide GA was added after the NcoI site (CCATGG) in the 5’ end, resulting in a glycine after the start methionine. The synthesized products were then inserted into pQE-60 between the NcoI and BamHI sites. The expressed proteins are predicted dCTP deaminases with a C-terminus tail sequence of

GGGGGLVPRGSRSHHHHHH. The $(Gly)_5$ was added to spatially separate the $(His)_6$ from the C-terminus of the deaminase polypeptide and the LVPRGS is the thrombin cleavage site. The coding sequence for GSRSHHHHHH was present in the vector pQE-60 (Qiagen, Germantown, MD) which is a low copy number plasmid with a T5 promoter and ampicillin resistance gene. The pQE-60-$thyX_{Dicty}$ was synthesized by GenScript where LVPR was added directly without $(Gly)_5$ to the C-terminus of $ThyX_{Dicty}$.

<u>*E. coli* competent cells and transformation</u>

E. coli strains used in this study are listed in Supplementary Table 6. WT BW25113 and Δ*dcd E. coli* JW2050-1 are from The Coli Genetic Stock Center (Yale University, New Haven, CT) (Baba et al. 2006). WT χ2842 and Δ*thyA E. coli* χ2913 are from The Coli Genetic Stock Center and Roy Curtiss Lab (Univ. Florida) (Curtiss et al. 1968). Following a vendor protocol (Gene Pulser Xcell Electroporation System Instruction Manual; Bio-Rad, Hercules, CA), electrocompetent cells were made by growing the corresponding *E. coli* strain (Δ*dcd E. coli*, Δ*thyA E. coli* or WT) in Luria-Bertani media (0.5% yeast extract, 1% tryptone, 1% sodium chloride, pH 7) to mid-log phase at 37 °C. The cells were chilled on ice and then collected by centrifugation. The supernatant was discarded, and after two washes with 10% glycerol, cells were resuspended to 1-3 x 10^{10} cells/ml in 10% glycerol. Competent cells (50 μl) were mixed with 10 ng of plasmid DNA and transformed by electroporation using 2 mm gap electroporation cuvettes.

<u>*E. coli* growth and expression analyses</u>

To test the function of *D. discoideum* dCTP deaminases in Δ*dcd E. coli,* cultures were always started with a single *E. coli* colony inoculated into 3 ml MOPS minimal media with 0.25% glucose (Teknova, Hollister, CA) (Neidhardt et al. 1974) with antibiotics as needed (30 μg/ml kanamycin, or 50 μg/ml ampicillin). For Δ*dcd E. coli* which has a *kan* insertion, the antibiotic was present during transformations along with ampicillin to ensure selection. For growth studies that included WT *E. coli,* kanamycin was omitted but ampicillin was present to maintain plasmids. Mini-cultures (3 ml) were shaken continuously at 225 rpm at 37 °C and growth was monitored by measuring the OD_{600}. When the OD_{600} was in the range of 0.25 to 0.8, cells were inoculated into 12 ml of media to obtain a final starting OD_{600} of 0.06. For warm start growth

experiments, 12 ml of media was warmed to 37 °C and shaken for 2 h before inoculation. For cold start growth experiments, 12 ml of media was cooled to 4 °C without pre-aeration before inoculation. All 12 ml cultures then were shaken continuously at 225 rpm at 37 °C. Cell growth was monitored using OD_{600} readings for 7 to 10 hours depending on the study. At high densities, to ensure accurate readings, samples were diluted to under $OD_{600} = 0.3$, and the final OD_{600} was calculated.

To test the function of the *D. discoideum* ThyX in the Δ*thyA E. coli,* mini-cultures (MOPS minimal media) included 25 µg/ml thymine (ThermoFisher Scientific, NJ) to insure the growth of the Δ*thyA E. coli* and Δ*thyA E. coli* + EV strains. Cells from growing mini-cultures were collected by centrifugation at room temperature (1 min at 13,000 rpm), resuspended in 0.5 ml of media without thymine and used to inoculate 12 ml of media lacking thymine for warm start growth studies (Neidhardt et al. 1974). Cultures were monitored as described above.

<u>*E. coli* doubling time calculation</u>

OD_{600} readings were converted to cell numbers using an OD_{600} of 0.1 to correspond to 2×10^7 cells/ml (Sezonov et al. 2007). The $\log_{10}$ of cell numbers was plotted against time and from the steepest linear portion of the curve, the exponential growth interval was identified. The *E. coli* cell doubling constant µ was calculated using the formula:

$\mu = ((\log_{10} N - \log_{10} N_0)\ 2.303) / (t - t_0)$. The doubling time was calculated using the formula: doubling time = $\ln 2/ \mu$**.** A minimum of three replicates was used to calculate the mean doubling time for each strain. The data were tested by single factor ANOVA (analysis of variance); $p < 0.05$ is considered significant.

<u>*S. pombe* expression plasmids</u>

The *S. pombe* expression plasmids used are listed in Supplementary Table 4. Sequences of the constructs are provided in Supplementary Table 5 and PCR primers are listed in Supplementary Table 7. The *S. pombe* $dcd1_{Dicty}$ expression plasmid was built by digesting the fission yeast pREP3X vector (Forsburg 1993) (a gift from Dr. R. Wilson, Univ. Nebraska-Lincoln) with XhoI and SmaI. The coding sequence for *dcd1-His*$_6$ was amplified by PCR using pQE-60-*dcd1-His*$_6$ as a template. The amplicon was inserted between the XhoI and SmaI sites, downstream of the *nmt*

promoter and upstream of the *nmt* terminator (GenScript). The $dcd2_{Dicty}$ expression vector was constructed by first digesting pREP3X-$dcd1_{Dicty}$ with XhoI and SmaI. The pREP3X backbone was isolated by agarose gel electrophoresis and recovered by electroelution (Sambrook and Russell 2001). The $dcd2_{Dicty}$ was amplified using pQE-60-$dcd2_{Dicty}$ as the template and primers with 5' SalI and 3' SmaI sites (Supplementary Table 7). The PCR product was digested with SalI and SmaI, purified and ligated with linearized pREP3X using T4 DNA Ligase (ThermoFisher Scientific). Ligation products were transformed into XL1-Blue Competent Cells (Agilent, Santa Clara, CA) and the plasmid was purified from transformed cells using a QIAprep Spin Miniprep Kit (Qiagen). Preparative quantities were prepared with a Plasmid Midi Kit (Qiagen). Constructs were verified by sequencing (Eurofins Scientific, Des Moines, IA).

S. pombe competent cells and transformation

S. pombe strains used in this study are listed in Supplementary Table 6. The WT *S. pombe* and Δ*dCMP deaminase S. pombe* are from Dr. P. Russell (Scripps Research, La Jolla, CA) (Sánchez et al. 2012). Following a standard transformation protocol (Suga and Hatakeyama 2005), *S. pombe* cells were grown in YES medium (BioWORLD, Dublin, OH) to late log phase, diluted ten-fold and shaken at 30 °C in SLD (0.67% Bacto yeast nitrogen base, 0.5% glucose) supplemented with amino acids (DO Supplement (-Leu); Takara, San Jose, CA) and 225 mg/l L-Leucine (Sigma, St. Louis, MO) to a density of 1×10^7 cells/ml. The cells were washed with sterile cold deionized water and resuspended to a density of 1×10^9 cells/ml in 30% ice-cold glycerol containing 0.1 M lithium acetate, pH 4.9. Aliquots of 50 µl/tube were left for 30 min, at room temperature, and then stored at -70 °C until the day of transformation.

S. pombe cells were thawed at 40 °C for 2 min and mixed with 5 µl salmon sperm DNA (10 mg/ml; ThermoFisher) and 5 -10 ng of either pREP3X-$dcd1_{Dicty}$ or pREP3X-$dcd2_{Dicty}$. Polyethylene glycol 4000 (50% w/v; 145 µl) was added, and the mixture was heat-shocked at 43 °C for 15 min. The cell suspension was diluted with 10 mM Tris, 1 mM EDTA, pH 7.5 and spread onto EMM2 selection plates (Sunrise Science Products, Knoxville, TN) with DO Supplement (-Leu) incubated at 30 °C. Colonies of transformants appeared after 3-5 days.

PCR of *S. pombe* transformants

Using sterile pipette tips, *S. pombe* colonies were picked and put into 1.5 ml microcentrifuge tubes with 10 µl of sterile deionized water. The tubes were put at -20 °C for 2 min, vortexed for 4 sec at room temperature and then boiled in a water bath for 2 min. This sequence was repeated two more times, and 2 µl were used as the DNA template in PCR analyses with primers corresponding to the pREP3X sequences flanking the insert (Supplementary Table 7). Sizes of PCR products confirmed the presence of the plasmid.

S. pombe growth analyses

Seed cultures were grown in shaken suspension (225 rpm, 30 °C) in EMM2 with amino acids (DO Supplement, -Leu) until mid-log phase. For growth in the presence of hydroxyurea (HU; ThermoFisher), cells were inoculated into 20 ml of pre-warmed media at 30 °C containing 2 mM HU (Sánchez et al. 2012) to a final OD_{600} of 0.1, and shaken at 225 rpm. Cell growth was monitored by taking aliquots (0.5 ml) that were diluted as needed to obtain OD_{600} readings below 0.3 that were then multiplied by the dilution factor to obtain final OD_{600} values. An OD_{600} = 0.1 is approximately 2 x 10^6 cells/ml (Kawai et al. 2010).

S. pombe doubling time analyses

The *S. pombe* doubling times were calculated with the same formula used with the *E. coli* data. The $\log_{10}$ of *S. pombe* cell numbers was plotted against time and from the steepest linear portion of the curve, the exponential growth interval was identified. Another method used the formula: doubling time = log (2^{t2-t1})/ log (y/x); where y = cells/ml at time t2, x = cells/ml at time t1, which yielded closely matching times (Paul Nurse 2014). The mean doubling time was calculated from three to seven replicates per strain. ANOVA single factor statistical analyses were used to test the significance of difference between strain doubling times. We determined that the WT *S. pombe* and Δ*S. pombe* strains have doubling times of 3.3 and 5.9 hours, respectively (Supplementary Figure 3). Validating our growth conditions and calculation methods, the WT number is consistent with previously published doubling times of *S. pombe* in EMM2 (Petersen and Russell 2016), and for Δ*S. pombe*, the longer doubling time is consistent with its slower growth shown by published plate growth data (Sánchez et al. 2012).

Lysates of *E. coli* and *S. pombe* cells, SDS-PAGE and Immunoblotting

E. coli cells grown to middle to late log phase (OD_{600} 0.5-1.2;1 ml) were pelleted by centrifugation in a microfuge at room temperature (5 min. at top speed). Cells were lysed with 100 µl of 10% SDS (sodium dodecyl sulfate) and boiled for 2 min. Stationary phase *S. pombe* cells (15 ml) were collected by centrifugation at 4 °C. Cell pellets were resuspended in 0.6 ml of sterile water before the addition of 0.6 ml of 0.6 N NaOH, and left for 10 min at room temperature. One ml of the suspension was spun for 1 min at top speed in a microfuge and the supernatant discarded (Matsuo et al. 2006). Sample buffer for SDS-PAGE was added to the permeabilized cells that were then boiled for 3 min.

E. coli and *S. pombe* lysates were analyzed by SDS-PAGE (10% acrylamide) using the Laemmli buffer system. To visualize the proteins, gels were stained using Coomassie blue (0.25 g/L Coomassie R-250, 50% methanol, 10% acetic acid). Protein standards (PageRuler Prestained Protein Ladder; ThermoFisher) were used to estimate the size of recombinant proteins. For immunoblotting, cell lysates were separated by SDS-PAGE, transferred to polyvinylidene difluoride (PVDF) by semi-dry blotting using a discontinuous buffer system where the anode buffer was 40 mM N-cyclohexyl-3-aminopropanesulfonic acid (CAPS), 60 mM Tris pH 9.6, 15% methanol and the cathode buffer was 40 mM CAPS, 60 mM Tris, pH 9.6, containing 0.1% SDS (Bio-Rad Bulletin 2134). Blots were blocked with 5% non-fat milk in 25 mM Tris, 150 mM NaCl, pH 7.2 (TBS), and washed with TBS containing 0.1% Tween-20 (TBST). The blot was then incubated with a mouse anti-6x-His Tag monoclonal primary antibody (ThermoFisher) in 5% bovine serum albumin. After three washes with TBST, the blot membrane was incubated with a donkey anti-mouse horse radish peroxidase conjugate (Novex/ThermoFisher Scientific). After three washes with TBST, the membrane was developed with Pierce ECL western blotting substrate (ThermoFisher). Images are captured by a C-Digit Blot Scanner (Li-Cor Image Studio Software ver. 5.2.5; LI-COR Biosciences, Lincoln, NE).

Cell imaging

Mid-log phase *E. coli* cells from thymine-supplemented mini-cultures were diluted in MOPS minimal medium without thymine to a starting OD600 of 0.06 and incubated at 37°C with shaking (225 rpm) for 3.5 hours. Cells were stained with slight modification as described (Chauhan et al. 2019). Briefly, cells were centrifuged, washed with PBS, and resuspended in 500

μl of PBS. Cell membranes were stained, at a final concentration of 4 μg/ml (ThermoFisher), with N-(3-triethylammonium-propyl)-4-(6-(4-(diethylamino)phenyl)hexatrienyl) pyridinium dibromide (FM 4-64). After cells were incubated in the dark at room temperature for 15 min, 4′,6-diamidino-2-phenylindole (DAPI) was added (final concentration 1 μ/ml) and cells were left in the dark at room temperature for an additional 15 min. After a final wash with PBS, resuspended cells were stored in the dark at 4 °C, until observed by microscopy. Cells were observed using a Nikon A1R-Ti2 inverted confocal laser scanning microscope (Microscopy Core Research Facility, Univ. Nebraska). A 60x oil objective was used with a 2x zoom for a total magnification of 1200x. DAPI was detected by the blue channel at 405 nm, FM4-64 was excited with a 488 nm laser and imaged in the 560 nm channel.

References

Baba T, Ara T, Hasegawa M, Takai Y, Okumura Y, Baba M, Datsenko KA, Tomita M, Wanner BL, Mori H. 2006. Construction of escherichia coli K-12 in-frame, single-gene knockout mutants: The keio collection. Mol Syst Biol. 2: 2006.0008.

Capella-Gutiérrez S, Silla-Martínez JM, Gabaldón T. 2009. trimAl: A tool for automated alignment trimming in large-scale phylogenetic analyses. Bioinformatics. 25(15): 1972-1973.

Chauhan D, Srivastava PA, Ritzl B, Yennamalli RM, Cava F, Priyadarshini R. 2019. Amino acid-dependent alterations in cell wall and cell morphology of *Deinococcus indicus* DR1. Front in Microbiol. 10: 1449.

Curtiss R3, Charamella LJ, Stallions DR, Mays JA. 1968. Parental functions during conjugation in *Escherichia coli* K-12. Bacteriol.Rev. 32(4 Pt 1): 320-348.

Edgar RC. 2004. MUSCLE: Multiple sequence alignment with high accuracy and high throughput. Nucleic Acids Res. 32(5): 1792-1797.

Fey P, Dodson RJ, Basu S, Chisholm RL. 2013. One stop shop for everything dictyostelium: dictyBase and the dicty stock center in 2012. In: *Dictyostelium discoideum* protocols. Eichinger L and Rivero F, editors. Totowa, NJ: Humana Press. p. 59-92.

Forsburg SL. 1993. Comparison of *Schizosaccharomyces pombe* expression systems. Nucleic Acids Res. 21(12): 2955-2956.

Hall. BioEdit: A user-friendly biological sequence alignment editor and analysis program for windows 95/98/NT. Nucleic acids symposium series, 41; 1999. [London]: Information Retrieval Ltd., c1979-c2000. p 95-98.

Harris MA, Rutherford KM, Hayles J, Lock A, Bähler J, Oliver SG, Mata J, Wood V. 2022. Fission stories: Using PomBase to understand schizosaccharomyces pombe biology. Genetics. 220(4): iyab222.

Hoang DT, Chernomor O, von Haeseler A, Minh BQ, Vinh LS. 2018. UFBoot2: Improving the ultrafast bootstrap approximation. Mol Biol Evol. 35(2): 518-522.

Johansson E, Fanø M, Bynck JH, Neuhard J, Larsen S, Sigurskjold BW, Christensen U, Willemoës M. 2005. Structures of dCTP deaminase from escherichia coli with bound substrate and product: Reaction mechanism and determinants of mono- and bifunctionality for a family of enzymes. J Biol Chem. 280(4): 3051-3059.

Kalyaanamoorthy S, Minh BQ, Wong TKF, von Haeseler A, Jermiin LS. 2017. ModelFinder: Fast model selection for accurate phylogenetic estimates. Nat. Methods. 14(6): 587-589.

Kawai S, Hashimoto W, Murata K. 2010. Transformation of *Saccharomyces cerevisiae* and other fungi: Methods and possible underlying mechanism. Bioeng Bugs. 1(6): 395-403.

Kerepesi C, Szabó JE, Papp-Kádár V, Dobay O, Szabó D, Grolmusz V, Vértessy BG. 2016. Life without dUTPase. Front Microbiol. 7: 1768.

Leduc D, Graziani S, Lipowski G, Marchand C, Le Maréchal P, Liebl U, Myllykallio H. 2004. Functional evidence for active site location of tetrameric thymidylate synthase X at the interphase of three monomers. Proc Natl Acad Sci U.S.A. 101(19): 7252-7257.

Martijn J, Vosseberg J, Guy L, Offre P, Ettema TJG. 2018. Deep mitochondrial origin outside the sampled alphaproteobacteria. Nature. 557(7703): 101-105.

Matsuo Y, Asakawa K, Toda T, Katayama S. 2006. A rapid method for protein extraction from fission yeast. Biosci Biotechnol Biochem. 70(8): 1992-1994.

Nakamura Y, Gojobori T, Ikemura T. 2000. Codon usage tabulated from international DNA sequence databases: Status for the year 2000. Nucleic Acids Res. 28(1): 292.

Neidhardt FC, Bloch PL, Smith DF. 1974. Culture medium for enterobacteria. J Bacteriol. 119(3): 736-747.

Nurse P. [Internet]. 2014. Fission Yeast Handbook. Gonbach (Germany): Baumannlab.org. [cited 2021 March 31]. Available from: https://www.baumannlab.org/documents/Nurselab_fissionyeasthandbook_000.pdf

Nguyen L, Schmidt HA, von Haeseler A, Minh BQ. 2014. IQ-TREE: A fast and effective stochastic algorithm for estimating maximum-likelihood phylogenies. Mol Biol Evol. 32(1): 268-274.

Petersen J and Russell P. 2016. Growth and the environment of *Schizosaccharomyces pombe*. Cold Spring Harb Protoc. 2016(3): pdb.top079764.

Richter D, Berney C, Strassert J, Yu-Ping P, Herman E, Muñoz-Gómez S, Wideman J, Burki F, De Vargas C. 2022. EukProt: A database of genome-scale predicted proteins across the diversity of eukaryotes. Peer Community J, 2, e56.

Sambrook J and Russell DW. 2001. Gel electrophoresis of DNA and pulsed-field agarose gel electrophoresis. In: Molecular cloning: A laboratory manual. 3rd ed. Cold Spring Harbor, New York: Cold Spring Harbor Laboratory Press. p. 5.23-5.25.

Sánchez A, Sharma S, Rozenzhak S, Roguev A, Krogan NJ, Chabes A, Russell P. 2012. Replication fork collapse and genome instability in a deoxycytidylate deaminase mutant. Mol Cell Biol. 32(21): 4445-4454.

Sánchez R, Serra F, Tárraga J, Medina I, Carbonell J, Pulido L, de María A, Capella-Gutíerrez S, Huerta-Cepas J, Gabaldón T, et al. 2011. Phylemon 2.0: A suite of web-tools for molecular evolution, phylogenetics, phylogenomics and hypotheses testing. Nucleic Acids Res. 39: W470-W474.

ScienceBuddies. 2022. Genomics %G~C Content Calculator [Internet].[cited 2022 June 12] Available from: https://www.sciencebuddies.org/science-fair-projects/references/genomics-g-c-content-calculator .

Sezonov G, Joseleau-Petit D, D'Ari R. 2007. *Escherichia coli* physiology in Luria-Bertani broth. J Bacteriol. 189(23): 8746-8749.

Shimodaira H. 2002. An approximately unbiased test of phylogenetic tree selection. Syst Biol. 51(3): 492-508.

Stern A, Mayrose I, Penn O, Shaul S, Gophna U, Pupko T. 2010. An evolutionary analysis of lateral gene transfer in thymidylate synthase enzymes. Syst Biol. 59(2): 212-225.

Stothard P. 2000. The sequence manipulation suite: JavaScript programs for analyzing and formatting protein and DNA sequences. BioTechniques. 28(6): 1102-1104.

Suga M and Hatakeyama T. 2005. A rapid and simple procedure for high-efficiency lithium acetate transformation of cryopreserved *Schizosaccharomyces pombe* cells. Yeast. 22(10): 799-804.

Zhong J, Skouloubris S, Dai Q, Myllykallio H, Barbour AG. 2006. Function and evolution of plasmid-borne genes for pyrimidine biosynthesis in borrelia spp. J. Bacteriol. 188(3): 909-918.
