# Supplementary material for: Functional Prokaryotic-Like Deoxycytidine Triphosphate Deaminases and Thymidylate Synthase in Eukaryotic Social Amoebae: Vertical, Endosymbiotic, or Horizontal Gene Transfer?
Source: Mol Biol Evol. 2023 Dec 8;40(12):msad268. doi: 10.1093/molbev/msad268 (PMC10733785; doi:10.1093/molbev/msad268)
Supplement: msad268_Supplementary_Data [file msad268_supplementary_data.zip › Supplementary Table 4, 6, 7 MBEfinal.docx]

Supplementary Table 4 Expression plasmids used in this study

| **Plasmid** | **Description** | **Source** |
| --- | --- | --- |
| pQE-60  (Empty Vector) | *E. coli* expression vector, *Amp^R^* | GenScript |
| pQE-60-*dcd1_Dicty_* | *E. coli* expression vector, containing the predicted coding sequence for a dCTP deaminase of *D. discoideum* (*dcd1_Dicty_*, gene ID: DDB_G0293580, XM_629018.1) with a C-terminal His_6;_ *Amp^R^* | GenScript |
| pQE-60-*dcd2_Dicty_* | *E. coli* expression vector, containing the predicted coding sequence for a dCTP deaminase of *D. discoideum* (*dcd2_Dicty_*, gene ID: DDB_G0268194, XM_642535.1) with a C-terminal His_6;_ *Amp^R^* | GenScript |
| pQE-60-*thyX_Dicty_* | *E. coli* expression vector, containing the predicted coding sequence for a thymidylate synthase of *D. discoideum* (*thyX_Dicty_*, gene ID: DDB_G0280045, XM_636243.1) with a C-terminal His_6;_ *Amp^R^* | GenScript |
| pREP3X  (Empty Vector) | *S. pombe* expression vector, contains a *LEU2* gene (which enables cell growth in defined media without leucine) | (Forsburg 1993) |
| pREP3X-*dcd1_Dicty_* | *S. pombe* expression vector, containing the predicted coding sequence for a dCTP deaminase of *D. discoideum* (*dcd1_Dicty_*) with a C-terminal His_6_, *LEU2* | GenScript |
| pREP3X-*dcd2_Dicty_* | *S. pombe* expression vector, containing the predicted coding sequence for a dCTP deaminase of *D. discoideum* (*dcd2_Dicty_*) with a C-terminal His_6_, *LEU2* | This study |

Supplementary Table 6 *E. coli and S. pombe s*trains used for complementation experiments

|  | **Genotype** | **Description** | **Source** |
| --- | --- | --- | --- |
| *E. coli* for *dcd_Dicty_* experiments | | | |
| WT *E. coli* | F*-, Δ(araD-araB)567, ΔlacZ4787(::rrnB-3), λ-, rph-1, Δ(rhaD-rhaB)568, hsdR514* | BW25113 (parent) | (Baba et al. 2006) |
| Δ*E. coli* | F-, *Δ(araD-araB)567, ΔlacZ4787(::rrnB-3), λ-, Δdcd-763::kan, rph-1, Δ(rhaD-rhaB)568, hsdR514* | JW2050-1  The *dcd* gene which codes for the *E. coli* dCTP deaminase is disrupted in BW25113 by a kanamycin resistance cassette | (Baba et al. 2006) |
| Δ*E. coli + dcd1_Dicty_* | F-, *Δ(araD-araB)567, ΔlacZ4787(::rrnB-3), λ-, Δdcd-763::kan, rph-1, Δ(rhaD-rhaB)568, hsdR514* | Δ*E. coli* (JW2050-1) transformed with pQE-60-*dcd1_Dicty_* | This study |
| Δ*E. coli + dcd2_Dicty_* | F-, *Δ(araD-araB)567, ΔlacZ4787(::rrnB-3), λ-, Δdcd-763::kan, rph-1, Δ(rhaD-rhaB)568, hsdR514* | Δ*E. coli* (JW2050-1) transformed with pQE-60-*dcd2_Dicty_* | This study |
| Δ*E. coli +* EV | F-, *Δ(araD-araB)567, ΔlacZ4787(::rrnB-3), λ-, Δdcd-763::kan, rph-1, Δ(rhaD-rhaB)568, hsdR514* | Δ*E. coli* (JW2050-1) transformed with pQE-60 empty vector (EV) | This study |
| WT *E. coli* + EV | F-, *Δ(araD-araB)567, ΔlacZ4787(::rrnB-3), λ-, rph-1, Δ(rhaD-rhaB)568, hsdR514* | WT *E. coli* (BW25113) transformed with pQE-60 empty vector (EV) | This study |

| Suppl. Table 6 (cont’d) | **Genotype** | **Description** | **Source** |
| --- | --- | --- | --- |
| *E. coli* for *thyX_Dicty_* experiments | | | |
| WT *E. coli* | F+, *rpoS396(Am), λ- rph-1* | *E. coli* K-12, χ2842 (W1485) (parent) | From Dr. Roy Curtiss, III, (Lederberg and Lederberg 1953) |
| Δ*E. coli* | F-*, λ-, thyA748::Tn10, rph-1?* | χ2913, the *thyA* gene which codes for the *E. coli* thymidylate synthase is disrupted in χ2842 | (Curtiss et al. 1968) |
| Δ*E. coli + thyX_Dicty_* | F-*, λ-, thyA748::Tn10, rph-1?* | Δ*E. coli* (χ2842) transformed with pQE-60-*thyX_Dicty_* | This study |
| Δ*E. coli +* EV | F-*, λ-, thyA748::Tn10, rph-1?* | Δ*E. coli* (χ2842) transformed with pQE-60 empty vector (EV) | This study |
| WT *E. coli* + EV | F+, *rpoS396(Am), λ- rph-1* | WT *E. coli* (χ2842) transformed with pQE-60 empty vector (EV) | This study |

| Suppl. Table 6 (cont’d) | **Genotype** | **Description** | **Source** |
| --- | --- | --- | --- |
| *S. pombe* for *dcd_Dicty_* experiments | | | |
| WT *S. pombe* | *h−leu1-32* | AS237  (parent) | (Sánchez et al. 2012) |
| Δ*S. pombe* | *h−leu1-32 ura4-D18 dcd1::kanMX6* | AS351  Gene SPBC2G2.13c which codes for the *S. pombe* dCMP deaminase is disrupted in AS237 with a kanamycin resistance cassette | (Sánchez et al. 2012) |
| Δ*S. pombe* + *dcd1_Dicty_* | *h−leu1-32 ura4-D18 dcd1::kanMX6* | AS351 (Δ*S. pombe*) transformed with pREP3X-*dcd1_Dicty_* | This study |
| Δ*S. pombe* + *dcd2_Dicty_* | *h−leu1-32 ura4-D18 dcd1::kanMX6* | AS351 (Δ*S. pombe*) transformed with pREP3X-*dcd2_Dicty_* | This study |
| Δ*S. pombe* + EV | *h−leu1-32 ura4-D18 dcd1::kanMX6* | AS351 (Δ*S. pombe*) transformed with pREP3X empty vector (EV) | This study |
| WT *S. pombe* + EV | *h−leu1-32* | AS237 (WT *S. pombe*) transformed with pREP3X empty vector (EV) | This study |

**Supplementary Table 7. Primers used to generate restriction sites, and to screen for inserts of pREP3X-*dcd2_Dicty_* constructs.**

| **Primer name** | **Orientation** | **Sequence (5’ to 3’)** | **Target** |
| --- | --- | --- | --- |
| dcd2_For1_SaII | Sense | ATAGGTCGACTTAACCATGGGAAACGAACCAACAAATAATAATG | *dcd2_Dicty_* |
| dcd2_Rev_Smal | Antisense | TAGCCCGGGTTAGTGATGGTGATGGTGATGAGATCTGG | *dcd2_Dicty_* |
| pREP_3X_For1 | Sense | GAGGAATCCTGGCATATCATC | pREP3X vector upstream of multiple cloning site |
| pREP_3X_Rev1 | Antisense | CGTAATATGCAGCTTGAATGGGC | pREP3X vector downstream of multiple cloning site |

References

Baba T, Ara T, Hasegawa M, Takai Y, Okumura Y, Baba M, Datsenko KA, Tomita M, Wanner BL, Mori H. 2006. Construction of *Escherichia coli* K-12 in-frame, single-gene knockout mutants: The keio collection. Mol Syst Biol. 2: 2006.0008.

Curtiss R3, Charamella LJ, Stallions DR, Mays JA. 1968. Parental functions during conjugation in *Escherichia coli* K-12. Bacteriol.Rev. 32(4 Pt 1): 320-348.

Lederberg EM and Lederberg J. 1953. Genetic studies of lysogenicity in *Escherichia coli.* Genetics. 38(1): 51-64.

Sánchez A, Sharma S, Rozenzhak S, Roguev A, Krogan NJ, Chabes A, Russell P. 2012. Replication fork collapse and genome instability in a deoxycytidylate deaminase mutant. Mol Cell Biol. 32(21): 4445-4454.
